# Stratified and geographic patterns of depression and anxiety in Mozambican women: Mental health policy implications

**DOI:** 10.1371/journal.pone.0356265

**Published:** 2026-08-19

**Authors:** Md Salek Miah

**Affiliations:** Department of Statistics, Shahjalal University of Science and Technology (SUST), Sylhet, Bangladesh; Independent, UNITED STATES OF AMERICA

## Abstract

**Objective:**

To identify the risk factors of depression and anxiety among ever-married Mozambican women, including rural–urban, internet-use, and province- wide geographic inequalities, and to inform mental health policy.

**Methodology:**

This study used cross-sectional design and 13,183 ever-married women aged 15−49 years from the nationally representative Mozambique Demographic and Health Survey (MDHS) 2022−2023. The outcome variables were assessed using the Patients Health Questionnaire-9 (PHQ-9), and Generalized Anxiety Disorder-7 (GAD-7), with score (>=10) indicating depression and anxiety respectively. Survey-weighted multivariate logistic regression models were used to determine factors associated with anxiety and depression, and results were presented as adjusted odds ratios (aORs) with corresponding 95% confidence intervals (95% CIs).

**Findings:**

Among all women, 11% reported anxiety symptoms, and 10% reported depressive symptoms. Women aged 35–39 years associated the highest odds of anxiety (aOR = 2.06, 95% CI: 1.48–2.87) and depression (aOR = 1.49, 95% CI: 1.07–2.08). Additionally, women aged 45–49 years showed the second-highest odds of anxiety (aOR = 1.58, 95% CI: 1.12–2.23), while the second-highest odds of depressive symptoms were also observed among women aged 45–49 years (aOR = 1.32, 95% CI: 0.92–1.89), compared with women aged 15–19 years. Age at first sex (<18 years) was significantly associated with anxiety (aOR = 1.66, 95% CI: 1.25–2.19) and depression (aOR = 1.60, 95% CI: 1.17–2.18) compared to aged ≥18 years. Women participation in decision making autonomy significantly associated with lower odds of anxiety (aOR = 0.67, 95% CI: 0.54–0.83) and depression (aOR = 0.65, 95% CI: 0.53–0.81). Household asset ownership of (aOR = 0.76, 95% CI: 0.62–0.92), and improved sanitation facilities associated with lower odds for anxiety (aOR = 0.76, 95% CI: 0.60–0.98) only. While, improved household materials (aOR = 1.37, 95% CI: 1.07–1.76) and internet use (aOR = 1.46, 95% CI: 1.07–1.99) were associated with higher anxiety. Geographically, women Cabo Delgado and Zambézia, Nampula consistently associated with higher odds while Gaza associated with lower odds of both anxiety and depression compared to Maputo City respectively.

**Conclusions:**

Targeted mental health symptoms interventions should prioritize aged 35–49 years and high burden provinces such as Nampula strengthening women’s decision-making autonomy. Integrating routine mental health symptoms screening into reproductive and women’s health services may help decrease anxiety and depression among Mozambican women.

## 1. Introduction

Mental health symptoms are considered as the major causes of the global burden of disease [1]. In 2021, more than 359 million individuals diagnosed with depression and anxiety symptoms worldwide [2–4]. Depression and anxiety symptoms are associated with poor quality of life, reduced productivity, and a higher burden of comorbid physical conditions [5,6]. Biological, social and economic factors such as hormonal changes, events of reproductive and sociocultural pressures make women of reproductive age especially vulnerable [7]. Identifying determinants of anxiety and depression symptoms is very crucial for informing effective prevention and interventions roles [8,9].

Depression and anxiety are disparity associated with women of reproductive ages in low- and middle-income nations (LMICs) around the globe due to the inadequate health resources to support mental health service and limited access [10,11]. In many low-resource settings, the burden of anxiety and depression symptoms is worsened by insufficient mental health policies and execution outlines, insistent mental health symptoms, scarcities of trained mental health professionals, and limited accessibility of focused mental health facilities [12–14]. One Previous findings revealed internet use associated with mental health symptoms among women of reproductive age, with internet users showing lower odds of anxiety and depression symptoms than non-internet-users [13].

In Mozambique, mental health symptoms such as anxiety and depression symptoms are limited explored, a post-conflict nation facing acute socioeconomic disparity [15–18]. Available health research in Mozambique has mostly explored on maternal and child health, infectious diseases, and nutritional outcomes, while reproductive aged mental health symptoms remain a relatively limited explored area despite its significant suggestions for women’s health and well-being [18,19]. Considering the demographic heterogeneity of the country, with both urban and rural differences, it is essential to comprehend the problem of depression and anxiety symptoms in women to provide the context-specific interventions [10,20]. A nationally representative study will provide it possible to derive some generalizable knowledge, which can inform health policy, resource distribution, and interventions in Mozambique [21].

Previous findings revealed several determinants of anxiety and depression symptoms among women of reproductive age, educational status, marital status, household wealth status, reproductive history, attitude towards the justification of intimate partner violence (IPV), and access to media and health information, which may improve mental health literacy and help timely healthcare-seeking [1,22–24]. Recent findings have revealed that there is an association between internet use and the mental health symptoms of women in their reproductive years. Since the availability of the internet continues to increase in low- and middle-income countries, it has become essential to understand how the internet use associates with women’s anxiety and depression symptoms [25].

As an illustration, research has showed that education, early marriage, multiple pregnancies, and attitude towards the IPV are always associates with higher odds of depression symptoms [18]. Nevertheless, the majority of the studies have either concentrated on a set of variables or used simple regression methods without a methodological approach without using Directed Acyclic Graph (DAG), conceptual, theoretical, statistical driven approach such as forward or backward model selection to estimate potential confounders were included in the multivariate analysis [15,26]. Moreover, the limited number of studies have explored stratified disparities by place of residence such as rural-urban or internet access even though urbanization and exposure to the internet associated with mental health symptoms [13,27,28]. First, existing literature on analytical framework often lacks of systematic methods for confounder selection, fitting best models by evaluating Akaike Information Criteria (AIC), Bayesian Information Criteria (BIC), and Likelihood Ratio Estimators Test (LRT) and therefore excluding rigorous statistical, conceptual, theoretical approach for fitting models through significant confounding or mediating variables [29–31]. Second, limited evidence exists regarding geographic and internet access disparities in depression and anxiety, which indicates potential difference social connectivity, information access, and health care access availability [20,32]. Third, the provincial level of geographic inequalities of mental health symptoms prevalence remained limited explored in Mozambique, indicating a critical knowledge and evidence gaps in understanding regional disparities [17,18].

These gaps need to be addressed to provide an evidence-based policy to progress Sustainable Development Goals (SDGs), and Millenium Developments Goals (MDGs). This paper will address these gaps through most recent nationally representative Mozambique Demographic and Health Survey (MDHS) 2022−23 data. This study implemented an inclusive analytical outline, including a stepwise variable selection methods, survey-weighted multivariable logistic regression, descriptive geographic analysis, and place of residence wise stratified analyses to examine depression and anxiety among women of reproductive age in Mozambique. By integrating sociodemographic, reproductive, household, and environmental covariates, the study offers a wide-ranging evaluation of mental health symptoms determinants. This methodological outline provides a practical framework for other LMICs seeking to generate context-specific findings to inform data-driven maternal mental health symptoms policies and targeted interventions. Based on these outlines and previous literature, we examined these factors as potential association with anxiety and depression symptoms rather than implying causal relationships. This study aimed to examine depression and anxiety among ever-married women in Mozambique by:

1. Identifying associated factors using survey-weighted multivariable logistic regression.
2. Exploring rural–urban inequalities with stratified survey-weighted models.
3. Assessing disparities by internet use (users vs. non-users) through stratified analyses.
4. Mapping provincial-level prevalence using descriptive geographic analysis.
5. Providing evidence-based policy to enhance maternal and reproductive health policies for improving women’s mental health symptoms in Mozambique.

Findings from this study are aligned with the United Nations Sustainable Development Goals (SDGs), particularly SDG 3, which emphasizes good health and well-being, and targets reducing premature mortality from non-communicable diseases and promoting mental health symptoms. By identifying population subgroups at higher risk and highlighting modifiable factors, this research can guide policies and programs aimed at improving mental health symptoms, reducing health inequalities, and advancing Mozambique’s progress toward achieving the SDGs.

## 2. Methodology

### 2.1. Study design and data source

This investigation extracted data from the Mozambique Demographic and Health Survey (MDHS), 2022–2023, which is a nationally representative cross-sectional survey conducted from July 2022 to March 2023 [33]. This study was conducted in accordance with strengthening the reporting of observational Studies in epidemiology (STROBE) guidelines for observational research.

### 2.2. Sampling design and sample size

The survey employed a stratified, two-stage sampling design to provide estimates at the national, provincial, and urban–rural levels. The sampling frame used was based on the 2017 National Census. Initially, 619 enumeration areas (232 urban, 387 rural) were selected by probability proportional to size. Eight districts in Cabo Delgado were excluded due to security concerns. Secondly, in each enumeration area, 26 households were selected at random, totaling 14,250 households after two clusters were not surveyed due to inaccessibility. All women aged 15–49 years in selected households were eligible. Of 13,976 eligible women, 13,183 completed the interview (94% response rate). The study sample included ever-married women aged 15–49 years, regardless of their pregnancy status or reproductive history. Thus, the analyses examined anxiety and depressive symptoms among women of reproductive age rather than being restricted to pregnant or postpartum women. Therefore, this study used data from13,183 ever-married women aged 15–49 weighted to represent reproductive-aged women nationally [33].The study area is presented in S1 Fig.

### 2.3. Variables

#### 2.3.1. Outcomes variable.

Anxiety and depression symptoms were evaluated using the Generalized Anxiety Disorder-7 (GAD-7) and Patient Health Questionnaire-9 (PHQ-9), respectively. A cut-off score of ≥10 on each scale was used to classify participants as having clinically significant symptoms. The outcome variable anxiety, and depression were classified as “yes” if participants scored ≥10 on the PHQ-9 and GAD-7, respectively, and “no” otherwise. Both instruments have demonstrated strong internal consistency in previous research, with Cronbach’s alpha values ranging from 0.89–0.92 for the GAD-7 and 0.86–0.89 for the PHQ-9 [32].

#### 2.3.2. Covariates.

All covariates were selected from the standardized MDHS 2022–2023 questionnaire and household datasets. No additional data collection tools or exterior assessment tools were used. Based on previous literature and the availability of variables in the DHS dataset, a range of socio-demographic, reproductive, household, and contextual factors were included. Women’s age was categorized as 15–19, 20–24, 25–29, 30–34, 35–39, 40–44, and 45–49 years. Age at first birth was classified as early (10–17 years), normal (18–24 years), and late (≥25 years). Age at first sex categorized as <18 and ≥18 years, while age at cohabitation was grouped as <18, 18–20, and ≥21 years. Educational status for women and her husbands was categorized as below primary, secondary incomplete, and secondary or higher. Women employment, current employment, current pregnancy status, residing status, and current sexual abstinence were categorized as binary variable (“Yes”/” No”). Pressure to become pregnant was evaluated using the MDHS questionnaire, which asked whether women stated suffering pressure from their husband/partner to become pregnant (“Yes”/” No”). Additionally, antenatal care attendance was categorized as <4 visits and (≥4 visits). Women’s decision-making autonomy was derived from DHS questions regarding participation in decisions about their own healthcare, major household purchases, and visits to family or relatives. Women who participated alone or jointly with their partner in at least one of these decisions were classified as having autonomy (“Yes”), while those who did not participate in any decision were classified as having no autonomy (“No”). Attitude towards the justification of intimate partner violence (IPV) was derived from DHS items assessing whether a husband is justified in beating his wife under specific circumstances (e.g., going out without telling him, neglecting children, arguing with him, refusing sex, or burning food). Women who justified wife-beating in at least one situation were classified as “Yes”, while those who did not justify wife-beating in any situation were classified as “No”. Household asset ownership was constructed using ownership of selected household assets available in the DHS dataset (e.g., refrigerator, bicycle, or motorcycle,). Households possessing at least one asset were categorized as “Yes”, whereas households with none of the listed assets were categorized as “No”. Media access was derived from respondents’ exposure to mobile phone, newspapers/magazines, radio, and television. Women who reported access to at least one form of media were categorized as having media access (“Yes”), while those reporting no exposure to any media source were categorized as having no media access (“No”), and internet use was categorized as “Yes” if the respondent had used the internet in the previous 12 months and “No” otherwise. Reproductive characteristics included number of children (none, 1–2, ≥ 3), birth size (small vs. average/large), preterm birth (preterm vs. full-term), pregnancy history (single vs. multiple pregnancies), pregnancy loss (none, one loss, two or more losses), number of unions (once vs. more than once), and menstruation within the last six weeks (yes/no). Household and environmental variables included wealth status (poor, middle, rich), household size (1–3 vs. ≥ 4 members), sex of household head (male/female), water access (improved/unimproved), sanitation facility (improved/unimproved), and household materials (improved/unimproved), based on standard DHS classifications. Place of residence was categorized as urban or rural. Religion was categorized as Catholic, Islam, Zion, Evangelical/Pentecostal, and Other, according to the standardized MDHS 2022–2023 classifications [14,28,34–36].

### 2.4. Confounder selection process for adjusting in model

Potential confounder for depression and anxiety was identified through a systematic, three-step selection process:

1) Bivariate analyses using chi-square tests were conducted for all candidate variables, with a liberal significance threshold of p < 0.25 to minimize exclusion of potentially relevant predictors [37].
2) Second, univariate logistic regression models were used to assess effect sizes, prioritizing variables with clinically meaningful changes in odds ratios (≥10%) [37].
3) Finally, variables were evaluated for conceptual relevance based on theoretical considerations and prior evidence in mental health research. This approach ensured that the final multivariable model included variables that were statistically robust, clinically significant, and theoretically justified.

These variables included in the final adjusted multivariable logistic regression models: women age, age at first sex, women education, employment, women decision making autonomy, wealth, household assets, currently pregnant, pregnancy history, pregnancy loss, attitude towards the justification of intimate partner violence (IPV), age at cohabitation, water access, sanitation facilities, household materials, media, internet use, province, religion, and place of residence.

### 2.5. Statistical analysis

Descriptive analyses were first conducted to summarize the characteristics of the study participants, with results presented as frequencies and percentages. This study performed three complementary analyses: (1) survey-weighted multivariable logistic regression to examine factors associated with depression and anxiety symptoms, (2) rural–urban stratified survey-weighted logistic regression models to explore potential geographic disparities, (3) survey-weighted logistic regression models were stratified by internet use (users vs. non-users) to assess inequalities in depression and anxiety determinants, and (4) geographic analyses to assess provincial-level inequalities in depression and anxiety prevalence.

Due to inequalities in model specification across analyses (Tables 3–5), reference categories for province were not consistent across all models. Specifically, Maputo City was used as the reference category in the main multivariable models (Table 3 and Table 4), while Niassa was used as the reference category in selected stratified or outcome-specific models (Table 5) to enhance interpretability across rural–urban comparisons. All analyses were weighted for clustering and stratification using provided survey weights. Associations of the covariates with the mental health outcomes were estimated by survey-weighted multivariable logistic regression. All results are shown as AORs with 95% CIs. All statistical analyses were performed using StataSE version 17 (StataCorp, USA), while geographic analyses and figure generation were conducted in R Studio version 4.5.1. Provincial administrative boundary shapefiles were obtained from the Humanitarian Data Exchange (HDX) Common Operational Dataset (COD) for Mozambique and all maps were created by the authors using the R packages sf and ggplot2. Cases with missing values were excluded from advanced analyses.

### 2.6. Reproducibility

To improve transparency and reproducibility, the STATA and R scripts used for data management, statistical analyses, and figure generation are publicly available in a GitHub repository at https://github.com/muhammadsalek/mozambique-mental-health-analysis.

### 2.7. Model assumption

After developing the adjusted model, the Hosmer Lemeshow test was conducted to evaluate models’ goodness of fit, with a non-significant p-value (p = 0.05) indicating an adequate fit, and assessing discriminative ability used Receiver Operating Characteristic curve (AUCROC). Variance inflation factors (VIFs) were calculated to check for multicollinearity, with values above 10 indicating potential multicollinearity issues.

### 2.8. Ethical considerations

The Mozambique Demographic and Health Survey (MDHS) 2022–23 received ethical approval from the Mozambique National Health Research Council (NHRC) and the ICF Institutional Review Board. Permission to use the anonymized dataset for secondary analysis was granted by the **DHS Program**, and details available at The DHS Program – Protecting the Privacy of DHS Survey Respondents.

## 3. Results

### 3.1. Distribution of GAD-7 and PHQ-9 Item Responses

**Fig 1:**
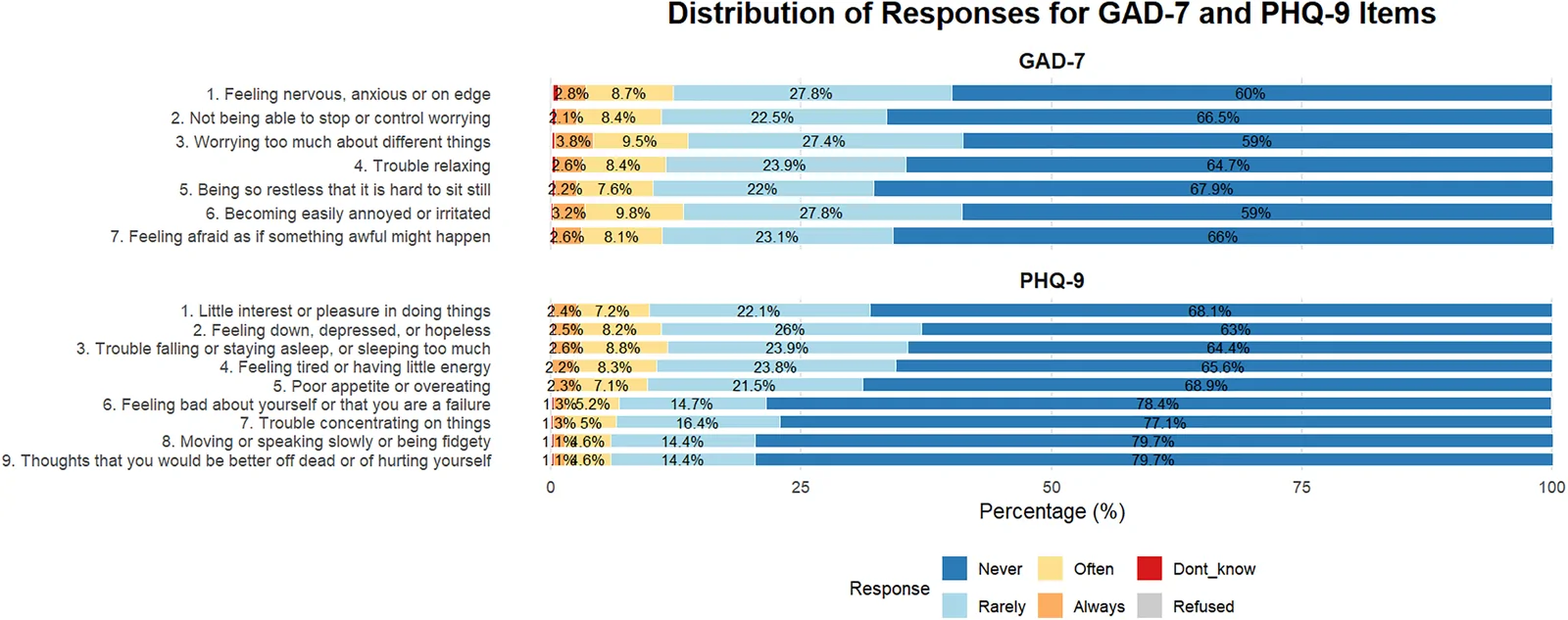
Distribution of responses for GAD-7 and PHQ-9.

Across both GAD-7 and PHQ-9 scales, “Never” was the most common response, ranging from 59%–68% for anxiety items and 63%–78% for depression items, including for severe symptoms such as suicidal thoughts. Symptom-showing responses (“Rarely,” “Often,” or “Always”) were comparatively less frequent (Fig 1, S1 Table).

### 3.2. Sociodemographic, reproductive, and household characteristics of the study population

The sample is mostly of adolescent and young women, the highest prevalence of which is 15–19 years (23.1%) and the lowest prevalence is 20–24 years (20.4%). Early life-course reproductive experiences are widespread, including a high prevalence who reported early age at first birth (35.2% at 10–17 years) and early cohabitation (<18 years: 38.5%), reflecting the high prevalence of early transition into motherhood and partnership. Women’s educational attainment was generally low, with 69.2% showing below primary education, 28.1% showing incomplete secondary education, and only 2.7% having completed secondary or higher education. Majority of women were currently employed (69.6%) and majority of the women had visited antenatal care for at least four visits (80.0%). The reproductive features showed that most women were pregnant (92.6%), and that many had multiple pregnancies (76.9%), felt pressured to become pregnant (88.3%) and had experienced some form of pregnancy loss (89.9% reporting one pregnancy loss). Desire for contraceptives seems low, as only 6.5% wanted more children. There are mixed patterns of decision-making autonomy and gender norms: 58.4% of women showed autonomy in decision making, but many (81.3%) showed attitude towards the justification of intimate partner violence (IPV) is under some circumstances, reflecting continued strong gender norm acceptance. Household conditions are moderate socioeconomic disadvantage, with 45.7% in poor wealth status, 61.2% in rural residence and more than half without improved water source (62.9%) and media access (64.7%). Despite this, internet use is fairly high (80.0%), indicating that this is mobile internet usage. Geographically, he highest concentration in Nampula (23.2%) (Table 1).

**Table 1:** Descriptive statistics of study variables among Mozambican reproductive-aged women (Weighted n = 13,183).

Variable	Weighted N (%)
**Age of woman**	
15–19	3050 (23.1)
20–24	2693 (20.4)
25–29	2195 (16.7)
30–34	1577 (12.0)
35–39	1486 (11.3)
40–44	1171 (8.9)
45–49	1011 (7.7)
**Age at first birth**	
10–17 (Early)	4637 (35.2)
18–24 (Normal)	4718 (35.8)
≥ 25 (Late)	3828 (29.0)
**Age at first sex**	
≥ 18 years	11186 (84.9)
< 18 years	1997 (15.1)
**Age at cohabitation**	
Mild (18–20)	5606 (42.5)
Older (21+)	2503 (19.0)
< 18 years	5074 (38.5)
**Education of woman**	
Below Primary	9123 (69.2)
Secondary Incomplete	3709 (28.1)
Secondary+ Higher	352 (2.7)
**Husband’s education**	
Below Primary	3313 (25.1)
Secondary Incomplete	5449 (41.3)
Secondary+ Higher	4421 (33.5)
**Maternal employment**	
Yes	9178 (69.6)
Not	4005 (30.4)
**Antenatal care (≥4 visits)**	
Yes	10551 (80.0)
No	2632 (20.0)
**Number of children**	
None	5573(42.3)
1–2	3250 (24.7)
3+	4361 (33.1)
**Birth size**	
Average or Large	8968 (68.0)
Small	4215 (32.0)
**Preterm birth**	
Preterm (<9 months)	12887 (97.8)
Full- Term	296 (2.2)
**Currently pregnant**	
Yes	12211 (92.6)
No	972 (7.4)
**Pregnancy history**	
Multiple pregnancy	10133 (76.9)
Single pregnancy	3050 (23.1)
**Pressure to become pregnant**	
Yes	11641 (88.3)
No	1542 (11.7)
**Pregnancy loss**	
One loss	11858 (89.9)
Two or more losses	996 (7.6)
None	329 (2.5)
**Residing status**	
Yes	1418 (10.8)
No	11765 (89.2)
**Currently abstaining from sexual intercourse**	
Yes	11185 (84.8)
No	1998 (15.2)
**Number of unions**	
More than once	8222 (62.4)
Once	4961 (37.6)
**Menstruated in last 6 weeks**	
Yes	4373 (33.2)
No	8810 (66.8)
**Decision making Autonomy**	
Yes	7703 (58.4)
No	5480 (41.6)
**Want more children**	
Yes	857 (6.5)
No	12326 (93.5)
**IPV justification**	
Yes	10719 (81.3)
No	2464 (18.7)
**Sanitation Facilities**	
Improved	9146 (69.4)
Unimproved	4037 (30.6)
**Water source**	
Improved	4896 (37.1)
Unimproved	8287 (62.9)
**Place of residence**	
Urban	5120 (38.8)
Rural	8063 (61.2)
**Province**	
Niassa	861 (6.5)
Cabo Delgado	705 (5.3)
Nampula	3064 (23.2)
Zambézia	2193 (16.6)
Tete	1314 (10.0)
Manica	909 (6.9)
Sofala	909 (6.9)
Inhambane	555 (4.2)
Gaza	670 (5.1)
Maputo	1347 (10.2)
Cidade de Maputo	655 (5.0)
**Religion**	
Catholic	3920 (29.7)
Islam	2715 (20.6)
Zion	1595 (12.1)
Evangelical/Pentecostal	3721 (28.2)
Other	1233 (9.4)
**Currently working**	
Yes	9178 (69.6)
No	4005 (30.4)
**Wealth Status**	
Middle Class	4783 (36.3)
Rich	2372 (18.0)
Poor	6029 (45.7)
**Household size**	
1–3 members	10836 (82.2)
4 or more members	2347 (17.8)
**Household head sex**	
Male	9001 (68.3)
Female	4182 (31.7)
**Household assets**	
Yes	6447 (48.9)
No	6736 (51.1)
**Media access**	
Yes	4652 (35.3)
No	8531 (64.7)
**Internet use**	
Yes	10547 (80.0)
No	2636 (20.0)
**Household materials**	
Improved	6178 (46.9)
Unimproved	7005 (53.1)

### 3.3. Weighted prevalence of anxiety and depressive symptoms across participant characteristics

In general, the survey weighted descriptive prevalence of mental health symptoms was high, with 11.09% (95% CI 9.8–12.4) reporting anxiety symptoms and 10.14% (8.9–11.5) reporting depressive symptoms. Descriptive prevalence rates higher for people aged 20–24 years for anxiety (21.23%) and depression (22.57%) and lower with age, with the lowest rates among females aged 40–44. Women with early reproductive transitions showed constantly higher descriptive prevalence of mental health symptoms, mostly among those whose first birth occurred at ages 10–17 years (38.18% anxiety; 35.83% depression) and those with sexual debut before 18 years (~77% for both outcomes). The majority of symptom burden (anxiety 75.06% and depression 78.00%) were showed in women with lower education (below primary education) and higher education (secondary+higher) showed significantly lower prevalence. Other notable findings related to reproductive health and psychosocial symptoms. Moreover, high gender norms with regard to low decision-making autonomy and high acceptance of attitude toward the justification of intimate partner violence (IPV), respectively, were reported by a significant proportion of women with symptoms (64.09% and ≈22%, respectively) (Table 2).

**Table 2:** Survey-weighted prevalence of anxiety and depressive symptoms among Mozambican women.

Variables	Anxiety symptoms (>= 10) % (95% CI)	Depressive symptoms (>= 10) % (95% CI)
Overall	11.09 (9.8, 12.4)	10.14 (8.9, 11.5)
**Women age**		
15-19	17.59 (15.01,20.51)	19.72 (16.95,22.81)
20-24	21.23 (18.5,24.26)	22.57 (19.33,26.17)
25-29	18.54 (15.95,21.44)	18.00 (15.28,21.09)
30-34	12.03 (9.6, 14.8)	11.45 (9.33,13.96)
35-39	14.51 (12.3,17.04)	12.52 (10.49,14.88)
40-44	7.4 (5.8, 9.3)	7.12 (5.60,9.01)
45-49	8.6 (6.7, 11.01)	8.60 (6.66, 11.83)
**Age at first Birth**		
10–17 (Early)	38.18 (33.8, 42.6)	35.83 (31.96, 39.89)
18–24 (Normal)	35.5 (31.8, 39.5)	36.95 (33.43, 49.62)
≥ 25 (Late)	26.2 (22.8, 29.8)	27.21 (23.80, 30.91)
**Age at first sex**		
< 18 years	76.64 (71.7, 80.88)	77.73 (72.52, 82.19)
≥ 18 years	23.35 (19.11, 28.21)	22.26 (17.80, 27.47)
**Age at cohabitation**		
Young (<18)	44.7 (40.49, 49.01)	42.75 (38.89, 46.69)
Mild (18–20)	21.88 (18.65, 25.48)	21.34 (18.04, 25.06)
Older (21+)	33.39 (29.74, 37.26)	35.90 (32.00, 39.99)
**Women Education**		
Below Primary	75.06 (70.86, 78.83)	78.00 (74.26,81.34)
Secondary incomplete	23.38 (19.79,27.39)	20.64 (17.38, 24.33)
Secondary+	1.55 (1.01,2.37)	1.35 (0.85, 2.13)
**Husband Education**		
Below primary	27.35 (22.22- 33.15)	29.94 (24.21,36.38)
Below Secondary	42.40 (36.84,48.16)	42.54 (36.73,48.57)
Secondary+Higher	30.24 (25.17,35.84)	27.50 (22.29, 33.41)
**Employment**		
Not	78.77 (75.26,24.73)	80.54 (77.15,83.54)
Yes	21.22 (18.08,24.73)	19.45 (16.45, 22.84)
**ANC visit**		
< 4	82.18 (78.99,84.98)	81.56 (78.05, 84.62)
>=4	17.81 (15.01,21.0)	18.43 (15.37,21.94)
**Number of children**		
None	21.68 (18.51, 25.23)	22.91 (19.68,26.49)
1-2	33.76 (30.10, 37.64)	33.11 (29.36, 37.10)
3+	44.54 (40.78, 48.36)	43.96 (40.19, 47.81)
**Birth Size**		
Small	68.60 (64.24- 72.66)	67.74 (63.08,69.48)
Average or Large	31.39 (27.33- 35.75)	32.25 (27.92,36.91)
**Preterm birth**		
Term (>=9 months)	97.69 (95.95, 98.69)	97.79 (95.91,98.82)
Preterm (<9 months)	2.30 (1.30, 4.04)	2.20 (1.17,4.08)
**Currently Pregnant**		
No	90.46 (88.13, 92.38)	90.07 (87.71,92.01)
Yes	9.5 (7.6, 11.8)	9.92 (7.98,12.28)
**Pregnancy History**		
Single pregnancy	81.16 (77.63, 84.24)	79.96 (76.50, 83.02)
Multiple pregnancy	18.83 (15.75, 22.36)	20.03 (16.97,23.49)
**Pressure to be pregnant**		
No	88.16 (84.76, 90.89)	90.84 (87.93,93.11)
Yes	11.83 (9.1, 15.2)	9.15 (6.88, 12.06)
**Pregnancy loss**		
None	92.35 (90.2,94.0)	93.06 (91.19, 94.55)
One loss	4.9 (3.5, 6.7)	4.84 (3.56, 6.55)
Two or more losses	2.7 (1.8, 3.9)	2.09 (1.42, 3.06)
**Residing Status**		
No	7.45 (5.46, 10.07)	7.76 (5.56,10.73)
Yes	92.55 (89.92, 94.53)	92.23 (89.26, 94.43)
**Currently Abstaining**		
No	86.94 (84.12, 89.32)	86.42 (83.25, 89.07)
Yes	13.06 (10.67, 15.87)	13.57 (10.92,16.74)
**Number of Unions**		
Once	56.29 (52.35,60.14)	56.93 (53.08, 60.69)
More than once	43.71(39.85,47.64)	43.06 (39.30, 46.91)
**Menstruated last 6 weeks**		
No	32.51 (28.83,36.40)	31.33 (27.41, 35.53)
Yes	67.49 (63.59,71.16)	68.66 (64.46, 72.58)
**Decision Making Autonomy**		
No	64.09 (58.86,69.00)	66.69 (61.93,71.13)
Yes	35.91 (30.99, 41.13)	33.30 (28.86, 38.06)
**Want More Children**		
No	6.29 (3.81, 10.19)	6.05 (3.81, 9.49)
Yes	93.71 (89.80,96.18)	93.94 (90.50, 96.18)
**IPV Justification**		
No	77.73 (73.17,81.70)	77.81 (72.67, 82.22)
Yes	22.27 (18.29,26.82)	22.18 (17.77, 27.32)
**Sanitation Facilities**		
Unimproved	74.39 (69.96,78.37)	74.26 (69.71,78.34)
Improved	25.60 (21.62,30.03)	25.73 (21.65,30.28)
**Water Access**		
Unimproved	47.36 (40.62,54.20)	48.75 (41.65, 55.90)
Unimproved	52.63 (45.79,59.37)	51.24 (44.09, 58.34)
**Place of Residence**		
Urban	37.92 (32.15,44.05)	36.53 (30.33, 43.22)
Rural	62.07 (55.94, 67.84)	63.46 (56.77,69.66)
**Province**		
Niassa	16.65 (1.16,2.37)	1.91 (1.20,3.05)
Cabo Delgado	4.40 (2.56,7.45)	6.85 (4.49, 10.31)
Nampula	59.67 (53.84,65.24)	55.47 (48.78, 61.96)
Zambuzia	13.68 (9.77,18.82)	16.26 (10.88, 23.61)
Tete	4.62 (3.23,6.58)	2.86 (1.79,4.45)
Manica	1.55 (0.93,2.57)	1.58 (0.89,2.79)
Sofala	4.48 (3.28,6.09)	6.86 (5.32, 8.81)
Inhambane	0.61 (0.32,1.17)	1.34 (0.87, 2.05)
Gaza	0.48 (0.26,0.86)	0.46 (0.26,0.82)
Maputo	7.36 (4.73,11.27)	5.21 (3.12, 8.56)
Cidade de maputo	1.44 (1.00,2.08)	1.14 (0.79,1.66)
**Religion**		
Catholic	35.35 (29.54,41.62)	36.19 (30.24, 42.59)
Islam	33.18 (27.11,39.88)	33.10 (26.93, 39.91)
Zion	5.31 (4.00,7.00)	5.36 (3.94,7.26)
Evangelical/Pentecostal	18.10 (14.58,22.25)	18.30 (14.97, 22.17)
Other	8.04 (6.06,10.59)	7.03 (5.03,9.76)
**Currently working**		
No	78.77 (75.26,81.91)	80.54 (77.15,83.54)
Yes	21.22 (18.08,24.73)	19.45 (16.45, 22.84)
**Wealth Status**		
Poor	45.49 (39.42,51.68)	47.74 (41.34, 54.22)
Middle class	15.66 (12.28,19.77)	15.37 (12.17, 19.23)
Rich	38.84 (33.12,44.88)	36.87 (30.85,43.33)
**Household Size**		
1–3 members	18.75 (15.93, 21.93)	19.03 (15.89,22.63)
4 or more members	81.24 (78.06, 84.06)	80.96 (77.36,84.10)
**Household head sex**		
Male	70.55 (66.87, 73.97)	68.17 (64.36,71.76)
Female	29.44 (66.87,73.97)	31.82 (28.23,35.63)
**Household assets**		
No	61.81 (57.65,65.80)	61.04 (57.06,64.87)
Yes	38.18 (34.19,42.34)	38.95 (35.12,42.93)
**Media Access**		
No	43.08 (37.69,48.63)	46.06 (40.68,51.54)
Yes	56.91 (51.36,62.30)	53.93 (48.45,59.31)
**Internet use**		
No	87.05 (83.91, 89.65)	89.38 (86.35, 91.81)
Yes	12.94 (10.34,16.08)	10.61 (8.18,13.64)
**Household materials**		
Unimproved	55.49 (50.18, 60.67)	58.08 (52.42,63.53)
Improved	44.50 (39.32, 49.81)	41.91 (36.46, 47.57)

### 3.4. Factors associated with anxiety and depressive symptoms: Multivariable logistic regression

**Fig 2:**
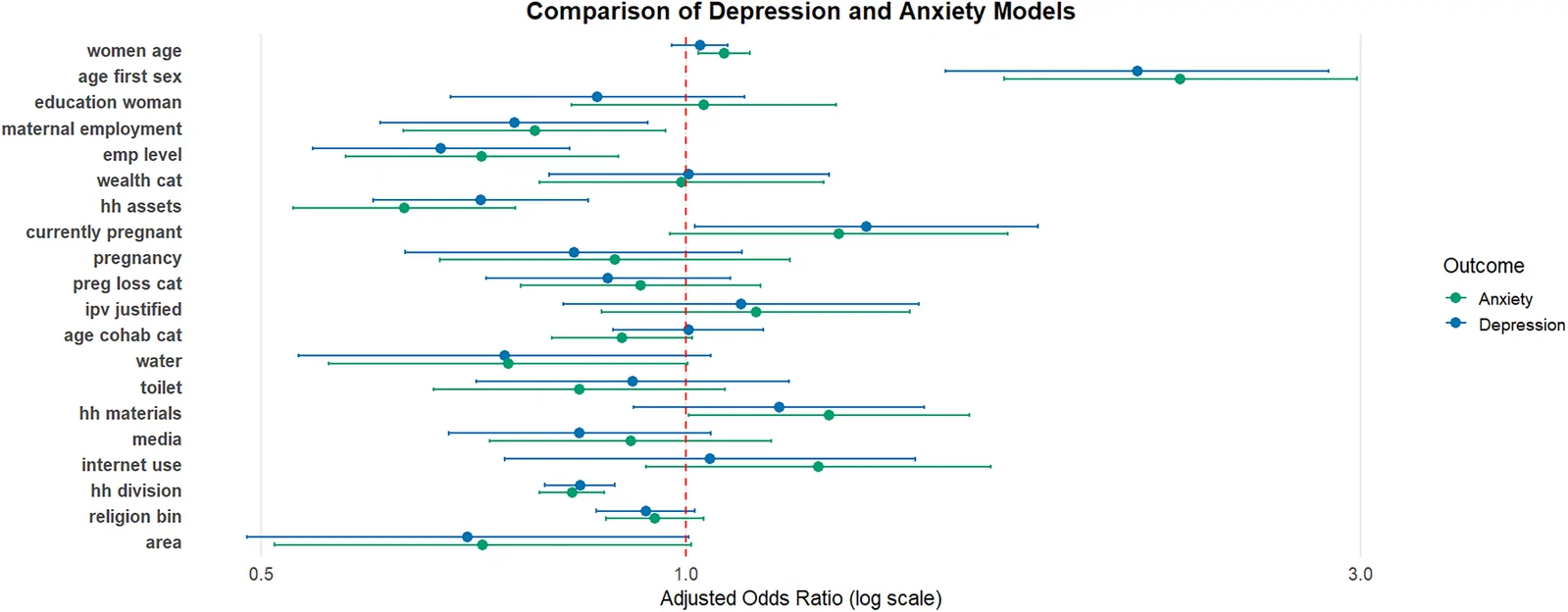
Forest plot of adjusted odds ratios (AORs) for anxiety and depression by key risk factors.

Compared with women aged 15–49 years (reference group), women aged 35–39 years significantly associated with more than twice the odds of anxiety (aOR = 2.06, 95% CI 1.48–2.87; p < 0.001) and significantly higher odds of depression symptoms (aOR = 1.49, 95% CI 1.07–2.08; p = 0.020). Similarly, women aged 45–49 years associated with higher odds of anxiety (aOR = 1.58, 95% CI 1.12–2.23; p = 0.009). Age at first sex was significantly associated with higher odds of both anxiety (AOR = 1.66, 95% CI 1.25–2.19; p < 0.001) and depression symptoms (aOR = 1.60, 95% CI 1.17–2.18; p = 0.003). However, participating in decision making autonomy associated with lower odds of anxiety (aOR = 0.67, 95% CI 0.54–0.83; p < 0.001) and depression (aOR = 0.65, 95% CI 0.53–0.81; p < 0.001) compared to no participation in decision making autonomy respectively. Likewise, ownership of household assets was associated with lower odds of anxiety only (aOR = 0.76, 95% CI 0.62–0.92; p = 0.005) compared to no household ownership. Among WASH indicators, improved sanitation facilities associated with 24% lower odds of anxiety (aOR = 0.76, 95% CI 0.60–0.98; p = 0.035) compared to unimproved sanitation facilities, while households with improved materials associated 37% higher odds of anxiety only (aOR = 1.37, 95% CI 1.07–1.76; p = 0.013) compared to unimproved household materials. Internet use also associated with 46% higher odds of anxiety only (aOR = 1.46, 95% CI 1.07–1.99; p = 0.018) compared to no internet use. Women in Nampula associated with substantial higher odds of both anxiety (aOR = 12.06, 95% CI 8.05–18.06; p < 0.001) and depression respectively (aOR = 9.02, 95% CI 5.34–15.23; p < 0.001) compared to Maputo Cidade province. While, Gaza province associated with substantially lower odds of both outcomes (anxiety: aOR = 0.23, 95% CI 0.11–0.49; p < 0.001; depression: aOR = 0.23, 95% CI 0.10–0.53; p = 0.001) respectively (Table 3, Fig 2).

**Table 3:** Survey-weighted multivariable logistic regression analysis of factors associated with anxiety and depressive symptoms among women (N = 13,183).

Variables	Anxiety symptoms (AOR [95% CI])	p-value	Depressive symptoms (AOR [95% CI])	p-value
**Women Age**				
15–19	Ref	–	Ref	–
20–24	1.28 [0.97, 1.69]	0.075	1.24 [0.94, 1.65]	0.134
25–29	1.43 [1.03, 1.97]	0.031	1.23 [0.90, 1.67]	0.192
30–34	1.41 [0.98, 2.02]	0.061	1.17 [0.82, 1.66]	0.396
35–39	2.06 [1.48, 2.87]	**<0.001**	1.49 [1.07, 2.08]	0.020
40–44	1.23 [0.81, 1.85]	0.328	1.03 [0.72, 1.47]	0.882
45–49	1.58 [1.12, 2.23]	0.009	1.32 [0.92, 1.89]	0.132
**Age at first sex**				
< 18 years	Ref	–	Ref	–
≥ 18 years	1.66 [1.25, 2.19]	<0.001	1.60 [1.17, 2.18]	0.003
**Women Education**				
Below Primary	Ref	–	Ref	–
Secondary Incomplete	1.05 [0.82, 1.35]	0.669	0.85 [0.65, 1.12]	0.254
Secondary+	0.82 [0.50, 1.35]	0.436	0.74 [0.40, 1.39]	0.353
**Employment**				
No	Ref	–	Ref	–
Yes	1.01 [0.81, 1.26]	0.922	0.93 [0.74, 1.16]	0.519
**Decision making Autonomy**				
No	Ref	–	Ref	–
Yes	0.67 [0.54, 0.83]	<0.001	0.65 [0.53, 0.81]	**<0.001**
**Wealth**				
Poor	Ref	–	Ref	–
Middle Class	0.92 [0.65, 1.31]	0.65	0.88 [0.62, 1.24]	0.456
Rich	1.00 [0.61, 1.65]	0.986	1.08 [0.66, 1.75]	0.769
**Household assets**				
No	Ref	–	Ref	–
Yes	0.76 [0.62, 0.92]	0.005	0.84 [0.69, 1.02]	0.077
**Currently pregnant**				
No	Ref	–	Ref	–
Yes	1.29 [0.95, 1.76]	0.010	1.35 [1.00, 1.83]	0.052
**Pregnancy History**				
Single	Ref	–	Ref	–
Multiple pregnancy	0.95 [0.67, 1.32]	0.745	0.85 [0.62, 1.16]	0.294
**Pregnancy loss**				
None	Ref	–	Ref	–
One loss	0.71 [0.49, 1.04]	0.075	0.74 [0.53, 1.03]	0.076
Two or more losses	1.18 [0.72, 1.92]	0.512	0.87 [0.52, 1.46]	0.604
**IPV justification**				
No	Ref	–	Ref	–
Yes	0.98 [0.72, 1.33]	0.904	0.96 [0.69, 1.33]	0.794
**Age at cohabitation**				
≤ 17 years	Ref	–	Ref	–
Mild (18–20)	1.04 [0.83, 1.31]	0.708	1.12 [0.90, 1.40]	0.3
Older (21+)	0.87 [0.68, 1.12]	0.271	1.11 [0.85, 1.45]	0.439
**Water Access**				
Unimproved	Ref	–	Ref	–
Improved	0.86 [0.63, 1.18]	0.356	0.84 [0.58, 1.21]	0.343
**Sanitation Facilities**				
Unimproved	Ref	–	Ref	–
Improved	0.76 [0.60, 0.98]	0.035	0.85 [0.65, 1.11]	0.231
**Household materials**				
Unimproved	Ref	–	Ref	–
Improved	1.37 [1.07, 1.76]	0.013	1.25 [0.96, 1.62]	0.096
**Media Access**				
No	Ref	–	Ref	–
Yes	0.97 [0.76, 1.23]	0.793	0.88 [0.71, 1.10]	0.263
**Internet use**				
No	Ref	–	Ref	–
Yes	1.46 [1.07, 1.99]	0.018	1.16 [0.80, 1.68]	0.421
**Province**				
Maputo Cidade	Ref	–	Ref	–
Cabo Delgado	3.47 [1.83, 6.57]	**<0.001**	4.99 [2.67, 9.33]	**<0.001**
Nampula	12.06 [8.05, 18.06]	**<0.001**	9.02 [5.34, 15.23]	**<0.001**
Zambézia	3.03 [1.82, 5.02]	<0.001	3.06 [1.59, 5.86]	0.001
Tete	1.57 [0.95, 2.60]	0.080	0.90 [0.46, 1.78]	0.765
Manica	0.59 [0.31, 1.12]	0.107	0.60 [0.28, 1.32]	0.204
Sofala	1.96 [1.17, 3.28]	0.011	3.04 [1.68, 5.50]	**<0.001**
Inhambane	0.37 [0.17, 0.80]	0.012	0.90 [0.45, 1.81]	0.762
Gaza	0.23 [0.11, 0.49]	**<0.001**	0.23 [0.10, 0.53]	0.001
Maputo	1.87 [0.98, 3.56]	0.059	1.37 [0.63, 2.96]	0.427
**Religion**				
Catholic	Ref	–	Ref	–
Islam	1.05 [0.79, 1.40]	0.737	1.02 [0.74, 1.41]	0.897
Zion	1.06 [0.76, 1.49]	0.727	1.10 [0.74, 1.65]	0.641
Evangelical/Pentecostal	1.28 [0.91, 1.79]	0.158	1.25 [0.89, 1.76]	0.198
Other	1.48 [1.02, 2.16]	0.039	1.18 [0.78, 1.78]	0.432
**Place of Residence**				
Urban	Ref	–	Ref	–
Rural	0.93 [0.64, 1.34]	0.679	0.89 [0.60, 1.33]	0.583

### 3.5. Residence- and internet use–stratified analysis of factors associated with anxiety symptoms

**Fig 3:**
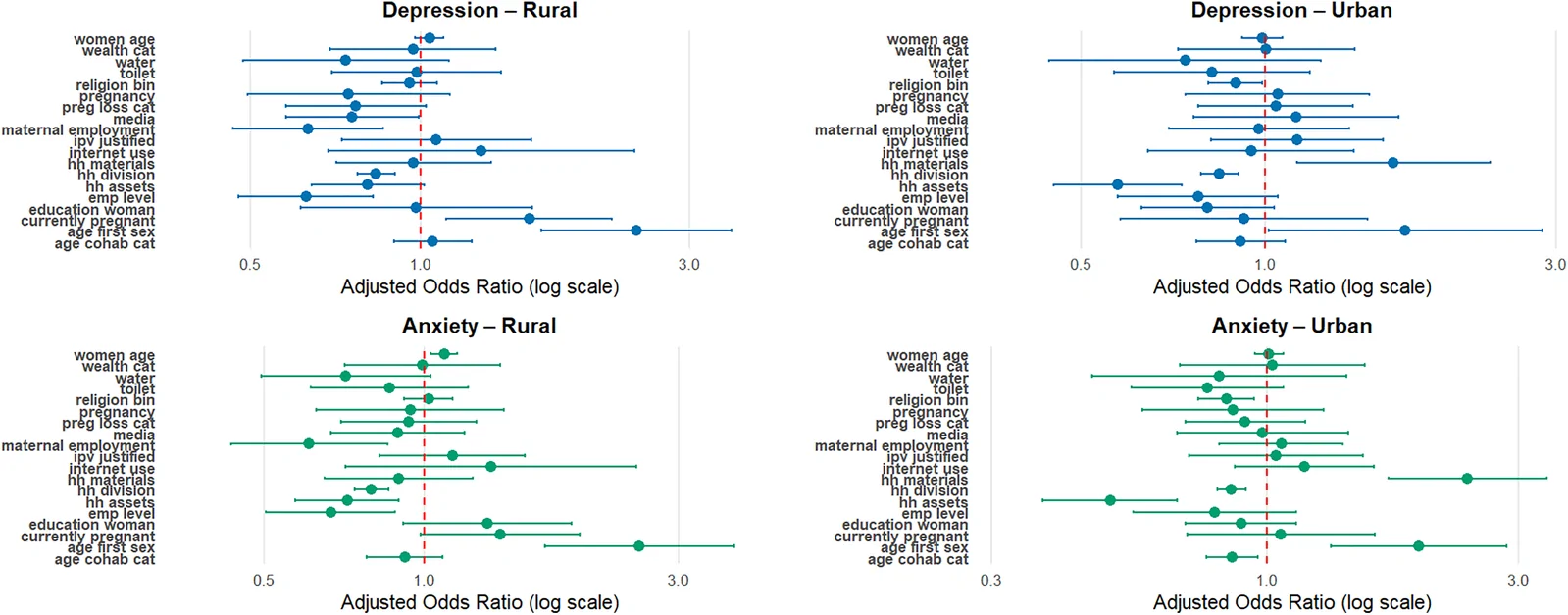
Survey-weighted associations of anxiety and depression with rural–urban residence.

Age was positively associated with anxiety symptoms, with women aged 35–39 years showing significantly higher odds compared to adolescents (15–19 years) in rural (aOR = 1.88, 95% CI: 1.20–2.94, p = 0.006), urban (aOR = 2.25, 95% CI: 1.36–3.72, p = 0.002), and non-internet-user’s groups (aOR = 2.21, 95% CI: 1.54–3.18, p < 0.001). Similarly, higher odds were also observed among women aged 45–49 years in rural (aOR = 1.86, 95% CI: 1.22–2.82, p = 0.004) and non-internet users (aOR = 1.68, 95% CI: 1.18–2.39, p = 0.004). Among internet users, significantly higher odds of anxiety were observed only in women aged 25–29 years (aOR = 2.21, 95% CI: 1.05–4.63, p = 0.036). Women initiating sexual activity at ≥18 years associated with higher odds of anxiety compared to those <18 years in rural (aOR = 1.88, 95% CI: 1.25–2.83, p = 0.002), urban (aOR = 1.44, 95% CI: 1.01–2.04, p = 0.043), and non-internet-user’s women (aOR = 1.82, 95% CI: 1.32–2.51, p < 0.001). However, decision-making autonomy was consistently associated with lower odds of anxiety in rural (aOR = 0.59, 95% CI: 0.45–0.79, p < 0.001) and non-internet-users’ women (aOR = 0.64, 95% CI: 0.51–0.82, p < 0.001). Women in the richest households associated with lower odds of anxiety among internet users (aOR = 0.14, 95% CI: 0.02–0.94, p = 0.043). Improved sanitation was associated with lower odds of anxiety among non-internet users (aOR = 0.68, 95% CI: 0.51–0.92, p = 0.011). While, improved household materials were associated with higher odds of anxiety among urban women (aOR = 2.43, 95% CI: 1.71–3.45, p < 0.001). Internet use was associated with higher odds of anxiety among rural women (aOR = 2.06, 95% CI: 1.00–4.22, p = 0.050). Compared to Maputo City, women in Nampula consistently had substantially higher odds of anxiety across rural (aOR = 15.36, 95% CI: 9.02–26.13, p < 0.001), urban (aOR = 8.44, 95% CI: 4.48–15.91, p < 0.001), and non-internet-users (aOR = 13.13, 95% CI: 8.43–20.45, p < 0.001). In contrast, women in Gaza associated with significantly lower odds of anxiety in rural (aOR = 0.25, 95% CI: 0.09–0.68, p = 0.007) and non-internet-users (aOR = 0.28, 95% CI: 0.12–0.66, p = 0.003). Evangelical/Pentecostal affiliation was associated with higher odds of anxiety in rural (aOR = 1.95, 95% CI: 1.21–3.14, p = 0.006) and non-internet users (aOR = 1.57, 95% CI: 1.04–2.37, p = 0.031), but lower odds in urban women (aOR = 0.53, 95% CI: 0.37–0.77, p = 0.001) (Table 4, Fig 3).

**Table 4:** Survey-weighted multivariable logistic regression of anxiety symptoms among women in Mozambique (N = 13,183) Use (Urban, N = 5,695 and Rural, N = 7,488).

Characteristic	Rural		Urban		No Internet		Internet	
	aOR (95% CI)	p	aOR (95% CI)	p	aOR (95% CI)	p	aOR (95% CI)	p
***Women’s age***								
***(ref: 15–19)***								
20–24	1.22 (0.84, 1.76)	0.296	1.38 (0.91, 2.10)	0.133	1.30 (0.96, 1.76)	0.088	1.25 (0.67, 2.33)	0.475
25–29	1.23 (0.81, 1.87)	0.339	1.72 (0.99, 2.99)	0.053	1.32 (0.93, 1.85)	0.116	2.21 (1.05, 4.63)	0.036
30–34	1.38 (0.87, 2.19)	0.169	1.32 (0.71, 2.44)	0.378	1.41 (0.95, 2.08)	0.086	1.30 (0.53, 3.17)	0.566
35–39	1.88 (1.20, 2.94)	0.006	2.25 (1.36, 3.72)	0.002	2.21 (1.54, 3.18)	<0.001	1.11 (0.45, 2.74)	0.823
40–44	1.37 (0.79, 2.35)	0.260	0.90 (0.50, 1.61)	0.713	1.29 (0.83, 2.02)	0.259	0.62 (0.23, 1.70)	0.355
45–49	1.86 (1.22, 2.82)	0.004	1.00 (0.52, 1.93)	0.992	1.68 (1.18, 2.39)	0.004	0.54 (0.17, 1.75)	0.307
***Age at first sex***								
***(ref: < 18 years)***								
≥ 18 years	1.88 (1.25, 2.83)	0.002	1.44 (1.01, 2.04)	0.043	1.82 (1.32, 2.51)	<0.001	1.05 (0.65, 1.70)	0.826
***Women’s education***								
***(ref: Below primary)***								
Secondary incomplete	1.36 (0.88, 2.12)	0.168	0.89 (0.68, 1.16)	0.390	1.03 (0.77, 1.38)	0.838	0.91 (0.52, 1.59)	0.733
Secondary+	0.78 (0.21, 2.91)	0.711	0.72 (0.42, 1.24)	0.241	0.43 (0.07, 2.54)	0.350	0.70 (0.35, 1.42)	0.324
***Employment***								
***(ref: No)***								
Yes	0.89 (0.63, 1.26)	0.509	1.14 (0.85, 1.53)	0.364	1.01 (0.78, 1.31)	0.942	1.24 (0.85, 1.81)	0.259
***Decision-making autonomy***								
***(ref: No)***								
Yes	0.59 (0.45, 0.79)	<0.001	0.80 (0.56, 1.14)	0.219	0.64 (0.51, 0.82)	<0.001	0.93 (0.59, 1.47)	0.751
***Household wealth***								
***(ref: Poor)***								
Middle	1.09 (0.74, 1.62)	0.655	0.59 (0.30, 1.19)	0.139	0.95 (0.67, 1.37)	0.802	0.32 (0.04, 2.68)	0.295
Rich	0.87 (0.44, 1.75)	0.704	0.82 (0.37, 1.83)	0.628	1.09 (0.65, 1.82)	0.751	0.14 (0.02, 0.94)	0.043
***Household assets***								
***(ref: No)***								
Yes	0.91 (0.72, 1.15)	0.427	0.58 (0.42, 0.79)	0.001	0.77 (0.63, 0.94)	0.010	0.69 (0.39, 1.25)	0.222
***Currently pregnant***								
***(ref: No)***								
Yes	1.40 (0.96, 2.04)	0.084	1.10 (0.69, 1.73)	0.692	1.26 (0.90, 1.75)	0.174	1.62 (0.77, 3.41)	0.201
***Pregnancy history***								
***(ref: Single)***								
Multiple	0.95 (0.60, 1.52)	0.832	0.91 (0.57, 1.43)	0.667	0.92 (0.63, 1.34)	0.662	1.07 (0.54, 2.12)	0.843
***Pregnancy loss***								
***(ref: None)***								
One	1.01 (0.60, 1.70)	0.982	0.49 (0.29, 0.81)	0.006	0.70 (0.44, 1.11)	0.131	0.72 (0.36, 1.44)	0.357
Two or more	0.91 (0.40, 2.11)	0.831	1.55 (0.86, 2.78)	0.141	1.06 (0.57, 1.96)	0.850	1.87 (0.82, 4.28)	0.138
***IPV Justification***								
***(ref:No)***								
Yes	0.96 (0.66, 1.39)	0.810	1.03 (0.66, 1.61)	0.895	0.94 (0.68, 1.30)	0.707	1.86 (0.89, 3.90)	0.098
***Age at cohabitation***								
***(ref: ≤ 17)***								
Mild (18–20)	0.95 (0.69, 1.30)	0.747	1.03 (0.73, 1.44)	0.884	0.99 (0.77, 1.28)	0.946	1.27 (0.62, 2.62)	0.518
Older (21+)	0.91 (0.64, 1.31)	0.622	0.84 (0.64, 1.10)	0.203	0.83 (0.63, 1.10)	0.191	1.19 (0.65, 2.17)	0.573
***Water access***								
***(ref: Unimproved)***								
Improved	0.86 (0.59, 1.26)	0.437	0.82 (0.48, 1.42)	0.485	0.85 (0.60, 1.19)	0.338	0.95 (0.50, 1.81)	0.887
***Sanitation facilities***								
***(ref: Unimproved)***								
Improved	0.77 (0.54, 1.10)	0.148	0.73 (0.52, 1.03)	0.071	0.68 (0.51, 0.92)	0.011	1.25 (0.86, 1.82)	0.246
***Household materials***								
***(ref: Unimproved)***								
Improved	0.98 (0.72, 1.35)	0.917	2.43 (1.71, 3.45)	<0.001	1.29 (0.99, 1.70)	0.060	2.30 (0.80, 6.60)	0.121
***Media access***								
***(ref: No)***								
Yes	0.96 (0.71, 1.29)	0.785	1.04 (0.68, 1.61)	0.842	0.95 (0.75, 1.22)	0.707	—	—
***Internet use***								
***(ref: No)***								
Yes	2.06 (1.00, 4.22)	0.050	1.39 (0.99, 1.94)	0.054	—	—	—	—
***Province***								
***(ref: Maputo City)***								
Cabo Delgado	3.38 (1.66, 6.90)	0.001	3.06 (1.03, 9.13)	0.045	3.94 (2.08, 7.45)	<0.001	1.33 (0.27, 6.44)	0.724
Nampula	15.36 (9.02, 26.13)	<0.001	8.44 (4.48, 15.91)	<0.001	13.13 (8.43, 20.45)	<0.001	5.01 (1.55, 16.19)	0.007
Zambézia	3.85 (2.00, 7.42)	<0.001	1.44 (0.70, 2.96)	0.319	2.93 (1.64, 5.25)	<0.001	3.01 (0.95, 9.57)	0.062
Tete	2.08 (1.09, 3.96)	0.026	0.82 (0.33, 2.03)	0.668	1.44 (0.80, 2.60)	0.228	1.61 (0.45, 5.77)	0.467
Manica	0.66 (0.27, 1.59)	0.351	0.43 (0.21, 0.90)	0.026	0.64 (0.30, 1.37)	0.251	0.26 (0.06, 1.12)	0.071
Sofala	1.33 (0.66, 2.69)	0.417	2.54 (1.30, 4.94)	0.006	1.60 (0.90, 2.86)	0.112	2.59 (0.76, 8.89)	0.129
Inhambane	0.31 (0.09, 1.08)	0.065	0.44 (0.18, 1.09)	0.075	0.60 (0.25, 1.42)	0.245	0.13 (0.03, 0.53)	0.005
Gaza	0.25 (0.09, 0.68)	0.007	0.25 (0.08, 0.80)	0.019	0.28 (0.12, 0.66)	0.003	0.16 (0.04, 0.68)	0.013
Maputo Province	1.29 (0.46, 3.60)	0.622	2.53 (1.24, 5.15)	0.011	2.23 (1.06, 4.70)	0.035	1.77 (0.53, 5.91)	0.353
Cidade de Maputo	—	—	0.76 (0.41, 1.41)	0.381	0.85 (0.37, 1.95)	0.698	0.63 (0.18, 2.15)	0.457
***Religion***								
***(ref: Catholic)***								
Islam	1.07 (0.74, 1.54)	0.709	0.93 (0.56, 1.54)	0.770	1.06 (0.78, 1.44)	0.698	1.11 (0.58, 2.11)	0.755
Zion	1.42 (0.91, 2.21)	0.119	0.52 (0.31, 0.89)	0.017	1.17 (0.78, 1.75)	0.448	0.52 (0.25, 1.09)	0.083
Evangelical/Pentecostal	1.95 (1.21, 3.14)	0.006	0.53 (0.37, 0.77)	0.001	1.57 (1.04, 2.37)	0.031	0.54 (0.33, 0.88)	0.014
Other	1.61 (0.97, 2.66)	0.066	1.13 (0.68, 1.89)	0.639	1.68 (1.10, 2.57)	0.017	0.66 (0.30, 1.46)	0.309

Note. aOR = adjusted odds ratio; CI = confidence interval; Ref. = reference category. Models were estimated using survey-weighted (svy) logistic regression accounting for the complex survey design (strata, primary sampling units, and sampling weights). Reference categories are indicated in parentheses beneath each characteristic heading. Bold italicized headings denote categorical variable groupings; reference category odds = 1.00 (not shown). “—” indicates the variable was not included in or estimable for that model (e.g., omitted for collinearity or zero cell counts/subpopulation size). All reported p-values are two-tailed; statistical significance was set at p < 0.05.

### 3.6. Residence- and internet use–stratified analysis of factors associated with depressive symptoms

**Fig 4:**
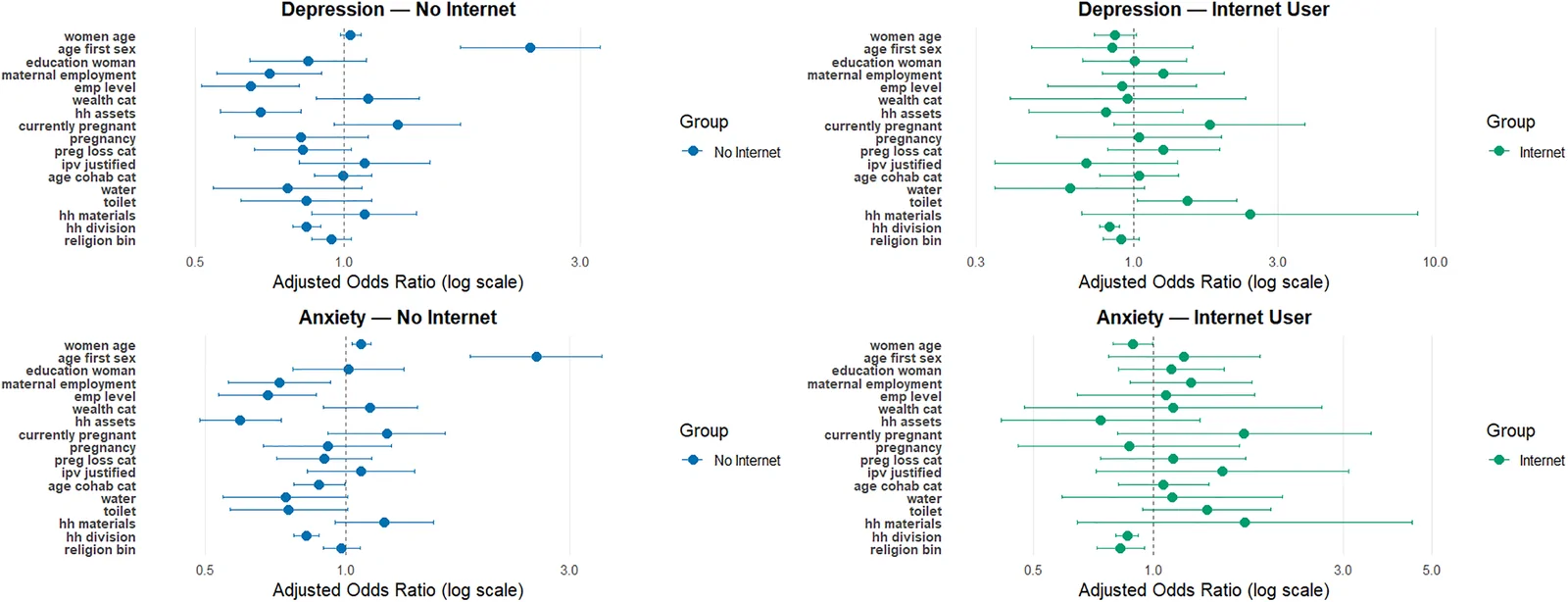
Survey-weighted associations of anxiety and depression with internet use.

Women aged 35–39 years had significantly higher odds of depression in rural (aOR = 1.56, 95% CI: 1.01–2.40, p = 0.043) and non-internet-user’s women (aOR = 1.52, 95% CI: 1.07–2.16, p = 0.019). Among internet users, higher odds were observed only in women aged 25–29 years (aOR = 2.37, 95% CI: 1.11–5.05, p = 0.026). Older age at first sexual intercourse (≥18 years) was associated with higher odds of depressive symptoms in rural (aOR = 1.96, 95% CI: 1.32–2.91, p = 0.001) and non-internet users (aOR = 1.82, 95% CI: 1.31–2.55, p < 0.001), indicating a consistent association across settings. Decision-making autonomy associated with significantly lower odds of depression in rural women (aOR = 0.60, 95% CI: 0.45–0.80, p = 0.001) compared to urban women and non-internet users (aOR = 0.64, 95% CI: 0.51–0.81, p < 0.001) compared to users. Pregnancy history was associated with higher odds of depression among rural women (aOR = 1.59, 95% CI: 1.11–2.29, p = 0.012). In contrast, women with two or more pregnancy losses associated with higher odds among internet users (aOR = 2.64, 95% CI: 1.16–5.97, p = 0.020). Household assets were associated with lower odds of depression in urban women (aOR = 0.71, 95% CI: 0.54–0.95, p = 0.023). Water access was associated with lower odds of depression among internet users (aOR = 0.55, 95% CI: 0.31–1.00, p = 0.049), while improved sanitation facilities associated with higher odds among internet users (aOR = 1.56, 95% CI: 1.05–2.31, p = 0.028). Compared to Niassa, higher odds of depression associated in the Nampula across rural (aOR = 14.59, 95% CI: 6.25–34.08, p < 0.001), urban (aOR = 5.60, 95% CI: 2.63–11.92, p < 0.001), and non-internet-user’s women (aOR = 9.96, 95% CI: 5.75–17.23, p < 0.001). Conversely, women in Gaza consistently showed lower odds of depressive symptoms across all models, including rural (aOR = 0.28, 95% CI: 0.08–0.95, p = 0.042) and internet users (aOR = 0.13, 95% CI: 0.03–0.54, p = 0.005). Manica and Inhambane also associated with lower odds of internet users (Manica: aOR = 0.06, 95% CI: 0.01–0.40, p = 0.004; Inhambane: aOR = 0.25, 95% CI: 0.07–0.98, p = 0.047) compared with Niassa (Fig 4, Table 5).

**Table 5:** Survey-weighted multivariable logistic regression analysis of factors associated with depressive symptoms among Mozambican women (N = 13,183).

Characteristic	Rural		Urban		No Internet Use		Internet Use	
	aOR (95% CI)	p	aOR (95% CI)	p	aOR (95% CI)	p	aOR (95% CI)	p
***Women’s age***								
***(ref: 15–19)***								
20–24	1.15 (0.78, 1.69)	0.483	1.36 (0.91, 2.05)	0.134	1.16 (0.84, 1.60)	0.356	1.67 (0.95, 2.94)	0.074
25–29	1.16 (0.77, 1.75)	0.477	1.26 (0.80, 1.99)	0.318	1.08 (0.78, 1.51)	0.633	2.37 (1.11, 5.05)	0.026
30–34	1.06 (0.66, 1.72)	0.801	1.20 (0.69, 2.09)	0.527	1.10 (0.75, 1.61)	0.624	1.42 (0.47, 4.27)	0.529
35–39	1.56 (1.01, 2.40)	0.043	1.38 (0.81, 2.34)	0.236	1.52 (1.07, 2.16)	0.019	0.79 (0.24, 2.55)	0.688
40–44	1.02 (0.64, 1.61)	0.944	0.98 (0.54, 1.77)	0.950	1.02 (0.70, 1.49)	0.912	0.57 (0.15, 2.13)	0.406
45–49	1.42 (0.91, 2.23)	0.126	1.03 (0.55, 1.92)	0.929	1.29 (0.88, 1.89)	0.187	0.53 (0.15, 1.91)	0.333
***Age at first sexual intercourse***								
***(ref: < 18 years)***								
≥ 18 years	1.96 (1.32, 2.91)	0.001	1.23 (0.76, 1.97)	0.396	1.82 (1.31, 2.55)	<0.001	0.71 (0.38, 1.34)	0.293
***Woman’s education***								
***(ref: None/Primary)***								
Secondary Incomplete	1.03 (0.61, 1.73)	0.924	0.78 (0.61, 1.02)	0.065	0.86 (0.63, 1.18)	0.346	0.66 (0.39, 1.11)	0.116
Secondary+	0.18 (0.02, 1.68)	0.131	0.87 (0.46, 1.66)	0.673	0.20 (0.02, 1.69)	0.138	0.71 (0.31, 1.63)	0.423
***Employment***								
***(ref: No)***								
Yes	0.86 (0.63, 1.18)	0.349	1.03 (0.73, 1.45)	0.885	0.92 (0.72, 1.19)	0.531	1.19 (0.75, 1.89)	0.458
***Decision Making Autonomy***								
***(ref: No)***								
Yes	0.60 (0.45, 0.80)	0.001	0.79 (0.58, 1.07)	0.123	0.64 (0.51, 0.81)	<0.001	0.83 (0.50, 1.35)	0.445
***Household wealth***								
***(ref: Poor)***								
Middle Class	0.87 (0.59, 1.30)	0.499	0.83 (0.41, 1.68)	0.599	0.90 (0.63, 1.29)	0.579	0.29 (0.04, 2.20)	0.232
Rich	1.03 (0.52, 2.04)	0.942	0.86 (0.40, 1.88)	0.709	1.14 (0.68, 1.92)	0.626	0.21 (0.03, 1.48)	0.116
***Household assets***								
***(ref: No)***								
Yes	0.94 (0.73, 1.21)	0.633	0.71 (0.54, 0.95)	0.023	0.84 (0.69, 1.04)	0.104	0.86 (0.47, 1.59)	0.630
***Currently pregnant***								
***(ref: No)***								
Yes	1.59 (1.11, 2.29)	0.012	0.92 (0.54, 1.59)	0.771	1.31 (0.95, 1.82)	0.100	1.63 (0.73, 3.64)	0.230
***Pregnancy history***								
***(ref: Single)***								
Multiple pregnancy	0.73 (0.47, 1.13)	0.159	1.09 (0.72, 1.66)	0.684	0.78 (0.55, 1.10)	0.154	1.41 (0.72, 2.76)	0.317
***Pregnancy loss***								
***(ref: None)***								
One loss	0.82 (0.52, 1.30)	0.392	0.72 (0.44, 1.15)	0.166	0.76 (0.52, 1.13)	0.177	0.71 (0.36, 1.42)	0.330
Two or more losses	0.46 (0.20, 1.04)	0.063	1.59 (0.79, 3.18)	0.192	0.66 (0.36, 1.22)	0.189	2.64 (1.16, 5.97)	0.020
***IPV Justification***								
***(ref: No)***								
Yes	0.93 (0.60, 1.45)	0.741	1.09 (0.75, 1.57)	0.658	0.98 (0.69, 1.39)	0.896	0.71 (0.34, 1.48)	0.363
***Age at cohabitation***								
***(ref: Young, < 18)***								
Mild (18–20)	1.01 (0.76, 1.33)	0.971	1.19 (0.84, 1.68)	0.332	1.06 (0.83, 1.35)	0.665	1.50 (0.70, 3.23)	0.298
Older (21+)	1.23 (0.86, 1.75)	0.252	0.93 (0.63, 1.36)	0.700	1.09 (0.82, 1.47)	0.545	1.19 (0.62, 2.29)	0.599
***Water Access***								
***(ref: Unimproved)***								
Improved	0.85 (0.54, 1.32)	0.467	0.80 (0.49, 1.31)	0.372	0.85 (0.57, 1.26)	0.411	0.55 (0.31, 1.00)	0.049
***Sanitation facility***								
***(ref: Unimproved)***								
Improved	0.93 (0.65, 1.31)	0.668	0.78 (0.54, 1.14)	0.203	0.77 (0.56, 1.05)	0.095	1.56 (1.05, 2.31)	0.028
***Household materials***								
***(ref: Unimproved)***								
Improved	1.17 (0.84, 1.61)	0.354	1.44 (0.99, 2.11)	0.059	1.17 (0.89, 1.54)	0.256	3.67 (0.81, 16.69)	0.092
***Media Access***								
***(ref: No)***								
Yes	0.81 (0.62, 1.06)	0.124	1.20 (0.79, 1.82)	0.388	0.87 (0.70, 1.09)	0.225	—	—
***Internet use***								
***(ref: No)***								
Yes	1.78 (0.85, 3.72)	0.126	1.09 (0.72, 1.67)	0.679	—	—	—	—
***Place of residence***								
***(ref: Urban)***								
Rural	—	—	—	—	0.86 (0.54, 1.35)	0.501	1.18 (0.60, 2.34)	0.633
***Province***								
***(ref: Niassa)***								
Cabo Delgado	8.03 (3.30, 19.55)	<0.001	3.02 (1.05, 8.75)	0.041	5.65 (3.03, 10.55)	<0.001	1.68 (0.32, 8.73)	0.536
Nampula	14.59 (6.25, 34.08)	<0.001	5.60 (2.63, 11.92)	<0.001	9.96 (5.75, 17.23)	<0.001	2.96 (0.81, 10.75)	0.099
Zambézia	5.09 (1.96, 13.22)	0.001	1.25 (0.42, 3.70)	0.687	3.24 (1.62, 6.48)	0.001	1.80 (0.39, 8.26)	0.447
Tete	1.53 (0.58, 4.02)	0.384	0.35 (0.09, 1.33)	0.124	1.07 (0.52, 2.17)	0.859	0.15 (0.02, 1.01)	0.051
Manica	0.84 (0.27, 2.64)	0.763	0.43 (0.15, 1.21)	0.109	0.79 (0.34, 1.83)	0.579	0.06 (0.01, 0.40)	0.004
Sofala	4.08 (1.64, 10.17)	0.003	2.57 (1.14, 5.77)	0.023	2.87 (1.52, 5.40)	0.001	2.10 (0.58, 7.60)	0.257
Inhambane	0.96 (0.34, 2.70)	0.939	0.84 (0.29, 2.38)	0.738	1.13 (0.50, 2.53)	0.774	0.25 (0.07, 0.98)	0.047
Gaza	0.28 (0.08, 0.95)	0.042	0.22 (0.07, 0.67)	0.009	0.20 (0.06, 0.69)	0.011	0.13 (0.03, 0.54)	0.005
Maputo Province	1.16 (0.37, 3.65)	0.796	1.38 (0.54, 3.53)	0.496	1.64 (0.65, 4.10)	0.292	0.57 (0.15, 2.15)	0.407
Cidade de Maputo	—	—	0.51 (0.23, 1.17)	0.111	0.89 (0.37, 2.14)	0.796	0.22 (0.06, 0.79)	0.020
***Religion***								
***(ref: Catholic)***								
Islam	0.86 (0.56, 1.33)	0.500	1.35 (0.79, 2.31)	0.271	0.99 (0.71, 1.38)	0.949	1.40 (0.65, 3.04)	0.388
Zion	1.21 (0.69, 2.10)	0.503	0.89 (0.52, 1.53)	0.679	0.96 (0.58, 1.57)	0.859	1.72 (0.81, 3.63)	0.157
Evangelical/Pentecostal	1.55 (0.96, 2.50)	0.071	0.82 (0.55, 1.21)	0.317	1.35 (0.91, 2.00)	0.142	0.94 (0.54, 1.64)	0.835
Other	1.17 (0.69, 2.00)	0.554	1.19 (0.68, 2.08)	0.541	1.18 (0.74, 1.88)	0.479	1.01 (0.46, 2.18)	0.988

Note. aOR = adjusted odds ratio; CI = confidence interval; Ref. = reference category. Models were estimated using survey-weighted (svy) logistic regression accounting for the complex survey design (strata, primary sampling units, and sampling weights). Reference categories are indicated in parentheses beneath each characteristic heading. Bold italicized headings denote categorical variable groupings; reference category odds = 1.00 (not shown). “—” indicates the variable was not included in or estimable for that model (e.g., omitted for collinearity or zero cell counts/subpopulation size). All reported p-values are two-tailed; statistical significance was set at p < 0.05.

### 3.7. Geographic distribution of anxiety and depressive symptoms across Mozambique

**Fig 5:**
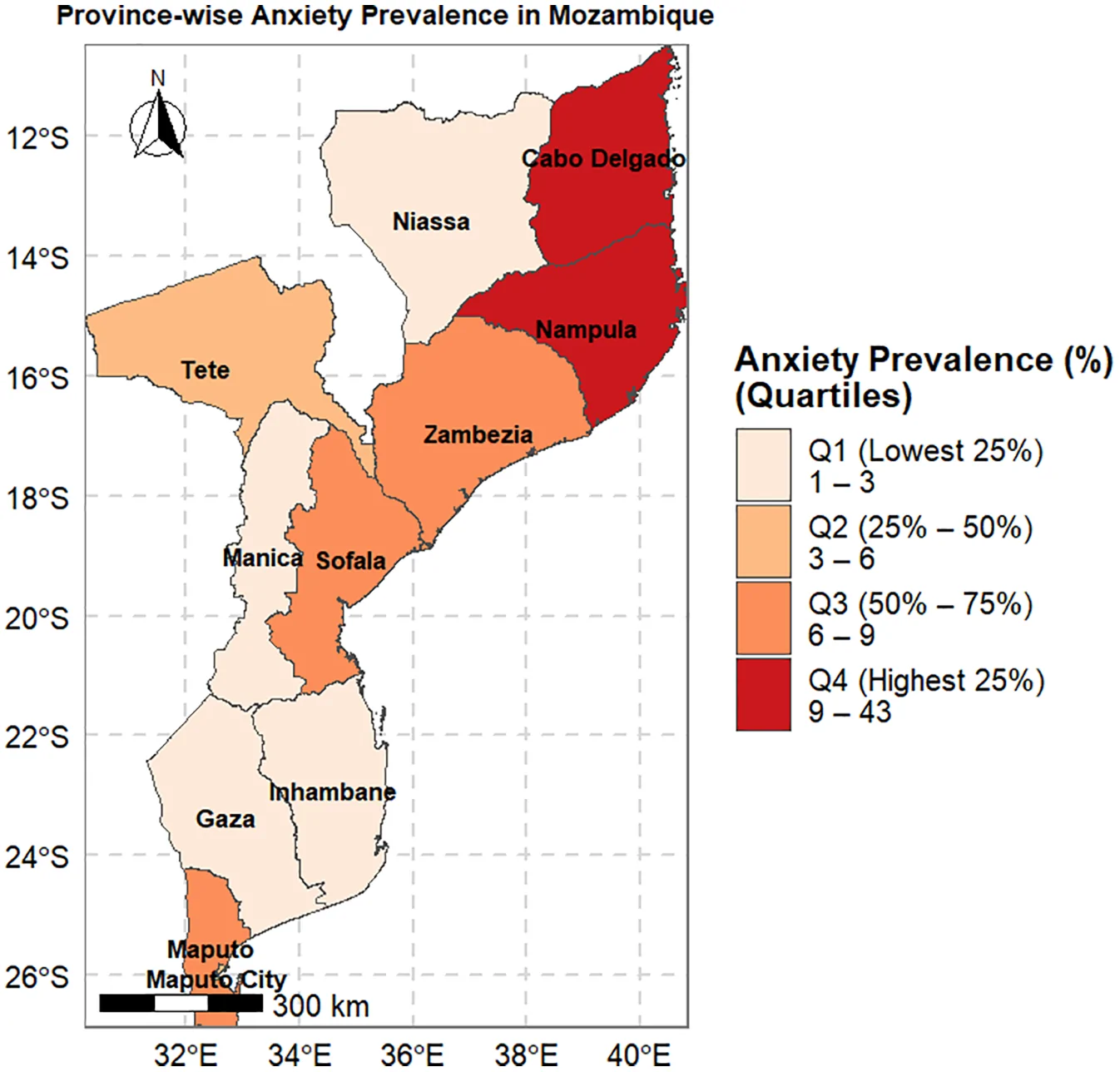
Geographical inequalities of anxiety prevalence across province. Provincial administrative boundary data were obtained from the Humanitarian Data Exchange (HDX) Common Operational Dataset (COD) for Mozambique (Source: Instituto Nacional de Estatística, Mozambique; OCHA Field Information Services Section). The map was created by the authors using R (packages: sf and ggplot2).

**Fig 6:**
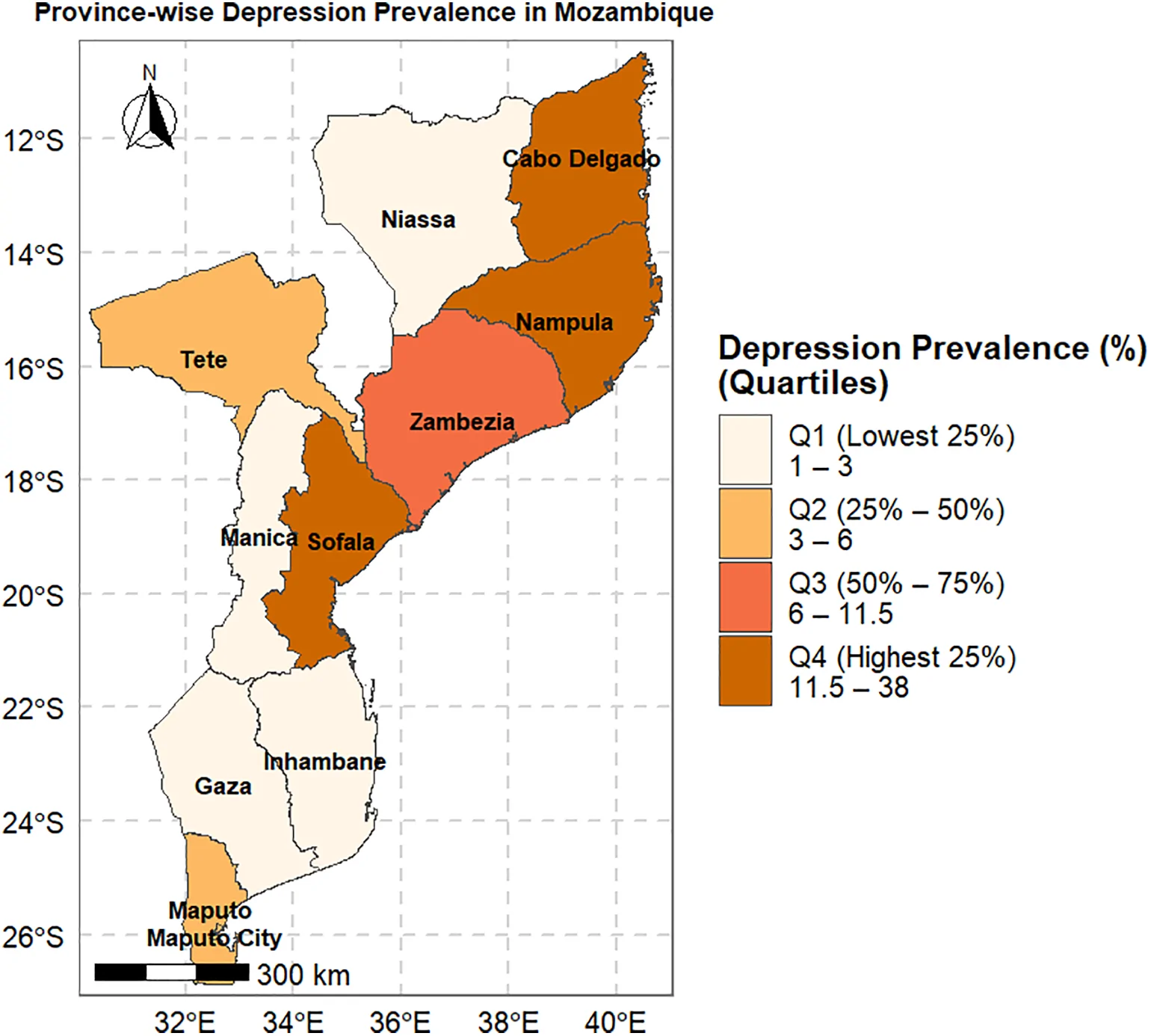
Geographical inequalities of depression prevalence across province. Provincial administrative boundary data were obtained from the Humanitarian Data Exchange (HDX) Common Operational Dataset (COD) for Mozambique (Source: Instituto Nacional de Estatística, Mozambique; OCHA Field Information Services Section). The map was created by the authors using R (packages: sf and ggplot2).

The highest prevalence of depression and anxiety were observed in the Nampula. Moderate depression rates are observed in the Sofala, Zambezia, and Cabo Delgado, while anxiety is notable in Cabo Delgado, Zambezia, and Sofala. The lowest prevalence for both outcomes observed in southern provinces, including Gaza Inhambane, and Niassa (Fig 5 and Fig 6).

### 3.8. Network analysis of factors associated with anxiety and depression

**Fig 7:**
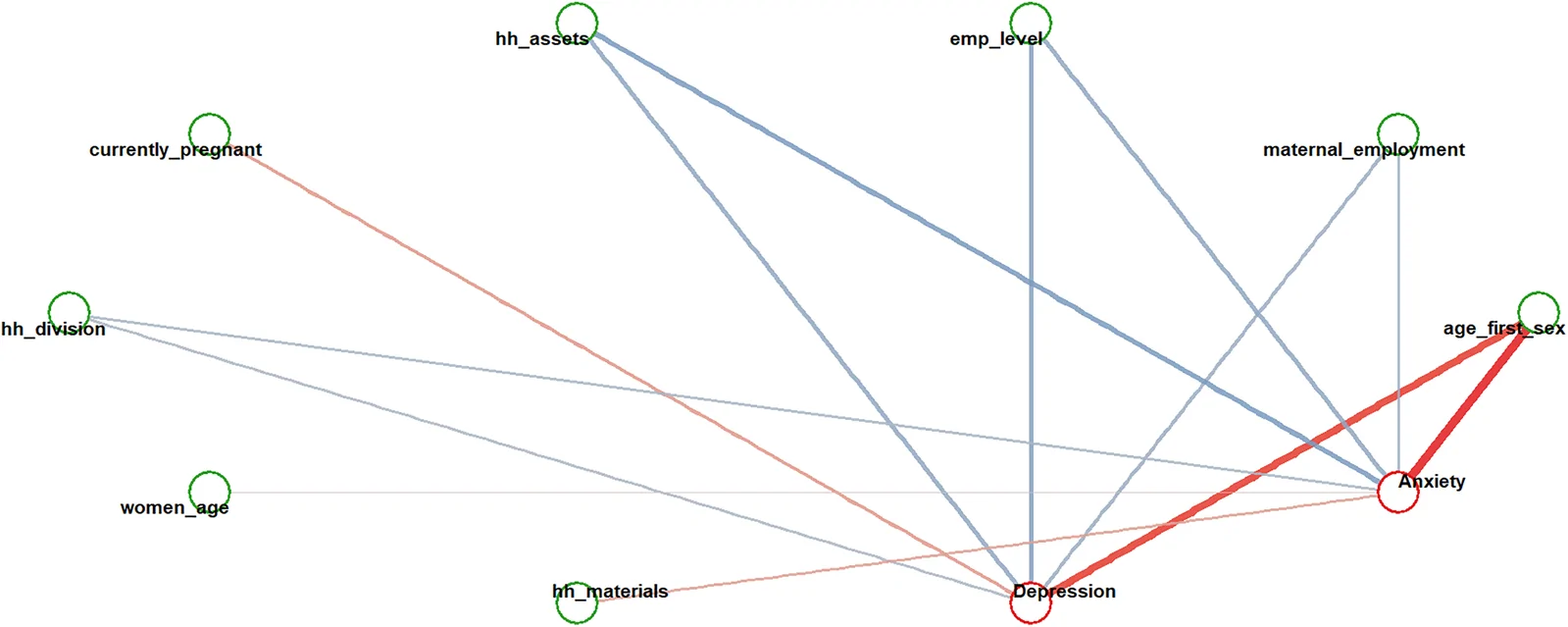
Network of significant risk factors for anxiety and depression.

The network analysis indicates a strong positive association between Depression and Anxiety. Age at first sex is the most important risk factor, followed by strong positive association with both outcomes. Other variables, such as household province, decision making autonomy, pregnancy status, household assets, employment, and women’s age, show weaker or modest associations with either Depression or Anxiety. Overall, the findings suggest that several common factors were associated with both anxiety and depressive symptoms, with earlier age at first sexual intercourse (<18 years) emerging as one of the notable associations with the adjusted analyses (Fig 7).

### 3.9. Discriminative performance of the multivariable models

**Fig 8:**
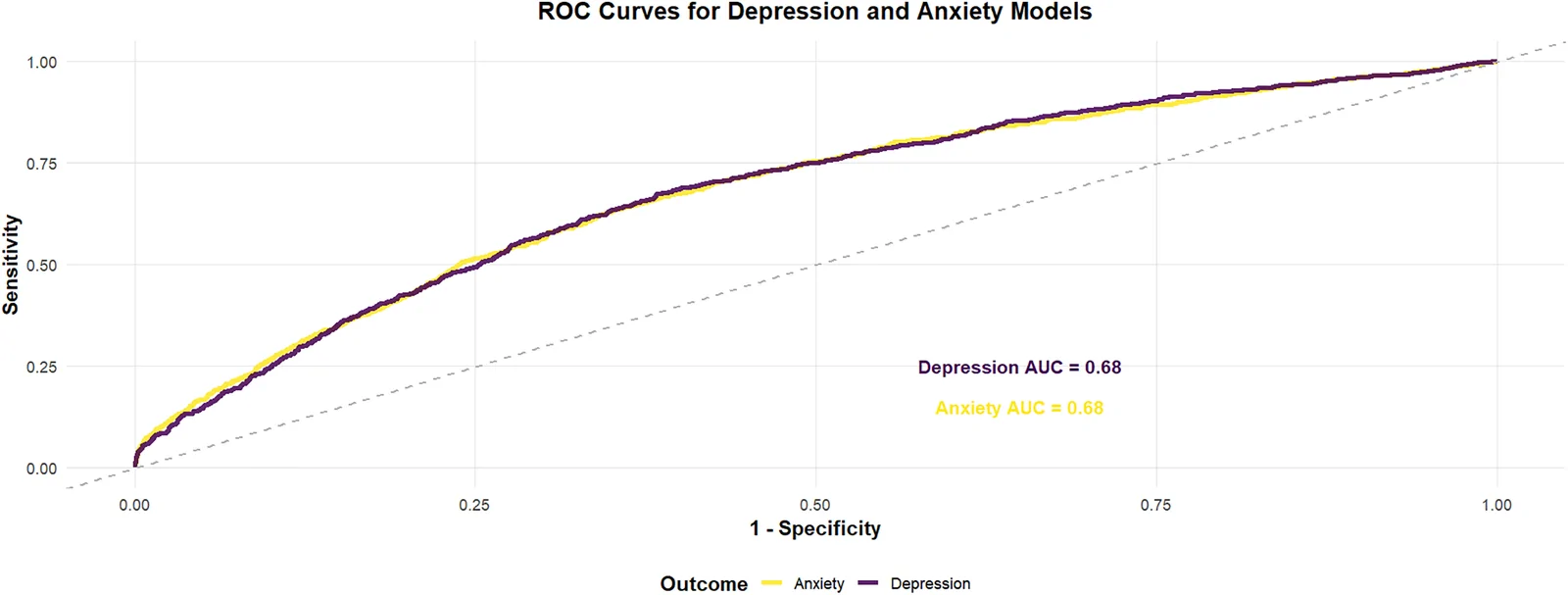
Receiver operating characteristic (ROC) curves of survey-weighted logistic regression models for anxiety and depression.

The ROC curves highlighted that both multivariable models had moderate discriminatory performance (AUC = 0.68 for both anxiety and depression), indicating a moderate ability to discriminate women with and without mental health symptoms (Fig 8).

## 4. Discussion

This study revealed substantial burden of mental health symptoms among Mozambican women, with approximately one in ten women reporting anxiety (11.1%) and depression (10.1%). The findings should be interpreted within the limitations of a cross-sectional design. Because exposure and outcome variables were evaluated simultaneously, temporal relationships cannot be established and causal inferences cannot be drawn. The key significant risk factors of both anxiety and depression are including age, age at first sex, decision-making autonomy and province. In summary, these findings indicate that mental health inequities of women in Mozambique are a complex result of reproductive, socioeconomic, geographical and digital determinants.

This higher odds of anxiety and depression in women between the ages of 35–39 may be associated with the accumulation of reproductive, care taking and economic responsibilities which tend to increase during middle age. While the crude prevalence was higher in younger women, the adjusted analyses indicated that the association with other factors may begin to exceed that of age-related social and economic pressures after this point. Women involved in household decision making autonomy revealed more control over health-seeking behaviors, their finances and reproductive choices, and therefore less vulnerable to psychological symptoms [14].

An interesting pattern was the association between the internet use and higher odds anxiety especially for women in rural areas. While the internet can provide access to information and social interactions, it can also present opportunities for misinformation, social comparison, cyber harassment and disturbing news content. Internet access in rural communities could raise awareness of socioeconomic inequities but not provides enough supports [13].

Therefore, the higher prevalence of rural internet users could be due to a combination of digital stressors and structural disadvantages that continue to exist. The rural–urban subgroup analyses also reveal the significant differences. The lower association of autonomy in decision-making autonomy and improve sanitation was more prevalent in the rural women’s who did not uses internet indicating a stronger role of basic social and environmental factors under limited resources [38].

On the other hand, the association between improve household materials and higher anxiety of urban women may be due to the residual socioeconomic complexities. Improved housing may not necessarily be associated with improved psychological well-being, and may also be associates with higher financial expectations, mental health symptoms in the urban, or optimum awareness of social inequalities [14]. Nampula is one of the poorest and limited developed provinces in Mozambique, with expectations of a high prevalence of early marriage and adolescent pregnancies, as well as low health access and high poverty rates. The structural conditions may associate with the mental health symptoms observed in the present study [18,19]. The insistently lower odds observed in Gaza, however, might be due to socioeconomic inequalities, health systems, social services, or cultural inequalities, and should be explored further. Targeted maternal mental health screening programmers that could be embedded in antenatal and reproductive health services could be beneficial for provinces like Nampula, Cabo Delgado, Sofala and Zambézia.

The association between socioeconomic resources and lower mental health symptoms, similarly, was consistent with the general literature on socioeconomic resources and mental health [18,20]. The significant association between internet use and anxiety, however, contrasts with the traditional benefits of the internet [13,39,40]. Recent findings from international studies are emerging that suggest a significant association between the internet and mental health, but that a lower odd can also be observed, depending on the quality, purpose and context of internet use [13,21,23]. The current results add to this expanding body of evidence to suggest that the digital exposure might constitute a specific vulnerability of rural Mozambican women. The high level of provincial differences highlighted here further confirms earlier Mozambican studies that have reported inequalities between regions with regard to maternal and reproductive health outcomes. The results indicate that mental health inequities could reflect national trends of social economic and health care inequities [18,31,41]. The present results contribute to this rising literature by demonstrating that digital exposure may represent a different vulnerability among rural Mozambican women [42–44].

The GAD-7 and PHQ-9 are reliable scales that have been widely employed in population-based surveys for the assessment of symptoms of anxiety and depression, respectively. The employment of such scales in the DHS framework provides an opportunity for standardized and consistent measurement of common mental disorder symptoms in the population. Nonetheless, they are not diagnostic but screening scales and do not measure some clinical disorders [24,41].

The results showed that a wide confidence interval is used to interpret. Relatively wide confidence intervals (e.g., for pregnancy loss, wealth status, provincial estimates etc.) were associated with several subgroup estimates, especially within the internet-user models [13,45–47]. Although early age at first birth, early sexual debut, and early cohabitation are consistent reproductive life-course events, each was examined distinctly to evaluate its independent association with mental health symptoms. Women whose first sexual intercourse occurred at age ≥ 18 years associated with higher odds of anxiety and depression compared with women whose first sexual intercourse occurred before age 18 years.

This study findings regarding pregnancy loss should be interpreted in light of previous evidence demonstrating that adverse reproductive experiences may be associated with the higher odds of poor mental health among women of reproductive age [13]. Although internet use was associated with higher odds of anxiety in our adjusted analyses, the cross-sectional design does not permit conclusions regarding causality. Internet use may act as a proxy for broader social, behavioral, or digital environmental factors that were not captured in the MDHS. Further longitudinal research is needed to clarify the mechanisms underlying this association. A wide confidence interval is associated with less statistical precision and could be due to a smaller number of individuals who belong to a specific subgroup, few observations within each of these subgroups, or to a high degree of variability among the observations [48,49].

### 4.1. Strengths and limitations

Its strengths encompass the application of a large, nationally representative sample; strong survey-weighted regression models; stratified analyses by residence and internet use; and geographic evaluation of provincial inequalities. A combination of these strategies offers a wholesome insight into how the mental health of women varies in terms of demographic, reproductive, socioeconomic, and geographic. Besides, based on theoretical, conceptual, and statistical framework, the forward stepwise model selection is another methodological strength.

However, this study is limited by the cross-sectional design that does not allow causal inference. The outcome of mental health symptoms was self-reported screening tools and not clinical diagnoses. Some province estimates were not consistently estimable across models due to sparse data and zero cell counts in certain subgroups, leading to exclusion because of collinearity or lack of variation. Reference categories for province also differed across models (Maputo City and Niassa), which may limit direct comparison of estimates across tables. Additionally, comorbid depression and anxiety were not evaluated as a combined outcome in the present study, which may limit a more inclusive understanding of overlapping mental health symptoms. Although internet use was associated with higher odds of anxiety, causal inference is not possible due to the cross-sectional design. Longitudinal studies are needed to establish causality and identify the adjusted or crude associations.

### 4.2. Implications on policies and practice

The findings suggest that mental health symptoms should be integrated into existing reproductive, antenatal, and community health services in Mozambique. This consistent association of decision-making autonomy suggests that interventions that enhance women’s role in household and reproductive decision-making autonomy could help to improve their mental health symptoms. Screening and counselling for mental health symptoms could be provided priority to women with reproductive problems, such as mental health symptoms during pregnancy and pregnancy loss. There is a particular need for targeted interventions in high burden provinces, which included women with significantly higher odds of anxiety and depression symptoms including Nampula, Cabo Delgado, Sofala and Zambézia. High burden province-specific mental health services programs integrated within maternal and primary healthcare systems may help reduce these inequalities. This association between internet use and anxiety symptoms, especially among the rural women, reveals the need to provide digital health policies that encourage safe and healthy use of the internet. Improving digital health literacy initiatives and ensuring access to trustworthy health information online can facilitate positive mental health outcomes for women using the internet, while reducing the risk of poor mental health outcomes. The results also highlight the need for more holistic social and environmental factors of mental health to be addressed. Improvements to basic service including sanitation, water access, household materials, and wealth status may improve mental health symptoms, particularly for socially vulnerable groups.

### 4.3. Conclusions

The results of this study showed that there is a significant level of anxiety and depression symptoms among the Mozambican women and that it is possible to identify important social, reproductive, environmental, digital, and geographical factors associated with these symptoms. Decision making autonomy was consistently associated with lower odds of anxiety and depression, while the internet use was associated with higher odds of anxiety symptoms only. Substantial provincial inequalities were observed, with Nampula consistently revealed the higher burden and Gaza showing comparatively lower odds of both outcomes. These findings revealed targeted, evidence-informed policies that address the social, reproductive, and geographic determinants of women’s mental health symptoms in Mozambique. Routine integration of mental health indicators screening into reproductive health and women’s health services, together with strengthening women’s decision-making autonomy, promoting digital literacy and prioritizing high-burden provinces, may help decrease mental health indicators disparities among Mozambican women. Overall, our findings also support integrating maternal mental health services with interventions that promote women’s decision-making autonomy, strengthen gender equity, reduce the acceptance and occurrence of intimate partner violence, and expand access to appropriate mental support, particularly in high-burden provinces. Despite the lack of causal inferences with respect to the cross-sectional design, the findings are relevant evidence for guiding focused mental health policies and moving towards better maternal health and Sustainable Development Goal 3 (SDG 3). Women’s health policies should integrate mental health symptoms promotion, screening, and referral services across the reproductive life course, particularly for women at higher risk of anxiety and depression symptoms.

## Supporting information

S1 Table Frequency distributions for GAD-7 and PHQ-9 items (n = 13,183). (DOCX)

S1 Fig Study area of Mozambique. (TIFF)
