# Correction: Stratified and geographic patterns of depression and anxiety in Mozambican women: Mental health policy implications

**DOI:** 10.1371/journal.pone.0362565

**Published:** 2026-11-10

**Authors:** Md Salek Miah

Tables 3–5 were uploaded incorrectly. Please see the correct Tables 3–5 here.

**Table 3:** Survey-weighted multivariable logistic regression analysis of factors associated with anxiety and depressive symptoms among women (N= 13,183).

Variables	Anxiety symptoms (AOR [95% CI])	p-value	Depressive symptoms (AOR [95% CI])	p-value
**Women Age**				
15–19	Ref	–	Ref	–
20–24	1.28 [0.97, 1.69]	0.075	1.24 [0.94, 1.65]	0.134
25–29	1.43 [1.03, 1.97]	0.031	1.23 [0.90, 1.67]	0.192
30–34	1.41 [0.98, 2.02]	0.061	1.17 [0.82, 1.66]	0.396
35–39	2.06 [1.48, 2.87]	**<0.001**	1.49 [1.07, 2.08]	0.020
40–44	1.23 [0.81, 1.85]	0.328	1.03 [0.72, 1.47]	0.882
45–49	1.58 [1.12, 2.23]	0.009	1.32 [0.92, 1.89]	0.132
**Age at first sex**				
<18 years	Ref	–	Ref	–
≥18 years	1.66 [1.25, 2.19]	**<0.001**	1.60 [1.17, 2.18]	0.003
**Women Education**				
Below Primary	Ref	–	Ref	–
Secondary Incomplete	1.05 [0.82, 1.35]	0.669	0.85 [0.65, 1.12]	0.254
Secondary+	0.82 [0.50, 1.35]	0.436	0.74 [0.40, 1.39]	0.353
**Employment**				
No	Ref	–	Ref	–
Yes	1.01 [0.81, 1.26]	0.922	0.93 [0.74, 1.16]	0.519
**Decision making Autonomy**				
No	Ref	–	Ref	–
Yes	0.67 [0.54, 0.83]	**<0.001**	0.65 [0.53, 0.81]	**<0.001**
**Wealth**				
Poor	Ref	–	Ref	–
Middle Class	0.92 [0.65, 1.31]	0.650	0.88 [0.62, 1.24]	0.456
Rich	1.00 [0.61, 1.65]	0.986	1.08 [0.66, 1.75]	0.769
**Household assets**				
No	Ref	–	Ref	–
Yes	0.76 [0.62, 0.92]	0.005	0.84 [0.69, 1.02]	0.077
**Currently pregnant**				
No	Ref	–	Ref	–
Yes	1.29 [0.95, 1.76]	0.100	1.35 [1.00, 1.83]	0.052
**Pregnancy History**				
Single	Ref	–	Ref	–
Multiple pregnancy	0.95 [0.67, 1.32]	0.745	0.85 [0.62, 1.16]	0.294
**Pregnancy loss**				
None	Ref	–	Ref	–
One loss	0.71 [0.49, 1.04]	0.075	0.74 [0.53, 1.03]	0.076
Two or more losses	1.18 [0.72, 1.92]	0.512	0.87 [0.52, 1.46]	0.604
**IPV justification**				
No	Ref	–	Ref	–
Yes	0.98 [0.72, 1.33]	0.904	0.96 [0.69, 1.33]	0.794
**Age at cohabitation**				
≤17 years	Ref	–	Ref	–
Mild (18–20)	1.04 [0.83, 1.31]	0.708	1.12 [0.90, 1.40]	0.300
Older (21+)	0.87 [0.68, 1.12]	0.271	1.11 [0.85, 1.45]	0.439
**Water Access**				
Unimproved	Ref	–	Ref	–
Improved	0.86 [0.63, 1.18]	0.356	0.84 [0.58, 1.21]	0.343
**Sanitation Facilities**				
Unimproved	Ref	–	Ref	–
Improved	0.76 [0.60, 0.98]	0.035	0.85 [0.65, 1.11]	0.231
**Household materials**				
Unimproved	Ref	–	Ref	–
Improved	1.37 [1.07, 1.76]	0.013	1.25 [0.96, 1.62]	0.096
**Media Access**				
No	Ref	–	Ref	–
Yes	0.97 [0.76, 1.23]	0.793	0.88 [0.71, 1.10]	0.263
**Internet use**				
No	Ref	–	Ref	–
Yes	1.46 [1.07, 1.99]	0.018	1.16 [0.80, 1.68]	0.421
**Province**				
Maputo Cidade	Ref	–	Ref	–
Cabo Delgado	3.47 [1.83, 6.57]	**<0.001**	4.99 [2.67, 9.33]	**<0.001**
Nampula	12.06 [8.05, 18.06]	**<0.001**	9.02 [5.34, 15.23]	**<0.001**
Zambézia	3.03 [1.82, 5.02]	<0.001	3.06 [1.59, 5.86]	0.001
Tete	1.57 [0.95, 2.60]	0.080	0.90 [0.46, 1.78]	0.765
Manica	0.59 [0.31, 1.12]	0.107	0.60 [0.28, 1.32]	0.204
Sofala	1.96 [1.17, 3.28]	0.011	3.04 [1.68, 5.50]	**<0.001**
Inhambane	0.37 [0.17, 0.80]	0.012	0.90 [0.45, 1.81]	0.762
Gaza	0.23 [0.11, 0.49]	**<0.001**	0.23 [0.10, 0.53]	0.001
Maputo	1.87 [0.98, 3.56]	0.059	1.37 [0.63, 2.96]	0.427
**Religion**				
Catholic	Ref	–	Ref	–
Islam	1.05 [0.79, 1.40]	0.737	1.02 [0.74, 1.41]	0.897
Zion	1.06 [0.76, 1.49]	0.727	1.10 [0.74, 1.65]	0.641
Evangelical/Pentecostal	1.28 [0.91, 1.79]	0.158	1.25 [0.89, 1.76]	0.198
Other	1.48 [1.02, 2.16]	0.039	1.18 [0.78, 1.78]	0.432
**Place of Residence**				
Urban	Ref	–	Ref	–
Rural	0.93 [0.64, 1.34]	0.679	0.89 [0.60, 1.33]	0.583

**Table 4:** Survey-weighted multivariable logistic regression of factors associated with anxiety symptoms among women in Mozambique, stratified by place of residence and internet use (N = 13,183).

Characteristic	Rural		Urban		No Internet		Internet	
	aOR (95% CI)	p	aOR (95% CI)	p	aOR (95% CI)	p	aOR (95% CI)	p
***Women's age***								
***(ref: 15–19)***								
20–24	1.22 (0.84, 1.76)	0.296	1.38 (0.91, 2.10)	0.133	1.30 (0.96, 1.76)	0.088	1.25 (0.67, 2.33)	0.475
25–29	1.23 (0.81, 1.87)	0.339	1.72 (0.99, 2.99)	0.053	1.32 (0.93, 1.85)	0.116	2.21 (1.05, 4.63)	0.036
30–34	1.38 (0.87, 2.19)	0.169	1.32 (0.71, 2.44)	0.378	1.41 (0.95, 2.08)	0.086	1.30 (0.53, 3.17)	0.566
35–39	1.88 (1.20, 2.94)	0.006	2.25 (1.36, 3.72)	0.002	2.21 (1.54, 3.18)	<0.001	1.11 (0.45, 2.74)	0.823
40–44	1.37 (0.79, 2.35)	0.260	0.90 (0.50, 1.61)	0.713	1.29 (0.83, 2.02)	0.259	0.62 (0.23, 1.70)	0.355
45–49	1.86 (1.22, 2.82)	0.004	1.00 (0.52, 1.93)	0.992	1.68 (1.18, 2.39)	0.004	0.54 (0.17, 1.75)	0.307
***Age at first sex***								
***(ref: <18 years)***								
≥18 years	1.88 (1.25, 2.83)	0.002	1.44 (1.01, 2.04)	0.043	1.82 (1.32, 2.51)	<0.001	1.05 (0.65, 1.70)	0.826
***Women's education***								
***(ref: Below primary)***								
Secondary incomplete	1.36 (0.88, 2.12)	0.168	0.89 (0.68, 1.16)	0.390	1.03 (0.77, 1.38)	0.838	0.91 (0.52, 1.59)	0.733
Secondary+	0.78 (0.21, 2.91)	0.711	0.72 (0.42, 1.24)	0.241	0.43 (0.07, 2.54)	0.350	0.70 (0.35, 1.42)	0.324
***Employment***								
***(ref: No)***								
Yes	0.89 (0.63, 1.26)	0.509	1.14 (0.85, 1.53)	0.364	1.01 (0.78, 1.31)	0.942	1.24 (0.85, 1.81)	0.259
***Decision-making autonomy***								
***(ref: No)***								
Yes	0.59 (0.45, 0.79)	<0.001	0.80 (0.56, 1.14)	0.219	0.64 (0.51, 0.82)	<0.001	0.93 (0.59, 1.47)	0.751
***Household wealth***								
***(ref: Poor)***								
Middle	1.09 (0.74, 1.62)	0.655	0.59 (0.30, 1.19)	0.139	0.95 (0.67, 1.37)	0.802	0.32 (0.04, 2.68)	0.295
Rich	0.87 (0.44, 1.75)	0.704	0.82 (0.37, 1.83)	0.628	1.09 (0.65, 1.82)	0.751	0.14 (0.02, 0.94)	0.043
***Household assets***								
***(ref: No)***								
Yes	0.91 (0.72, 1.15)	0.427	0.58 (0.42, 0.79)	0.001	0.77 (0.63, 0.94)	0.010	0.69 (0.39, 1.25)	0.222
***Currently pregnant***								
***(ref: No)***								
Yes	1.40 (0.96, 2.04)	0.084	1.10 (0.69, 1.73)	0.692	1.26 (0.90, 1.75)	0.174	1.62 (0.77, 3.41)	0.201
***Pregnancy history***								
***(ref: Single)***								
Multiple	0.95 (0.60, 1.52)	0.832	0.91 (0.57, 1.43)	0.667	0.92 (0.63, 1.34)	0.662	1.07 (0.54, 2.12)	0.843
***Pregnancy loss***								
***(ref: None)***								
One	1.01 (0.60, 1.70)	0.982	0.49 (0.29, 0.81)	0.006	0.70 (0.44, 1.11)	0.131	0.72 (0.36, 1.44)	0.357
Two or more	0.91 (0.40, 2.11)	0.831	1.55 (0.86, 2.78)	0.141	1.06 (0.57, 1.96)	0.850	1.87 (0.82, 4.28)	0.138
***IPV Justification***								
***(ref:No)***								
Yes	0.96 (0.66, 1.39)	0.810	1.03 (0.66, 1.61)	0.895	0.94 (0.68, 1.30)	0.707	1.86 (0.89, 3.90)	0.098
***Age at cohabitation***								
***(ref: ≤17)***								
Mild (18–20)	0.95 (0.69, 1.30)	0.747	1.03 (0.73, 1.44)	0.884	0.99 (0.77, 1.28)	0.946	1.27 (0.62, 2.62)	0.518
Older (21+)	0.91 (0.64, 1.31)	0.622	0.84 (0.64, 1.10)	0.203	0.83 (0.63, 1.10)	0.191	1.19 (0.65, 2.17)	0.573
***Water access***								
***(ref: Unimproved)***								
Improved	0.86 (0.59, 1.26)	0.437	0.82 (0.48, 1.42)	0.485	0.85 (0.60, 1.19)	0.338	0.95 (0.50, 1.81)	0.887
***Sanitation facilities***								
***(ref: Unimproved)***								
Improved	0.77 (0.54, 1.10)	0.148	0.73 (0.52, 1.03)	0.071	0.68 (0.51, 0.92)	0.011	1.25 (0.86, 1.82)	0.246
***Household materials***								
***(ref: Unimproved)***								
Improved	0.98 (0.72, 1.35)	0.917	2.43 (1.71, 3.45)	<0.001	1.29 (0.99, 1.70)	0.060	2.30 (0.80, 6.60)	0.121
***Media access***								
***(ref: No)***								
Yes	0.96 (0.71, 1.29)	0.785	1.04 (0.68, 1.61)	0.842	0.95 (0.75, 1.22)	0.707	—	—
***Internet use***								
***(ref: No)***								
Yes	2.06 (1.00, 4.22)	0.050	1.39 (0.99, 1.94)	0.054	—	—	—	—
***Province***								
***(ref: Maputo City)***								
Cabo Delgado	3.38 (1.66, 6.90)	0.001	3.06 (1.03, 9.13)	0.045	3.94 (2.08, 7.45)	<0.001	1.33 (0.27, 6.44)	0.724
Nampula	15.36 (9.02, 26.13)	<0.001	8.44 (4.48, 15.91)	<0.001	13.13 (8.43, 20.45)	<0.001	5.01 (1.55, 16.19)	0.007
Zambézia	3.85 (2.00, 7.42)	<0.001	1.44 (0.70, 2.96)	0.319	2.93 (1.64, 5.25)	<0.001	3.01 (0.95, 9.57)	0.062
Tete	2.08 (1.09, 3.96)	0.026	0.82 (0.33, 2.03)	0.668	1.44 (0.80, 2.60)	0.228	1.61 (0.45, 5.77)	0.467
Manica	0.66 (0.27, 1.59)	0.351	0.43 (0.21, 0.90)	0.026	0.64 (0.30, 1.37)	0.251	0.26 (0.06, 1.12)	0.071
Sofala	1.33 (0.66, 2.69)	0.417	2.54 (1.30, 4.94)	0.006	1.60 (0.90, 2.86)	0.112	2.59 (0.76, 8.89)	0.129
Inhambane	0.31 (0.09, 1.08)	0.065	0.44 (0.18, 1.09)	0.075	0.60 (0.25, 1.42)	0.245	0.13 (0.03, 0.53)	0.005
Gaza	0.25 (0.09, 0.68)	0.007	0.25 (0.08, 0.80)	0.019	0.28 (0.12, 0.66)	0.003	0.16 (0.04, 0.68)	0.013
Maputo Province	1.29 (0.46, 3.60)	0.622	2.53 (1.24, 5.15)	0.011	2.23 (1.06, 4.70)	0.035	1.77 (0.53, 5.91)	0.353
Cidade de Maputo	—	—	0.76 (0.41, 1.41)	0.381	0.85 (0.37, 1.95)	0.698	0.63 (0.18, 2.15)	0.457
***Religion***								
***(ref: Catholic)***								
Islam	1.07 (0.74, 1.54)	0.709	0.93 (0.56, 1.54)	0.770	1.06 (0.78, 1.44)	0.698	1.11 (0.58, 2.11)	0.755
Zion	1.42 (0.91, 2.21)	0.119	0.52 (0.31, 0.89)	0.017	1.17 (0.78, 1.75)	0.448	0.52 (0.25, 1.09)	0.083
Evangelical/Pentecostal	1.95 (1.21, 3.14)	0.006	0.53 (0.37, 0.77)	0.001	1.57 (1.04, 2.37)	0.031	0.54 (0.33, 0.88)	0.014
Other	1.61 (0.97, 2.66)	0.066	1.13 (0.68, 1.89)	0.639	1.68 (1.10, 2.57)	0.017	0.66 (0.30, 1.46)	0.309

Note. aOR = adjusted odds ratio; CI = confidence interval; Ref. = reference category. Models were estimated using survey-weighted (svy) logistic regression accounting for the complex survey design (strata, primary sampling units, and sampling weights). Reference categories are indicated in parentheses beneath each characteristic heading. Bold italicized headings denote categorical variable groupings; reference category odds = 1.00 (not shown). “—” indicates the variable was not included in or estimable for that model (e.g., omitted for collinearity or zero cell counts/subpopulation size). All reported p-values are two-tailed; statistical significance was set at p < 0.05.

**Table 5:** Survey-weighted multivariable logistic regression of factors associated with depressive symptoms among women in Mozambique, stratified by place of residence and internet use (N = 13,183).

Characteristic	Rural		Urban		No Internet Use		Internet Use	
	aOR (95% CI)	p	aOR (95% CI)	p	aOR (95% CI)	p	aOR (95% CI)	p
***Women's age***								
***(ref: 15–19)***								
20–24	1.15 (0.78, 1.69)	0.483	1.36 (0.91, 2.05)	0.134	1.16 (0.84, 1.60)	0.356	1.67 (0.95, 2.94)	0.074
25–29	1.16 (0.77, 1.75)	0.477	1.26 (0.80, 1.99)	0.318	1.08 (0.78, 1.51)	0.633	2.37 (1.11, 5.05)	0.026
30–34	1.06 (0.66, 1.72)	0.801	1.20 (0.69, 2.09)	0.527	1.10 (0.75, 1.61)	0.624	1.42 (0.47, 4.27)	0.529
35–39	1.56 (1.01, 2.40)	0.043	1.38 (0.81, 2.34)	0.236	1.52 (1.07, 2.16)	0.019	0.79 (0.24, 2.55)	0.688
40–44	1.02 (0.64, 1.61)	0.944	0.98 (0.54, 1.77)	0.950	1.02 (0.70, 1.49)	0.912	0.57 (0.15, 2.13)	0.406
45–49	1.42 (0.91, 2.23)	0.126	1.03 (0.55, 1.92)	0.929	1.29 (0.88, 1.89)	0.187	0.53 (0.15, 1.91)	0.333
***Age at first sexual intercourse***								
***(ref: < 18 years)***								
≥18 years	1.96 (1.32, 2.91)	0.001	1.23 (0.76, 1.97)	0.396	1.82 (1.31, 2.55)	**<0.001**	0.71 (0.38, 1.34)	0.293
***Woman's education***								
***(ref: None/Primary)***								
Secondary Incomplete	1.03 (0.61, 1.73)	0.924	0.78 (0.61, 1.02)	0.065	0.86 (0.63, 1.18)	0.346	0.66 (0.39, 1.11)	0.116
Secondary+	0.18 (0.02, 1.68)	0.131	0.87 (0.46, 1.66)	0.673	0.20 (0.02, 1.69)	0.138	0.71 (0.31, 1.63)	0.423
***Employment***								
***(ref: No)***								
Yes	0.86 (0.63, 1.18)	0.349	1.03 (0.73, 1.45)	0.885	0.92 (0.72, 1.19)	0.531	1.19 (0.75, 1.89)	0.458
***Decision Making Autonomy***								
***(ref: No)***								
Yes	0.60 (0.45, 0.80)	0.001	0.79 (0.58, 1.07)	0.123	0.64 (0.51, 0.81)	**<0.001**	0.83 (0.50, 1.35)	0.445
***Household wealth***								
***(ref: Poor)***								
Middle Class	0.87 (0.59, 1.30)	0.499	0.83 (0.41, 1.68)	0.599	0.90 (0.63, 1.29)	0.579	0.29 (0.04, 2.20)	0.232
Rich	1.03 (0.52, 2.04)	0.942	0.86 (0.40, 1.88)	0.709	1.14 (0.68, 1.92)	0.626	0.21 (0.03, 1.48)	0.116
***Household assets***								
***(ref: No)***								
Yes	0.94 (0.73, 1.21)	0.633	0.71 (0.54, 0.95)	0.023	0.84 (0.69, 1.04)	0.104	0.86 (0.47, 1.59)	0.630
***Currently pregnant***								
***(ref: No)***								
Yes	1.59 (1.11, 2.29)	0.012	0.92 (0.54, 1.59)	0.771	1.31 (0.95, 1.82)	0.100	1.63 (0.73, 3.64)	0.230
***Pregnancy history***								
***(ref: Single)***								
Multiple pregnancy	0.73 (0.47, 1.13)	0.159	1.09 (0.72, 1.66)	0.684	0.78 (0.55, 1.10)	0.154	1.41 (0.72, 2.76)	0.317
***Pregnancy loss***								
***(ref: None)***								
One loss	0.82 (0.52, 1.30)	0.392	0.72 (0.44, 1.15)	0.166	0.76 (0.52, 1.13)	0.177	0.71 (0.36, 1.42)	0.330
Two or more losses	0.46 (0.20, 1.04)	0.063	1.59 (0.79, 3.18)	0.192	0.66 (0.36, 1.22)	0.189	2.64 (1.16, 5.97)	0.020
***IPV Justification***								
***(ref: No)***								
Yes	0.93 (0.60, 1.45)	0.741	1.09 (0.75, 1.57)	0.658	0.98 (0.69, 1.39)	0.896	0.71 (0.34, 1.48)	0.363
***Age at cohabitation***								
***(ref: Young, < 18)***								
Mild (18–20)	1.01 (0.76, 1.33)	0.971	1.19 (0.84, 1.68)	0.332	1.06 (0.83, 1.35)	0.665	1.50 (0.70, 3.23)	0.298
Older (21+)	1.23 (0.86, 1.75)	0.252	0.93 (0.63, 1.36)	0.700	1.09 (0.82, 1.47)	0.545	1.19 (0.62, 2.29)	0.599
***Water Access***								
***(ref: Unimproved)***								
Improved	0.85 (0.54, 1.32)	0.467	0.80 (0.49, 1.31)	0.372	0.85 (0.57, 1.26)	0.411	0.55 (0.31, 1.00)	0.049
***Sanitation facility***								
***(ref: Unimproved)***								
Improved	0.93 (0.65, 1.31)	0.668	0.78 (0.54, 1.14)	0.203	0.77 (0.56, 1.05)	0.095	1.56 (1.05, 2.31)	0.028
***Household materials***								
***(ref: Unimproved)***								
Improved	1.17 (0.84, 1.61)	0.354	1.44 (0.99, 2.11)	0.059	1.17 (0.89, 1.54)	0.256	3.67 (0.81, 16.69)	0.092
***Media Access***								
***(ref: No)***								
Yes	0.81 (0.62, 1.06)	0.124	1.20 (0.79, 1.82)	0.388	0.87 (0.70, 1.09)	0.225	—	—
***Internet use***								
***(ref: No)***								
Yes	1.78 (0.85, 3.72)	0.126	1.09 (0.72, 1.67)	0.679	—	—	—	—
***Place of residence***								
***(ref: Urban)***								
Rural	—	—	—	—	0.86 (0.54, 1.35)	0.501	1.18 (0.60, 2.34)	0.633
***Province***								
***(ref: Niassa)***								
Cabo Delgado	8.03 (3.30, 19.55)	**<0.001**	3.02 (1.05, 8.75)	0.041	5.65 (3.03, 10.55)	**<0.001**	1.68 (0.32, 8.73)	0.536
Nampula	14.59 (6.25, 34.08)	**<0.001**	5.60 (2.63, 11.92)	**<0.001**	9.96 (5.75, 17.23)	**<0.001**	2.96 (0.81, 10.75)	0.099
Zambézia	5.09 (1.96, 13.22)	0.001	1.25 (0.42, 3.70)	0.687	3.24 (1.62, 6.48)	0.001	1.80 (0.39, 8.26)	0.447
Tete	1.53 (0.58, 4.02)	0.384	0.35 (0.09, 1.33)	0.124	1.07 (0.52, 2.17)	0.859	0.15 (0.02, 1.01)	0.051
Manica	0.84 (0.27, 2.64)	0.763	0.43 (0.15, 1.21)	0.109	0.79 (0.34, 1.83)	0.579	0.06 (0.01, 0.40)	0.004
Sofala	4.08 (1.64, 10.17)	0.003	2.57 (1.14, 5.77)	0.023	2.87 (1.52, 5.40)	0.001	2.10 (0.58, 7.60)	0.257
Inhambane	0.96 (0.34, 2.70)	0.939	0.84 (0.29, 2.38)	0.738	1.13 (0.50, 2.53)	0.774	0.25 (0.07, 0.98)	0.047
Gaza	0.28 (0.08, 0.95)	0.042	0.22 (0.07, 0.67)	0.009	0.20 (0.06, 0.69)	0.011	0.13 (0.03, 0.54)	0.005
Maputo Province	1.16 (0.37, 3.65)	0.796	1.38 (0.54, 3.53)	0.496	1.64 (0.65, 4.10)	0.292	0.57 (0.15, 2.15)	0.407
Cidade de Maputo	—	—	0.51 (0.23, 1.17)	0.111	0.89 (0.37, 2.14)	0.796	0.22 (0.06, 0.79)	0.020
***Religion***								
***(ref: Catholic)***								
Islam	0.86 (0.56, 1.33)	0.500	1.35 (0.79, 2.31)	0.271	0.99 (0.71, 1.38)	0.949	1.40 (0.65, 3.04)	0.388
Zion	1.21 (0.69, 2.10)	0.503	0.89 (0.52, 1.53)	0.679	0.96 (0.58, 1.57)	0.859	1.72 (0.81, 3.63)	0.157
Evangelical/Pentecostal	1.55 (0.96, 2.50)	0.071	0.82 (0.55, 1.21)	0.317	1.35 (0.91, 2.00)	0.142	0.94 (0.54, 1.64)	0.835
Other	1.17 (0.69, 2.00)	0.554	1.19 (0.68, 2.08)	0.541	1.18 (0.74, 1.88)	0.479	1.01 (0.46, 2.18)	0.988

Note. aOR = adjusted odds ratio; CI = confidence interval; Ref. = reference category. Models were estimated using survey-weighted (svy) logistic regression accounting for the complex survey design (strata, primary sampling units, and sampling weights). Reference categories are indicated in parentheses beneath each characteristic heading. Bold italicized headings denote categorical variable groupings; reference category odds = 1.00 (not shown). “—” indicates the variable was not included in or estimable for that model (e.g., omitted for collinearity or zero cell counts/subpopulation size). All reported p-values are two-tailed; statistical significance was set at p < 0.0

## References

[1] Miah MS. Stratified and geographic patterns of depression and anxiety in Mozambican women: Mental health policy implications. PLoS One. 2026;21(8):e0356265. doi: 10.1371/journal.pone.0356265 42616811 PMC13489479

