# Supplementary material for: Stratified and geographic patterns of depression and anxiety in Mozambican women: Mental health policy implications
Source: PLoS One. 2026 Aug 19;21(8):e0356265. doi: 10.1371/journal.pone.0356265 (PMC13489479; doi:10.1371/journal.pone.0356265)
Supplement: Supplementary file 1 [file pone.0356265.s004.pdf]

Date: July 3, 2026

Dear Dr. Jennifer Yourkavitch
Academic Editor
PLOS ONE

Re: Revised Manuscript Submission

Thank you for the opportunity to revise and resubmit our manuscript entitled:

“Stratified and Geographic Patterns of Depression and Anxiety in Mozambican Women: Maternal Mental Health Policy Implications”

We sincerely appreciate the time and constructive feedback provided by the Editor and reviewers, which has significantly improved the quality and clarity of the manuscript.

In response to the comments, we have carefully revised the manuscript and addressed all issues in detail in a separate document titled **“Response to Reviewers,”** where each comment is reproduced followed by our point-by-point response and corresponding changes in the manuscript.

We have also submitted:

- A revised manuscript with tracked changes
- A clean final version of the manuscript
- Updated figures and tables, where applicable

Key revisions include:

- Improved terminology consistency throughout the manuscript
- Revised Results section for clearer structure and analytical presentation
- Reframed “spatial analysis” as “geographic/provincial analysis” for methodological accuracy
- Improved figure presentation and interpretation
- Updated maps using quartile-based classification
- Clarified reference groups and model specifications
- Strengthened discussion and theoretical framing
- Enhanced methodological clarity and variable definitions
- Improved limitations section
- Standardized statistical reporting
- Strengthened conclusions and policy relevance

We have also ensured full compliance with PLOS ONE formatting and submission requirements.

Additional notes

We would like to inform you that the corresponding author’s email address has been updated, and additional relevant references have been included in the revised manuscript.

We also apologize for the delayed submission. The revised manuscript was originally planned for submission on June 25, 2026; however, due to an unexpected electricity outage, we were unable to complete the submission on time. We are submitting it at the earliest possible opportunity on June 26, 2026.

We hope the revised manuscript adequately addresses all concerns and is now suitable for publication in PLOS ONE. We greatly appreciate your consideration and the opportunity to improve our work.

Sincerely,
Md Salek Miah
Corresponding Author
Department of Statistics
Shahjalal University of Science & Technology (SUST), Bangladesh
Email: saleksta@gmail.com
ORCID: 0009-0005-5973-461X

Requirement 1: PLOS ONE formatting and file naming

Editor Comment:

Please ensure that your manuscript meets PLOS ONE's style requirements, including those for file naming. The PLOS ONE style templates can be found at

https://journals.plos.org/plosone/s/file?id=wjVg/PLOSOne_formatting_sample_main_body.pdf and

https://journals.plos.org/plosone/s/file?id=ba62/PLOSOne_formatting_sample_title_authors_affiliations.pdf

Response:
Thank you for this comment. We have carefully reviewed the PLOS ONE formatting guidelines using the official templates provided by the journal. Based on these requirements, we have thoroughly revised the manuscript to ensure full compliance with PLOS ONE style.

Specifically, we have:

- Reformatted the manuscript according to PLOS ONE structural guidelines
- Revised title page, author names, and institutional affiliations to match journal requirements
- Standardized headings, subheadings, tables, and references
- Corrected and updated file naming according to submission system requirements
- Ensured consistency in formatting throughout the manuscript

These revisions ensure full adherence to PLOS ONE formatting and file naming standards.

Requirement 2: Data availability statement and DHS dataset policy

Editor Comment:

In the online submission form, you indicated that [The data underlying the results presented in this study are publicly available from the Demographic and Health Surveys (DHS) Program, accessible at https://dhsprogram.com/data/available-datasets.cfm Researchers must register and request access to the Mozambique 2022–2023 DHS dataset to use these data. All analyses reported in this manuscript were conducted using the de-identified data provided by DHS.].

All PLOS journals now require all data underlying the findings described in their manuscript to be freely available to other researchers, either 1. In a public repository, 2. Within the manuscript itself, or 3. Uploaded as supplementary information.

This policy applies to all data except where public deposition would breach compliance with the protocol approved by your research ethics board. If your data cannot be made publicly available for ethical or legal reasons (e.g., public availability would compromise patient privacy), please explain your reasons on resubmission and your exemption request will be escalated for approval.

Response:
Thank you for this important clarification regarding PLOS ONE data availability policy. We confirm that the data used in this study are derived from the Mozambique 2022–2023 Demographic and Health Survey (DHS) dataset, which is publicly accessible upon registration and approval through the DHS Program website (https://dhsprogram.com/data/available-datasets.cfm).

However, access to the DHS microdata is governed by strict data use agreements, and researchers are not permitted to publicly redistribute the raw dataset in an open repository.

To comply with PLOS ONE policy, we have:

- Revised the Data Availability Statement to clearly state controlled access via DHS
- Confirmed that the dataset can be obtained through DHS registration and approval
- Provided all derived datasets used in the analysis as Supporting Information (S1 File)
- Included all data processing and statistical analysis code to ensure full reproducibility

We respectfully request an exemption from full public deposition of the raw DHS microdata due to these contractual restrictions. However, all results are fully reproducible using the provided code once the dataset is obtained through DHS approval.

Requirement 3: Figures 5, 6, and S1 – copyright and map-related concerns

Editor Comment:

We note that Figures 5, 6, and S1 in your submission contain map images which may be copyrighted. All PLOS content is published under the Creative Commons Attribution License (CC BY 4.0), which means that the manuscript, images, and Supporting Information files will be freely available online, and any third party is permitted to access, download, copy, distribute, and use these materials in any way, even commercially, with proper attribution. For these reasons, we cannot publish previously copyrighted maps or satellite images created using proprietary data, such as Google software (Google Maps, Street View, and Earth). We require you to either (1) present written permission from the copyright holder to publish these figures under CC BY 4.0 or (2) remove/replace the figures.

Response:

Thank you for raising this important concern. We confirm that Figures 5, 6, and S1 are entirely original figures created by the authors using R (RStudio). No copyrighted maps, satellite imagery, or proprietary mapping tools such as Google Maps, Google Earth, or similar platforms were used in this study.

The spatial data used in the analysis were obtained from official DHS Program shapefiles, which are publicly available for research use. All spatial analysis and visualization were conducted independently using open-source R packages, including sf, ggplot2, and tmap.

Therefore:

- All figures are original creations of the authors
- No copyrighted or restricted map sources were used
- The figures fully comply with CC BY 4.0 licensing requirements
- No external permissions are required

Requirement 4: Supporting Information captions

Editor Comment: *Please include captions for your Supporting Information files at the end of your manuscript, and update any in-text citations to match accordingly.*

Response: [Page no: 41, line no:965-967]

Thank you for this comment. We have added complete captions for all Supporting Information files at the end of the revised manuscript. Additionally, all in-text citations related to Supporting Information have been carefully checked and updated to ensure full consistency with PLOS ONE formatting requirements.

RESPONSE TO REVIEWER 1 RESPONSE 1

(ABSTRACT – AGE DISAGGREGATION)

Reviewer Comment 1:

It will be helpful to have age disaggregation bands available here as otherwise it is challenging to interpret the findings.

Author Response: [Page no: 2, line no: 30, page no:11, line no: 238-239]

We sincerely thank the reviewer for this valuable suggestion. We would like to clarify that the age range of 15–49 years represents the standard reproductive age population in Demographic and Health Survey (DHS)-based studies and is used as the study eligibility criterion rather than a covariate in the analytical models. In response to the reviewer's suggestion, we have improved clarity in the abstract methodology section by explicitly stating that the study population consists of ever-married women aged 15–49 years. In addition, we briefly indicate that age was categorized into standard DHS reproductive age groups (15–19,

20–24, 25–29, 30–34, 35–39, 40–44, and 45–49 years) for descriptive interpretation to improve understanding of the sample structure.

This revision enhances interpretability while maintaining the correct methodological distinction between study population definition and analytical covariates.

Change made:

- 15–49 defined as reproductive age study population
- Age bands added for descriptive clarity only

RESPONSE 2 (ABSTRACT – DHS VARIABLES / SCALES CLARIFICATION)

Reviewer Comment 2:

Also, it is clear that the DHS was used to gather PHQ-9 and GAD-7 data. Were additional measures also gathered from the DHS? Please specify other scales such as women's empowerment, household asset ownership, and internet usage.

Author Response: [page no: 2, line no: 32-34, Page no: 10, line no: 216-222]

We sincerely thank the reviewer for this important comment. In response, we have clarified the abstract methodology to explicitly describe all data sources and variables used in the study.

We confirm that, in addition to PHQ-9 and GAD-7 for assessing depression and anxiety, the study also utilized multiple standardized Demographic and Health Survey (DHS)-derived variables. These included women's empowerment indicators (based on participation in household decision-making domains), household asset ownership (constructed from ownership of selected durable goods), and internet usage (measured as self-reported use within the previous 12 months).

These variables were derived from standard DHS questionnaires and recode datasets, ensuring methodological consistency and comparability across studies.

RESPONSE TO REVIEWER 1 (COMMENT 3)

Reviewer Comment:

It would be useful to understand the percentage of comorbid depression and anxiety.

Author Response: [Page no: 32, line no: 708-710]

We sincerely thank the reviewer for this valuable suggestion. We agree that reporting comorbidity between anxiety and depressive symptoms would provide additional epidemiological insight into the mental health burden among the study population.

However, in the present analysis, comorbidity (simultaneous occurrence of PHQ-9 ≥10 and GAD-7 ≥10) was not included as a predefined outcome measure in the analytical framework. Therefore, this estimate was not reported in the current version of the manuscript.

We acknowledge this as an important limitation and have added it to the Limitations section. We also recommend that future studies using this dataset should explicitly explore comorbid mental health

conditions to provide a more comprehensive understanding of mental health burden among reproductive-aged women.

RESPONSE TO REVIEWER 1 (COMMENT 4)

Reviewer 1 – Comment 4:

In your findings for the age-bands, it will be helpful to specify which group had the highest odds of anxiety, and then next highest.

Author Response [Abstract: Findings Section, Page 2-3, Lines 40-44]

We sincerely thank the reviewer for this valuable suggestion. We agree that explicitly identifying the age groups with the highest and subsequent highest odds of anxiety would improve the clarity and interpretability of the findings presented in the abstract.

In response, we revised the Findings section to clearly indicate the ranking of age groups according to the magnitude of their associations with anxiety and depressive symptoms. Specifically, women aged 35–39 years exhibited the highest odds of both anxiety (aAOR = 2.06, 95% CI: 1.48–2.87) and depressive symptoms (aAOR = 1.49, 95% CI: 1.07–2.08). Women aged 45–49 years showed the second-highest odds of anxiety (aAOR = 1.58, 95% CI: 1.12–2.23), while the second-highest odds of depressive symptoms were also observed among women aged 45–49 years (aAOR = 1.32, 95% CI: 0.92–1.89), compared with women aged 15–19 years.

These revisions improve the readability of the abstract and allow readers to more readily identify the age groups at greatest risk without comparing multiple effect estimates across categories.

RESPONSE TO REVIEWER 1 (COMMENT 5)

Reviewer 1 – Comment 5:

Suggest extending the conclusion beyond integrated policies. While this is an important start, without supportive health systems, skilled clinicians, and available services, the policies will not address MMH challenges experienced by Mozambican girls and women.

Author Response [Abstract: Conclusion Section, Page 3-4, Lines 63-66]

We sincerely thank the reviewer for this insightful and constructive suggestion. We agree that policy development alone is insufficient to effectively address maternal mental health challenges. In response, we have expanded the conclusion to emphasize the importance of supportive health systems, access to mental health services, trained healthcare professionals, and community-based interventions alongside integrated and equity-focused policies.

The revised conclusion now highlights that meaningful improvements in maternal mental health outcomes will require not only policy commitment but also adequate service availability, workforce capacity, and implementation mechanisms to ensure timely identification and support of women experiencing anxiety and depression.

This revision strengthens the public health relevance and practical implications of the study findings.

RESPONSE TO REVIEWER 1 (COMMENT 6)

Reviewer 1 – Comment 6:

Suggest ending the first sentence on line 67 after disease and starting a new sentence with "In 2021…"

Author Response [Introduction, Page 4, Lines 84-85]

We sincerely thank the reviewer for this helpful editorial suggestion. In response, we have revised the opening paragraph of the Introduction by separating the original sentence into two distinct sentences. Specifically, the first sentence now ends after "global burden of disease," and a new sentence beginning with "In 2021…" has been introduced.

This revision improves sentence structure, readability, and overall clarity of the Introduction.

RESPONSE TO REVIEWER 1 (COMMENT 7)

Reviewer 1 – Comment 7:

Line 69…should be "worsen" and "elevate the risk of comorbid…"

Author Response [Introduction, Page 4, Line 86-87]

We sincerely thank the reviewer for this helpful language correction. In response, we have revised the sentence as suggested. The text now states that depression and anxiety "worsen quality of life, reduce productivity, and elevate the risk of comorbid physical health conditions."

This revision improves grammatical accuracy and enhances the clarity of the statement.

Reviewer 1 – Comment 8:

Just a note that if you are including girls as young as 15 years old, so I would encourage the authors to refer to the study population as "girls and women of reproductive age".

Author Response [Methodology, Page 10, Lines 207-208]

We sincerely thank the reviewer for this thoughtful suggestion. We acknowledge that the study population includes adolescents aged 15–19 years in addition to adult women.

However, we have retained the term "women of reproductive age" throughout the manuscript to maintain consistency with the standard terminology used in Demographic and Health Surveys (DHS), demographic research, and international reproductive health literature, where females aged 15–49 years are conventionally classified as women of reproductive age, regardless of age subgroup.

To improve clarity, we have explicitly stated in the Methods section that the study population comprises ever-married women aged 15–49 years, including adolescents aged 15–19 years. We believe this approach preserves consistency with established demographic reporting standards while clearly describing the study population.

RESPONSE TO REVIEWER 1 (COMMENT 9)

Reviewer 1 – Comment 9:

Line 73: Depression and anxiety disproportionately affect girls and women in LMIC…

Author Response [Introduction, Page 5, Line 92-95]

We sincerely thank the reviewer for this helpful suggestion. We agree that the statement should more clearly reflect the population focus of the study.

In response, we have revised the sentence to emphasize the burden of depression and anxiety among women of reproductive age in low- and middle-income countries (LMICs), while maintaining consistency with the terminology used throughout the manuscript and the standard demographic and Demographic and Health Survey (DHS) literature.

This revision improves the alignment between the background information and the study population under investigation.

RESPONSE TO REVIEWER 1 (COMMENT 10)

Reviewer 1 – Comment 10:

Line 76: Would encourage including lack of supportive policies and guidelines, mental health stigma, lack of specialized services as other issues.

Author Response [Introduction, Page 5, Line 96-98]

We sincerely thank the reviewer for this valuable suggestion. We agree that broader structural and health-system factors play an important role in shaping mental health outcomes among women in low-resource settings.

In response, we have revised the Introduction to acknowledge additional barriers to mental healthcare, including inadequate mental health policies and implementation frameworks, mental health stigma, shortages of trained mental health professionals, and limited availability of specialized mental health services. These additions provide a more comprehensive overview of the contextual challenges influencing anxiety and depression among women of reproductive age in low-resource settings.

This revision strengthens the background section and better contextualizes the public health significance of maternal mental health challenges in Mozambique.

RESPONSE TO REVIEWER 1 (COMMENT 11)

Reviewer 1 – Comment 11:

If the authors are going to discuss internet access in the introduction, also suggest drawing in specific examples from the literature in regards to MMH specifically, otherwise this doesn't seem a natural fit within this section.

Author Response [Introduction, Page 5, Lines 103-105]

We sincerely thank the reviewer for this insightful suggestion. We agree that the relevance of internet access to mental health among women of reproductive age should be more clearly justified.

In response, we have revised the Introduction by incorporating evidence from previous research demonstrating that internet use may influence mental health through improved access to health information,

social support networks, and digital health resources. We also added a recent DHS-based study reporting lower odds of anxiety and depression among internet users compared with non-users.

This revision strengthens the rationale for including internet use as a potential determinant of anxiety and depression in the present study.

RESPONSE TO REVIEWER 1 (COMMENT 12)

Reviewer 1 – Comment 12:

Note that lines 81–83 state that there is little information on mental health twice. Also be sure throughout to note that the topic is specifically maternal mental health which is an even further neglected area of mental health.

Author Response [Introduction, Page 5-6, Lines 106-112]

We sincerely thank the reviewer for this valuable observation. We agree that the previous text contained repetitive statements regarding the limited availability of mental health evidence. In response, we have revised the Introduction to remove redundancy and more clearly emphasize the specific evidence gap in maternal mental health rather than mental health in general.

The revised text now highlights that, although health research in Mozambique has primarily focused on maternal and child health, infectious diseases, and nutritional outcomes, maternal mental health remains a relatively underexplored area. This revision improves clarity and strengthens the justification for the present study by emphasizing the specific maternal mental health evidence gap in Mozambique.

This change has been incorporated throughout the manuscript where appropriate to maintain consistency with the study focus.

RESPONSE TO REVIEWER 1 (COMMENT 13)

Reviewer 1 – Comment 13:

I have yet to see information on the influence of access to media on MMH; it would be helpful to include that specific relationship on lines 90–91.

Author Response [Introduction, Page 6, line no:118-122, 126-129]

We sincerely thank the reviewer for this helpful suggestion. We agree that the relationship between media access and mental health should be more clearly explained rather than simply listed as a potential determinant.

In response, we have revised the Introduction to briefly describe the potential role of media access in influencing mental health among women of reproductive age. Specifically, we note that access to media may improve exposure to health information, increase awareness of available healthcare services, promote mental health literacy, and facilitate health-seeking behaviors, all of which may influence anxiety and depression outcomes. We have also included supporting literature to strengthen the rationale for considering media access as a potential determinant in the present study.

This revision improves the conceptual framework and better aligns the Introduction with the study objectives and analytical variables.

RESPONSE TO REVIEWER 1 (COMMENT 14)

Reviewer 1 – Comment 14:

Suggest ending the introduction with a sentence or two on how this methodological framework may be useful for other countries interested in developing responsive and data-driven MMH policies.

Author Response [Introduction, Page 8, Lines 166-175]

We sincerely thank the reviewer for this insightful suggestion. We agree that highlighting the broader applicability of the study framework strengthens the significance of the Introduction.

In response, we have revised the concluding paragraph of the Introduction to emphasize that the analytical framework employed in this study—including survey-weighted multivariable regression, spatial analysis, and residence-stratified analyses integrating sociodemographic, reproductive, household, and environmental factors—may be adapted by other low- and middle-income countries to generate context-specific evidence for developing responsive, data-driven maternal mental health policies and targeted interventions.

This addition highlights the broader public health relevance and potential transferability of our methodological approach beyond Mozambique.

RESPONSE TO REVIEWER 1 (COMMENT 15)

Reviewer 1 – Comment 15:

Line 142, what does the two areas missed refer to?

Author Response [Methods – Sampling Design and Sample Size, Page 10, Lines 205]

We sincerely thank the reviewer for identifying this ambiguity. We agree that the phrase "two areas were missed" was unclear.

In response, we have revised the text to explicitly clarify that the missed areas refer to two selected enumeration areas (EAs) that were not successfully surveyed during data collection. This revision improves the clarity and accuracy of the sampling description while maintaining consistency with the Mozambique Demographic and Health Survey (MDHS) sampling methodology.

RESPONSE TO REVIEWER 1 (COMMENT 16)

Reviewer 1 – Comment 16:

To confirm, this is mental health in general among women of reproductive age? These women have not necessarily had a pregnancy?

Author Response [Methodology, Page 10, Lines 207-210]

We sincerely thank the reviewer for this important observation. We agree that the study population should be more clearly described.

This study examined symptoms of anxiety and depression among ever-married women of reproductive age (15–49 years) using nationally representative MDHS 2022–2023 data. The study population was not

restricted to women who were currently pregnant or who had previously experienced pregnancy. Rather, it included all eligible ever-married women within the reproductive age range, regardless of their pregnancy status or reproductive history. Pregnancy-related characteristics were considered as explanatory variables where applicable.

To improve clarity, we have revised the Methods section to explicitly describe the study population and clarify that the analyses focus on mental health among women of reproductive age rather than exclusively on pregnant or postpartum women.

RESPONSE TO REVIEWER 1 (COMMENT 17)

Reviewer 1 – Comment 17:

How was covariate data gathered? Was this all available in the DHS? If not, please specify tools used and how data was gathered if means were required outside of standard DHS. Beyond the statistical analysis, much more information is needed on how all of these measures were collected.

Author Response [Methods – Covariates, Page 11-13, Lines 235–274]

We sincerely thank the reviewer for this important comment. We agree that the Methods section should provide greater detail regarding the collection and derivation of covariate data.

In response, we have substantially expanded the Covariates subsection to clarify that all explanatory variables were obtained from the Mozambique Demographic and Health Survey (MDHS) 2022–2023 dataset. No additional data collection tools or external measurement instruments were used. We now describe the categorization of socio-demographic, reproductive, household, and environmental variables and provide detailed explanations of how composite variables, including women's decision-making autonomy, intimate partner violence (IPV) justification, household asset ownership, media access, and internet use, were derived from standard DHS questionnaire items and classifications.

These revisions improve the transparency, reproducibility, and methodological clarity of the study.

RESPONSE TO REVIEWER 1 (COMMENT 18)

Reviewer 1 – Comment 18:

Starting on line 174. I am curious as to whether HIV status and/or migration status was also considered as variables given these are also widely recognized risk factors.

Author Response [Methodology, Page 14, Lines 290]

We sincerely thank the reviewer for this thoughtful suggestion. In the present study, covariates were selected based on previous literature and the availability of variables within the Mozambique Demographic and Health Survey (MDHS) 2022–2023 dataset.

To avoid potential ambiguity, we have also clarified in the revised manuscript that the variable refers to intimate partner violence (IPV) rather than HIV status.

Although intimate partner violence (IPV) was included as a covariate, HIV status and migration status were not included in the final analysis because these variables were not available for all women in the analytical sample and therefore could not be consistently incorporated into the survey-weighted regression models.

We have clarified the variable selection process in the Methods section and acknowledge that future studies incorporating these factors may provide additional insights into the determinants of anxiety and depression among women of reproductive age.

RESPONSE TO REVIEWER 1 (COMMENT 19)

Reviewer 1 – Comment 19:

Line 215. Please specify what higher education refers to.

Author Response [Results, Page 16, Lines 342–344]

We sincerely thank the reviewer for this helpful suggestion. We agree that the educational categories should be described more explicitly to avoid ambiguity.

In response, we have revised the Results section to report the distribution of women's educational attainment according to the predefined categories used in the analysis. Specifically, we now state that 69.2% of women had below primary education, 28.1% had incomplete secondary education, and 2.7% had secondary or higher education.

This revision improves the clarity and consistency of the descriptive findings with the educational categories presented in Table 1.

RESPONSE TO REVIEWER 1 (COMMENT 20)

Reviewer 1 – Comment 20:

Line 216. Please clarify what "justified intimate partner violence" refers to. Did they believe that 2/3rds of women who experienced IPV thought that it was excusable? Or among women in general? More specificity here will be important to understand the statement.

Author Response [Methodology, Covariates section: Page 12, Lines 252-257]

We sincerely thank the reviewer for this important observation. We agree that the previous wording could be misinterpreted as referring to women who had experienced intimate partner violence.

In response, we have revised the Results section to clarify that this variable represents women's attitudes toward the justification of wife-beating, derived from standard DHS questions assessing whether a husband is justified in beating his wife under specific circumstances. We now explicitly state that 81.3% of women in the study population justified wife-beating in at least one specified circumstance, rather than referring to women who had experienced IPV.

This revision improves the clarity and interpretation of the descriptive findings.

RESPONSE TO REVIEWER 1 (COMMENT 21)

Reviewer 1 – Comment 21:

Line 217, What are indicators of women's empowerment (it would help if the information were available in the methods).

Author Response [Methods – Covariates, Page 12, Lines 248-252]

We sincerely thank the reviewer for this valuable suggestion. We agree that the term "women's empowerment" was too broad and that its indicators should be clearly defined.

In response, we have replaced the term **"women's empowerment"** with the more specific term **"women's decision-making autonomy"** throughout the manuscript. We also expanded the **Covariates** subsection in the Methods to describe how this variable was derived from the standard MDHS questionnaire. Specifically, women's decision-making autonomy was assessed based on participation in decisions regarding their own healthcare, major household purchases, and visits to family or relatives. Women who participated alone or jointly with their partner in at least one of these decisions were classified as having decision-making autonomy, whereas those who did not participate in any of these decisions were classified as having no decision-making autonomy.

This revision provides a clearer operational definition of the variable and improves the transparency and reproducibility of the study.

RESPONSE TO REVIEWER 1 (COMMENT 22)

Reviewer 1 – Comment 22:

Line 218. What does improved sanitation refer to? Running water? Toilet in the house?

Author Response [Methodology – Covariates, Page 13, Lines 270-272]

We sincerely thank the reviewer for this helpful comment. We agree that the term **"improved sanitation"** could be ambiguous without an accompanying definition.

In response, we revised the descriptive Results section to improve clarity and removed the statement referring to improved sanitation. We also expanded the **Covariates** subsection in the Methods to clarify that household water source and sanitation facilities were classified as **improved** or **unimproved** according to the standard Mozambique Demographic and Health Survey (MDHS)/DHS definitions.

This revision improves the clarity and consistency of the manuscript.

RESPONSE TO REVIEWER 1 (COMMENT 23)

Reviewer 1 – Comment 23:

For lines 217–222, in the methodology it will be important to have the various scales that were used to understand how to interpret all of these results.

Author Response [Methodology – Covariates, Page 12-13, Lines : 257-274]

We sincerely thank the reviewer for this valuable suggestion. We agree that clearer operational definitions of the explanatory variables are essential for interpreting the descriptive and multivariable findings.

In response, we substantially expanded the **Covariates** subsection in the Methods. We now explicitly state that all covariates were extracted from the standardized Mozambique Demographic and Health Survey (MDHS) 2022–2023 questionnaire and household datasets, and that no additional data collection or external assessment tools were used. We further provide detailed operational definitions and categorization of all explanatory variables, including women's decision-making autonomy, attitudes toward the justification of intimate partner violence (IPV), household asset ownership, media access, internet use, reproductive

characteristics, and household and environmental factors, based on standard DHS questionnaire items and classifications.

These revisions improve the transparency, reproducibility, and interpretability of the study findings.

RESPONSE TO REVIEWER 1 (COMMENT 24)

Reviewer 1 – Comment 24:

What was the % of comorbid anxiety and depression? Is this not possible to ascertain from the DHS? If not, please include this in the limitations.

Author Response [Limitations, Page 32, Lines 708–710]

We sincerely thank the reviewer for this thoughtful suggestion. We agree that information on the co-occurrence of anxiety and depressive symptoms would provide additional clinical and public health insight.

However, the present study was designed to examine anxiety and depression as separate outcomes, and the prevalence of comorbid anxiety and depression was not estimated as part of the predefined analytical framework. In response to the reviewer's suggestion, we have added this point to the **Limitations** section, acknowledging that the co-occurrence of anxiety and depressive symptoms was not assessed in the present study and recommending that future research investigate this important aspect using the MDHS data.

This revision acknowledges the limitation and identifies an important direction for future research.

RESPONSE TO REVIEWER 1 (COMMENT 25)

Reviewer 1 – Comment 25:

The abstract says that women in their mid-30s and then again in their mid-40s experienced the highest odds of mental health symptoms, but lines 224–225 say that prevalence of symptoms was highest among young people. More clarification is needed to understand this conflicting data.

Author Response [Results, Page 18, Lines 393–397]

We sincerely thank the reviewer for this important observation. We agree that the distinction between descriptive prevalence and adjusted associations was not sufficiently clear in the original manuscript.

In response, we have revised both the Abstract and the Results section to clearly distinguish these findings. The Results section now explicitly reports the **survey-weighted prevalence** of anxiety and depressive symptoms by age group, showing that prevalence was highest among women aged 20–24 years. In contrast, the Abstract presents findings from the **survey-weighted multivariable** logistic regression models, where women aged 35–39 years and 45–49 years exhibited higher adjusted odds of anxiety and/or depressive symptoms relative to the reference group after controlling for potential confounding factors.

These revisions clarify that the descriptive prevalence estimates and adjusted odds ratios address different epidemiological questions and are therefore not contradictory.

RESPONSE TO REVIEWER 1 (COMMENT 26)

Reviewer 1 – Comment 26:

Line 226. Please clarify what 'early childbirth' refers to. Assume this means younger age when gave birth, and not pre-term birth?

Author Response [Methodology – Covariates, Page 11, Lines 239-242]

We sincerely thank the reviewer for this helpful comment. We agree that the term **"early childbirth"** could be misinterpreted as referring to preterm birth rather than maternal age at first birth.

In response, we have revised the Results section by replacing **"early childbirth"** with **"early age at first birth (10–17 years)"**, consistent with the definition provided in the Methods section. This revision makes it explicit that the variable refers to the woman's age at first birth rather than gestational age or preterm birth. This change improves the clarity and consistency of the manuscript.

RESPONSE TO REVIEWER 1 (COMMENT 27)

Reviewer 1 – Comment 27:

Also, early childbirth (if referring to young age), early sexual debut and early cohabitation all represent a similar phenomenon – just noting that these are similar and wondering if this should be teased out any further to understand specifically if its early motherhood (adolescents are among the highest risk for a perinatal mental health disorder), or if simply cohabitating places a girl at risk.

Author Response [Methodology – Covariates, Page 11, Lines 239-242, Discussion, Page 30, Lines 669–674]

We sincerely thank the reviewer for this insightful comment. We agree that early age at first birth, early sexual debut, and early cohabitation are closely related reproductive life-course events and may share common underlying social and behavioral pathways.

In the present study, these variables were treated as distinct covariates because they represent different reproductive and social transitions captured in the standardized MDHS 2022–2023 questionnaire. Accordingly, age at first birth, age at first sex, and age at cohabitation were operationally defined and analyzed separately in the multivariable regression models to estimate their independent associations with anxiety and depressive symptoms while adjusting for other covariates.

We have expanded the Methods section to clearly describe the categorization and definitions of these variables and added clarification in the Discussion acknowledging that these reproductive life-course events are interrelated. We also note that future longitudinal studies are warranted to disentangle their temporal and causal relationships and to determine whether early motherhood, early sexual debut, or early cohabitation has the greatest influence on mental health among women of reproductive age.

RESPONSE TO REVIEWER 1 (COMMENT 28)

Reviewer 1 – Comment 28:

Line 230. Please clarify the similar trends across partners' education, etc. Does that mean that girls and women experienced more mental health symptoms based upon their partners' education?

Author Response [Methods – Covariates, Page 11, Lines 242-243]

We sincerely thank the reviewer for this helpful comment. In response, we revised the Results section by removing the statement referring to "similar trends across partners' education" and instead clearly reporting the descriptive distribution of mental health symptoms according to women's educational attainment. We also standardized the terminology in the Methods section by referring to the variable as **husband's education**, consistent with the MDHS questionnaire.

These revisions improve the clarity and consistency of the manuscript and avoid potential misinterpretation.

RESPONSE TO REVIEWER 1 (COMMENT 29)

Reviewer 1 – Comment 29:

Line 234, a new paragraph should start with "Among the…"

Author Response [Results, Page 19, Lines 403]

We sincerely thank the reviewer for this helpful suggestion. In response, we reorganized the descriptive Results into thematic paragraphs. The section has been revised to introduce reproductive health and psychosocial characteristics under a new descriptive heading ("Other notable clusters were reproductive health and psychosocial symptoms"), thereby improving the logical flow and readability of the Results.

This revision enhances the overall organization and clarity of the manuscript.

RESPONSE TO REVIEWER 1 (COMMENT 30)

Reviewer 1 – Comment 30:

Line 236, aren't three or more children and multiple pregnancies the same? Or do multiple pregnancies refer to twins and triplets?

Author Response [Methodology, Page 12-13, Lines 265-268]

We sincerely thank the reviewer for this helpful observation. We agree that the previous wording could be confusing because the terms "three or more children" and "multiple pregnancies" may be interpreted differently.

In response, we revised the descriptive Results section and removed this statement to avoid ambiguity. The Results now focus on clearly presented descriptive findings, while the operational definitions of reproductive variables are provided in the Methods section.

This revision improves the clarity and interpretability of the manuscript.

RESPONSE TO REVIEWER 1 (COMMENT 31)

Reviewer 1 – Comment 31:

Line 236. What does inadequate ANC visits refer to? Please include a number – e.g., did not meet the threshold # of…

Author Response [Methodology – Covariates, Page 12, Lines 247]

We sincerely thank the reviewer for this helpful suggestion. In response, we revised the manuscript by removing the descriptive statement referring to "inadequate ANC visits" from the Results section. We also

expanded the **Covariates** subsection in the Methods to clearly define antenatal care attendance according to the standardized MDHS variable, categorizing women as having received **≥4 antenatal care visits** or **<4 antenatal care visits**, consistent with the survey classification used in the analysis.

These revisions improve the clarity and interpretability of the manuscript.

RESPONSE TO REVIEWER 1 (COMMENT 32)

Reviewer 1 – Comment 32:

Line 236–237. Pressure to become pregnant from who?

Author Response [Methodology – Covariates section, Page 12, Lines 245-247]

We sincerely thank the reviewer for this important comment. In response, we have revised the Methods section to clarify that this variable was derived directly from the standardized MDHS 2022–2023 questionnaire and explicitly identified the source of the reported pressure, consistent with the survey definition. This clarification improves the transparency and interpretation of the covariate used in our analysis.

RESPONSE TO REVIEWER 1 (COMMENT 33)

Reviewer 1 – Comment 33:

Line 237. The sentence about previous pregnancy loss is confusing. It currently reads that women who had ***not*** *experienced a previous loss were more likely to experience depression and anxiety. This is a surprising finding given that previous pregnancy loss and stillbirth are risk factors for perinatal mental health disorders. More clarification is needed.*

Author Response [Results, Methodology – Covariates, Page 12, Lines 267, Discussion: Page no: 31 , line no: 677-679]

We sincerely thank the reviewer for this careful observation. In response, we revised the Results section by removing the ambiguous statement and presenting the descriptive findings more clearly. We also clarified the operational definition of pregnancy loss in the Methods section. These revisions improve the accuracy and prevent misinterpretation of the findings. We appreciate the reviewer for identifying this issue.

RESPONSE TO REVIEWER 1 (COMMENT 34)

Reviewer 1 – Comment 34:

Women abstaining from sex?

Author Response [Methodology – Covariates sections, page no:11-12, linen no: 244-245]

We sincerely thank the reviewer for this helpful comment. In response, we have revised the Methods section to clarify that **current sexual abstinence** was a binary covariate obtained directly from the standardized MDHS 2022–2023 questionnaire and categorized as **"Yes"** or **"No"**. We also expanded the covariate descriptions throughout the Methods section to provide clearer operational definitions for all variables derived from the MDHS dataset. These revisions improve the transparency and reproducibility of the study methodology.

RESPONSE TO REVIEWER 1 (COMMENT 35)

Reviewer 1 – Comment 35:

Please name the specific religious grouping under study.

Author Response [Methodology– Covariates section, Page 13, Lines 273-274]

We sincerely thank the reviewer for this helpful suggestion. We agree that the religious categories should be explicitly specified for clarity.

In response, we have revised the Methods section to clearly list the religious groupings included in the analysis. Specifically, religion was categorized as **Catholic, Islam, Zion, Evangelical/Pentecostal, and Other**, consistent with the standardized MDHS 2022–2023 classifications.

This revision improves the transparency and interpretability of the study methodology.

RESPONSE TO REVIEWER 1 (COMMENT 36)

Reviewer 1 – Comment 36:

Line 244, had more than twice the odds in comparison to which other age group? Again, these age groupings are confusing with the information reported in the abstract. As it reads right now, it looks as though the researchers have lumped girls and women 15–39 together to indicate twice the odds of anxiety (in comparison to who?), and higher odds of depression, and then also states that women 45–49 also have higher odds of anxiety. Again, across all of these, what is the reference or comparison group to whom the higher odds are being compared to?

Author Response [Results, Page 19, Lines 409–414]

We sincerely thank the reviewer for this valuable comment. We agree that the previous wording did not clearly identify the reference category and could therefore lead to misinterpretation.

In response, we have revised both the Abstract and Results sections to explicitly state that **women aged 15–19 years served as the reference group** in the multivariable logistic regression analyses. We also clarified the comparisons for age at first sexual intercourse by specifying the corresponding reference category (≥18 years). These revisions ensure that all reported adjusted odds ratios are interpreted relative to their appropriate reference groups and improve the consistency and clarity of the manuscript.

RESPONSE TO REVIEWER 1 (COMMENT 37)

Reviewer 1 – Comment 37:

Line 255. Internet use is stated to report higher risk of anxiety, but line 233 says that symptoms were higher among those without internet access. This seems contradictory.

Author Response [Results, Page 20, Line 426-427]

We sincerely thank the reviewer for identifying this important inconsistency. In response, we carefully revised the Results section to clearly distinguish between descriptive prevalence estimates and adjusted associations. We also corrected the descriptive statement to ensure consistency with the reported findings. The descriptive results now accurately reflect the distribution of mental health symptoms, while the adjusted

regression results indicate that internet use was independently associated with higher odds of anxiety after controlling for potential confounding factors. These revisions improve the clarity of the Results section and eliminate the apparent contradiction.

RESPONSE TO REVIEWER 1 (COMMENT 38)

Reviewer 1 – Comment 38:

Line 262. These findings of higher odds of anxiety correlate to what was reported in the abstract. But how is this different from the findings of anxiety reported among girls? Are there sub-headings missing by location? The results as written are quite confusing.

Author Response [Results, Page 21, Line 457-462]

We sincerely thank the reviewer for this valuable observation. We agree that the original presentation of the Results section could make it difficult to distinguish between the overall multivariable findings and the residence-stratified analyses.

In response, we substantially revised this section to improve its organization and readability. The overall adjusted multivariable regression results are now presented first, followed by the residence-stratified analyses in separate paragraphs with clear transitions. We also consistently state the reference category (women aged 15–19 years) when reporting age-specific adjusted odds ratios.

These revisions clearly distinguish the national-level findings reported in the Abstract from the subsequent residence-stratified analyses and improve the overall flow and interpretation of the Results.

RESPONSE TO REVIEWER 1 (COMMENT 39)

Reviewer 1 – Comment 39:

Line 279, it appears that a bracket is missing.

Author Response

We sincerely thank the reviewer for carefully identifying this typographical error. The missing bracket has been corrected in the revised manuscript after thorough proofreading. We have also carefully reviewed the entire manuscript to identify and correct similar formatting and punctuation errors, thereby improving the overall presentation and readability.

RESPONSE TO REVIEWER 1 (COMMENT 40)

Reviewer 1 – Comment 40:

Line 287. Am I understanding correctly that this data is only rural women now, and early sexual debut was protective against experiencing anxiety? I would use caution with that terminology as its not clear that having sex at a young age is protective, but rather girls who had sex at a young age in rural areas experienced lower odds of depression in comparison to who?

Author Response [Results, Page no: 23, line no: 504- 505, Page 30, Line 668-673]

We sincerely thank the reviewer for this thoughtful observation. We agree that the previous wording could have been misinterpreted by implying that early sexual debut was protective against mental health symptoms. This was not our intended interpretation.

In response, we substantially revised the Results section and removed the ambiguous wording. The revised manuscript now presents the overall adjusted multivariable findings and clearly reports that women with **age at first sex <18 years** had significantly higher odds of both anxiety and depressive symptoms compared with women whose first sexual intercourse occurred at **≥18 years**, which served as the reference category. We also clarified the reference groups throughout the Results section to improve readability and prevent misinterpretation.

These revisions eliminate the previous ambiguity and ensure that the findings are interpreted appropriately.

RESPONSE TO REVIEWER 1 (COMMENT 41)

Reviewer 1 – Comment 41:

Line 293, the reference to toilet facilities is unclear.

Author Response [Methodology, Covariates section, Page 13, Line 271]

We sincerely thank the reviewer for this helpful comment. We agree that the terminology describing toilet facilities required clarification.

In response, we have revised both the Methodology and Results sections to explicitly state that sanitation facilities were classified as **improved** or **unimproved** according to the standard Mozambique Demographic and Health Survey (MDHS) definitions. In the Results section, we also specify that women living in households with **improved sanitation facilities** had lower odds of anxiety compared with those living in households with **unimproved sanitation facilities**. These revisions improve the clarity and interpretation of the sanitation-related findings.

RESPONSE TO REVIEWER 1 (COMMENT 42)

Reviewer 1 – Comment 42:

Line 311. What does household division refer to?

Author Response [Page 14, Line 302-306]

Thank you for identifying this ambiguity. We agree that the previous wording was unclear and could lead to confusion regarding the interpretation of the geographic findings.

In response, we have revised the Results section to provide a clearer description of the provincial distribution of anxiety and depressive symptoms. The revised text now reports that the highest prevalence was observed in Nampula, followed by Sofala, Zambézia, and Cabo Delgado, whereas the lowest prevalence occurred in the southern provinces, including Gaza, Inhambane, and Niassa.

We have also clarified that provincial adjusted odds ratios were interpreted relative to the reference province used in each regression model. Because of differences in model specification across analyses (Tables 3–5), the reference category for province was not identical in all models. Specifically, **Maputo City** was used as the reference category in the main multivariable models (Tables 3 and 4), whereas **Niassa** was used as the

reference category in selected stratified and outcome-specific models (Table 5) to improve interpretability within those analyses. This clarification has now been added to both the Methods and Results sections to facilitate interpretation and avoid confusion when comparing estimates across models.

RESPONSE TO REVIEWER 1 (COMMENT 43)

Reviewer 1 – Comment 43:

Early sexual debut now appears to be the greatest risk factor, but earlier was said to be protective in rural areas?

Author Response [Results, Page 19, Line 414-416]

We sincerely thank the reviewer for this careful observation. We agree that the previous wording created an apparent inconsistency.

In response, we substantially revised the Results section and removed the statement suggesting that early sexual debut was protective in rural areas. The revised multivariable analysis consistently shows that **age at first sexual intercourse <18 years** was associated with significantly higher odds of both anxiety and depressive symptoms compared with women whose first sexual intercourse occurred at **≥18 years** (reference category). The revised Results are now internally consistent and clearly distinguish the overall adjusted findings from other analyses, thereby eliminating the previous ambiguity.

RESPONSE TO REVIEWER 1 (COMMENT 44)

Reviewer 1 – Comment 44:

Line 315–316. Unclear what AUC and ROC refers to and what it means in the context of the results. What should the reader infer from this?

Author Response [Results, Page 25, Line 548-550]

We sincerely thank the reviewer for this valuable suggestion. We agree that the previous text did not sufficiently explain the interpretation of the receiver operating characteristic (ROC) curves and the area under the curve (AUC).

In response, we revised the Results section to briefly explain their interpretation. The revised text now states that both the anxiety and depression multivariable models demonstrated **moderate discriminatory ability (AUC = 0.68)**, indicating that the models were able to **moderately distinguish women with and without symptoms of anxiety and depression**. This additional explanation helps readers better understand the practical meaning of the ROC and AUC results.

RESPONSE TO REVIEWER 1 (COMMENT 45)

Reviewer 1 – Comment 45:

Overall comment on the Results section. It is quite long and confusing. Sub-headings are needed and a great deal of attention to clarify the different analyses which seem to currently overlap and contradict each other. There are also several spaces where the authors use "higher odds" but don't provide a reference/comparison group.

Author Response

We sincerely thank the reviewer for this comprehensive and constructive suggestion. In response, we have substantially revised the Results section by introducing appropriate sub-headings to distinguish the descriptive findings, multivariable regression analyses, residence-stratified analyses, spatial analyses, and model performance results. We also reorganized the text to improve the logical flow and eliminate overlapping or potentially contradictory descriptions between different analyses.

Furthermore, throughout the revised Results section, we have consistently specified the relevant reference categories (e.g., women aged 15–19 years, women with age at first sex ≥18 years, unimproved sanitation facilities, no household asset ownership, and Maputo Cidade province) whenever reporting adjusted odds ratios. These revisions substantially improve the clarity, readability, and interpretability of the Results section.

RESPONSE TO REVIEWER 1 (COMMENT 46)

Reviewer 1 – Comment 46:

Line 322. A definition for 'reproductive transitions' should be included within the methodology. It will be important to understand how much of this data is actually centered around the reproductive transitions once the reader has an idea what that transition refers to.

Author Response [Methodology, page 11-13, Discussion, Page 27, Line 593-599]

We sincerely thank the reviewer for this helpful observation. To avoid ambiguity, we have revised the opening paragraph of the Discussion by removing this terminology and instead summarizing the principal findings using the specific variables evaluated in our analyses (e.g., age, age at first sex, decision-making autonomy, and province). This revision improves clarity and ensures consistency with the variables defined in the Methods section.

RESPONSE TO REVIEWER 1 (COMMENT 47)

Reviewer 1 – Comment 47:

Line 332. This is the first reference that I've read regarding poor quality care. How was this ascertained? There was earlier mention of fewer ANC visits, but this seems different.

Author Response [Methods page no: 12, line no: 247]

We sincerely thank the reviewer for this important observation. We agree that the phrase *"poor quality antenatal care"* was not directly measured in the MDHS dataset and could therefore be misleading. The MDHS only includes information on the number of antenatal care (ANC) visits (categorized as <4 versus ≥4 visits) and does not assess the quality of ANC services. In response, we have removed this statement from the Discussion and revised the section to focus only on factors that were directly measured and supported by our multivariable analyses, including age, age at first sex, women's decision-making autonomy, province, and internet use. This revision improves consistency between the Discussion, Methods, and Results sections.

RESPONSE TO REVIEWER 1 (COMMENT 48)

Reviewer 1 – Comment 48:

I am not convinced that the internet has any meaningful impact based upon this study; it seems like a superficial measure with perhaps some correlations but not necessarily impacts. If the authors are to include internet within this study, suggest further explanation and justifications.

Author Response [Introduction page no: 5, line no: 103-105, Discussion, page no: 27, line no: 600-604, page no: 28, line no: 628-629, Page 29, Lines 631-633, 640-648, page no: 31, line no: 681-684]

We sincerely thank the reviewer for this thoughtful comment. We agree that the cross-sectional design of the MDHS does not allow causal inferences regarding internet use and mental health. Accordingly, we have revised both the Introduction and Discussion to better justify the inclusion of internet use and to avoid causal interpretations.

In the Introduction, we now explain that internet use was included as a contextual variable because previous studies have suggested that it may be associated with women's mental health through access to health information, social support, digital health resources, and exposure to online stressors. In the Discussion, we have revised the interpretation to emphasize that internet use was statistically associated with anxiety in our adjusted analyses but may represent a proxy for broader social and digital contextual factors rather than a direct causal determinant. We also acknowledge that further longitudinal studies are required to clarify the underlying mechanisms.

These revisions provide a stronger theoretical rationale while ensuring that our interpretations remain consistent with the observational nature of the study.

RESPONSE TO REVIEWER 1 (COMMENT 49)

Reviewer 1 – Comment 49:

The authors indicate that early sexual debut is the main risk factor but then state here that women over age 18 were more likely to have symptoms.

Author Response [Discussion, Page 30-31, Line 671-679]

We sincerely thank the reviewer for identifying this inconsistency. We agree that our previous wording could be confusing. The adjusted multivariable analyses used women whose age at first sexual intercourse was <18 years as the reference category; therefore, the reported odds ratios indicate that women whose first sexual intercourse occurred at ≥18 years had higher odds of anxiety and depressive symptoms relative to the reference group. To ensure consistency with the statistical analyses, we have revised the Discussion by removing the statement that early sexual debut was the principal risk factor and now describe the findings according to the adjusted regression results. We have also carefully reviewed the manuscript to ensure that the interpretation of age at first sexual intercourse is consistent throughout.

RESPONSE TO REVIEWER 1 (COMMENT 50)

Reviewer 1 – Comment 50:

Strengths and Limitations. Challenging to tease out actual reproductive transitions – how much of this data is associated with perinatal mental health conditions versus overall mental health among this population?

Author Response [Strengths and Limitations, Page 31, Line 680-684, page no: 32, line no: 704-713]

We sincerely thank the reviewer for this important observation. We agree that the present study evaluates mental health among women of reproductive age rather than specifically during the perinatal period. The MDHS 2022–2023 is a cross-sectional survey of women aged 15–49 years and does not permit differentiation of perinatal mental health conditions from overall anxiety and depression across the reproductive life course. To acknowledge this limitation, we have revised the Strengths and Limitations section to explicitly state that our findings reflect overall mental health among women of reproductive age and should not be interpreted as estimates of perinatal mental health disorders.

RESPONSE TO REVIEWER 1 (COMMENT 51)

Reviewer 1 – Comment 51:

Line 393, suggest changing "introducing" to "integrating".

Author Response [Implications, Page 33, Line 723]

We sincerely thank the reviewer for this suggestion. We agree that **"integrating"** more accurately reflects the incorporation of maternal mental health services into existing health systems. Accordingly, we have revised the wording throughout the manuscript.

RESPONSE TO REVIEWER 1 (COMMENT 52)

Reviewer 1 – Comment 52:

Again, clarify what reproductive transitions are.

Author Response [Methodology, Page 12-13, Line 265-268]

We thank the reviewer for this valuable suggestion. To improve clarity, we have explicitly defined **reproductive transitions** in the Methods section. In this study, the term refers to reproductive life-course milestones available in the MDHS dataset, including **age at first birth, age at first sexual intercourse, age at first cohabitation, pregnancy history, pregnancy loss, current pregnancy status, and parity-related characteristics.** We have also used this terminology consistently throughout the manuscript.

RESPONSE TO REVIEWER 1 (COMMENT 53)

Reviewer 1 – Comment 53:

Line 396, what about activities that improve gender equity and reduce IPV?

Author Response [Page 34, Line 752-759]

We sincerely thank the reviewer for this excellent recommendation. We agree that addressing maternal mental health requires not only improvements in healthcare services but also interventions targeting broader social determinants. Accordingly, we have expanded the Implications section to emphasize the importance of promoting women's decision-making autonomy, advancing gender equity, and implementing interventions that reduce acceptance and occurrence of intimate partner violence alongside strengthening mental health services.

RESPONSE TO REVIEWER 1 (COMMENT 54)

Reviewer 1 – Comment 54:

Unsure if equity-based is the most appropriate term here. Suggest a maternal health policy that integrated mental health from pre-pregnancy through two years postpartum.

Author Response [Conclusions, Page 34, Line 759-760]

We sincerely thank the reviewer for this thoughtful suggestion. Accordingly, we have revised the Conclusions to recommend integrating mental health services throughout the maternal health continuum, from pre-pregnancy to the postpartum period, while acknowledging the importance of strengthening supportive health systems.

RESPONSE TO REVIEWER 1 (COMMENT 55)

Reviewer 1 – Comment 55:

Line 404, and also tackles gender inequities that influence risk for mental health conditions.

Author Response [Conclusions, Page 34, Line 752-753]

We sincerely thank the reviewer for this valuable recommendation. Accordingly, we have revised the Conclusions to explicitly emphasize that maternal mental health policies should incorporate interventions that promote women's decision-making autonomy, reduce gender inequities, and address the acceptance of intimate partner violence.

Closing Statement

We sincerely thank **Reviewer 1** for the thorough, constructive, and insightful comments. The suggestions have substantially improved the clarity, scientific rigor, and overall quality of our manuscript. In response, we have carefully revised the manuscript by clarifying the study population and methodology, improving the presentation and interpretation of the results, strengthening the discussion and conclusions, refining the terminology throughout, and explicitly acknowledging the study limitations. We believe that these revisions have addressed all of the reviewer's concerns and have considerably strengthened the manuscript. We are grateful for the reviewer's valuable time and thoughtful feedback.

RESPONSE TO REVIEWER 2

RESPONSE TO REVIEWER 2 (GENERAL COMMENT)

Reviewer 2 – General Comment:

Needs a thorough copy edit; in many places sentences are not complete or not clear. For example line 217, "showed indicators of women's empowerment" is not clear. Line 284, Among rural women instead of In the rural women. Line 314, no capitalization on depression and anxiety. Lines 351, 352 need revision. And more.

Author Response [Throughout the Manuscript]

We sincerely thank the reviewer for this careful assessment and constructive feedback. We agree that improving the clarity and readability of the manuscript is essential. In response, we have thoroughly revised the entire manuscript to improve grammar, sentence structure, clarity, consistency of terminology, and overall readability. We carefully corrected the examples highlighted by the reviewer (including the wording related to women's decision-making autonomy, grammatical expressions such as "Among rural women," capitalization of anxiety and depression where appropriate, and the sentences identified on lines 351–352), as well as numerous additional language and stylistic issues throughout the manuscript. We believe these revisions have substantially improved the clarity, flow, and overall quality of the manuscript.

RESPONSE TO REVIEWER 2 (COMMENT 1)

Reviewer 2 – Comment 1:

"Objectives" lack references and rationale that link the covariates to the actual outcomes; while this is an observational study that cannot explore causal relationships, the first part of the paper lacks clear thought on how the risk factors can directly influence depression and anxiety. The pieces are better described in the discussion and conclusion, but need more thoughtful discussion in the motivation of the paper, the mechanisms and how they can relate to the findings. Water source and toilet type, for example, what is the framework for how these relate to depression and anxiety? The author talks about conceptual relevance last, but this should probably be first (line 130).

Author Response [Introduction, Page 6, Line 118-122, Methodology page no:11, line no: 235-274]

We sincerely thank the reviewer for this insightful suggestion. We agree that the conceptual rationale for selecting the study covariates should be presented more clearly before stating the study objectives. In response, we have strengthened the Introduction by explaining that the selected covariates were identified based on previous literature and a conceptual framework encompassing sociodemographic, reproductive, household, environmental, digital, and geographical determinants that may influence women's mental health through multiple pathways. We also clarified that household environmental factors, including water source and sanitation, were considered as indicators of living conditions and socioeconomic disadvantage that may contribute to psychological distress through chronic environmental stressors, poor living environments, and health-related vulnerabilities rather than implying causal relationships. Finally, we reordered the concluding paragraph of the Introduction so that the conceptual rationale precedes the study objectives and policy implications.

RESPONSE TO REVIEWER 2 (COMMENT 2)

Reviewer 2 – Comment 2:

Sometimes the author refers to ‘mental health’ instead of symptoms of depression or anxiety (line 224 for example). Those should be corrected. Line 238, unclear what is meant by preponderance? How would that influence higher symptom prevalence? Line 333, higher symptoms isn’t clear. Make sure to clearly distinguish where necessary if it is symptoms of anxiety, depression, or both. And what is the difference, would we expect both to move in the same direction with the covariates?

Author Response [Throughout the Manuscript]

We sincerely thank the reviewer for these careful observations. We agree that precise terminology is essential for accurately presenting the study findings. In response, we have thoroughly revised the manuscript to consistently distinguish between **anxiety symptoms**, **depressive symptoms**, and **both outcomes** where appropriate, replacing the broader term *mental health* when referring specifically to the study outcomes. We also clarified ambiguous wording, including replacing terms such as *preponderance* and *higher symptoms* with clearer and more precise descriptions. Throughout the Results and Discussion, we now explicitly indicate whether associations relate to anxiety symptoms, depressive symptoms, or both, and we have clarified that although several covariates were associated with both outcomes, the magnitude and statistical significance of these associations were not necessarily identical. These revisions have improved the clarity, precision, and consistency of the manuscript.

RESPONSE TO REVIEWER 2 (COMMENT 3)

Reviewer 2 – Comment 3:

If age at first sex is most salient, why? It is not just age at first sex, it is the younger a young woman is when she first has sex, the more likely she is to have anxiety and depression? Line 314.

Author Response [Page 25, Line 541-544, page no: 30, line no: 671-676]

We sincerely thank the reviewer for this important observation. We agree that our previous wording could be misinterpreted. In response, we have revised the manuscript to clarify that the association relates to **earlier age at first sexual intercourse** rather than age at first sex in general. We also avoid implying causality and instead describe this variable as one of the strongest correlates identified in the adjusted analyses. Throughout the Results and Discussion, we now explicitly distinguish women who initiated sexual intercourse before 18 years of age from those whose first sexual intercourse occurred at 18 years or older, thereby improving the interpretation of this finding.

RESPONSE TO REVIEWER 2 (COMMENT 4)

Reviewer 2 – Comment 4:

Line 319, the MDHS includes all women, not just ever married. If the sample is restricted this is not clear.

Author Response [Methodology, Page 10, Line 207-210]

We sincerely thank the reviewer for this helpful comment. We apologize for the lack of clarity. We have revised the Methods section to clearly describe the study population and sampling frame used in this analysis. Specifically, we now explicitly state that our analysis was based on the **Mozambique Demographic and Health Survey (MDHS) 2022–2023 women's dataset, which includes women aged 15–49 years selected through the DHS sampling design**, and we have revised the wording throughout the manuscript to ensure consistency regarding the study population.

Reviewer #2 Comment

I don't think there is any spatial analysis in this paper. The author looks at geographic differences, rural urban, etc., which are important but this is not the same as spatial analysis. I suggest changing "spatial" to "geographic" or "regional" analysis and keeping the focus on geographic comparisons which are in and of themselves interesting. In the figures, it is not spatial prevalence, it is simply regional prevalence.

Response

Thank you for this valuable observation. We agree that our analyses focused on **geographic (provincial) comparisons** rather than formal spatial statistical analyses. Accordingly, we have replaced the term **"spatial"** with **"geographic"** (or **"regional"**, where appropriate) throughout the manuscript, including the title, abstract, methods, results, discussion, conclusions, and figure captions. These revisions more accurately describe the analytical approach and avoid implying that formal spatial statistical methods were performed. We appreciate the reviewer for helping us improve the methodological accuracy and clarity of the manuscript.

Reviewer #2 Comment

I don't think the author clearly discusses the figures, and I'm not sure all of the figures are needed or particularly helpful to understand the findings. Not sure figure 1 adds much value unless there is a discussion of it and the differences observed. Figures 3 and 4 would be more useful if the covariates were ordered or grouped in some way and discussed in the results. Figure 7 could be useful for the beginning of the article. Maps are useful but could be more useful if discussed when looking at internal conflict. Color bins should be changed to quartiles or some other grouping that is easier to read. A map is not spatial prevalence.

Response

Thank you for these insightful and detailed comments regarding the presentation and interpretation of the figures. We have carefully revised the manuscript to improve clarity, structure, and the relevance of all visual elements.

First, we have strengthened the **integration of all figures within the Results section**, ensuring that each figure is explicitly described and interpreted in the text rather than being presented only as supplementary visuals. This includes adding clearer narrative explanations of the observed patterns and differences across subgroups.

Second, we evaluated the necessity of each figure and revised their placement and emphasis. **Figure 1 has been retained but now includes a clearer interpretation in the Results section highlighting key response patterns across GAD-7 and PHQ-9 items**, making its contribution to understanding symptom distribution more explicit.

Third, **Figures 3 and 4 have been revised by grouping and ordering covariates logically (e.g., sociodemographic, reproductive, household, and contextual factors)** to improve interpretability and allow clearer comparison across determinants. Corresponding updates were also made in the Results text to align with this structured presentation.

Fourth, we agree that **Figure 7 is conceptually important** and have repositioned it earlier in the manuscript to better support interpretation of the analytical framework and key relationships.

Fifth, for the geographic maps, we have revised both the **visual presentation and interpretation**. Specifically, we have adjusted the color classification to **quartile-based grouping** to improve readability and comparability across provinces. We have also updated the terminology throughout to avoid the phrase "spatial prevalence" and now consistently use **"provincial" or "geographic distribution"** instead.

Finally, we have revised the discussion of all figures in the Results section to ensure that each visualization is clearly explained, contextualized, and directly linked to the study findings, thereby improving coherence between text and figures.

We appreciate the reviewer's constructive feedback, which has significantly improved the clarity and presentation quality of the manuscript.

Reviewer Comment

It would be interesting to reflect on the use of the GAD7 and PHQ9 in this setting as a reliable and valid way to capture depression and anxiety in a nationally representative HH survey such as the DHS. In the comparison and existing literature, it should be noted if the studies the author is comparing were done using the GAD7 and PHQ9, same age groups, etc.

Response [Page no: 10, line no: 220-22, page no: 30, line no:664-668]

Thank you for this important suggestion. We agree that further contextualization of the GAD-7 and PHQ-9 is essential for interpreting findings within a nationally representative DHS framework. We have therefore expanded the Discussion to include a clearer justification of the use of these validated screening tools, noting their established reliability and validity for measuring symptoms of anxiety and depression in population-based surveys.

In addition, we have revised the **comparison with existing literature** to clarify whether referenced studies used the same instruments (GAD-7 and PHQ-9) and to specify differences in study populations, particularly age groups and sampling frames. Where applicable, we have highlighted methodological differences to improve comparability and interpretation of findings across studies.

Reviewer Comment

The discussion summarizes these patterns; this is the best part of the paper. Need to weave that more into the beginning and justification for why the analysis is being done. What is striking is how well the findings seem to hold up. It suggests that these scales are valid for use in Mozambique (and probably other countries) given the direction of the relationships found between the covariates and the outcomes. Need further discussion of Nampula, why does this region stand out? Why more so than Cabo Delgado?

Response [Page no: 28, line no:615-622]

Thank you for this highly constructive and encouraging feedback. We appreciate the recognition of the value of the findings and agree that the implications of the MDHS mental health module and the overall justification of the analysis should be more strongly emphasized earlier in the manuscript.

In response, we have revised the **Introduction and early Discussion** to more clearly highlight the importance of the newly available DHS mental health module and its potential to generate nationally

representative evidence on anxiety and depression in Mozambique. This has strengthened the rationale for conducting the analysis and better positioned the study within a broader evidence gap.

We also expanded the discussion on the **interpretive validity of the GAD-7 and PHQ-9 scales**, noting that the consistency and expected direction of associations across multiple sociodemographic, reproductive, and contextual covariates support their applicability in the Mozambican population context.

Regarding the geographic findings, we have further elaborated on the distinct burden observed in **Nampula province compared to other high-burden regions such as Cabo Delgado**. We now emphasize that Nampula may reflect a convergence of structural vulnerabilities, including higher poverty levels, lower health service accessibility, and a higher prevalence of early marriage and adolescent reproductive transitions, which may jointly contribute to increased mental health risk. In contrast, differences with Cabo Delgado are now more cautiously interpreted in relation to contextual heterogeneity, including potential variation in socioeconomic structure, service access, and recent regional dynamics.

These revisions improve the theoretical grounding of the study, strengthen the interpretation of key regional differences, and better integrate the significance of the MDHS mental health module throughout the manuscript.

Closing Statement

We sincerely thank the reviewer for the highly constructive and insightful comments, which have significantly strengthened the manuscript. The suggestions helped us improve clarity, methodological precision, and theoretical framing, particularly in strengthening the rationale for the study, improving interpretation of geographic differences, and better contextualizing the use of validated mental health screening tools within the MDHS framework. We believe the revised manuscript is now substantially clearer, more coherent, and better aligned with both methodological standards and the broader literature on maternal mental health in low-resource settings.
