# Supplementary material for: Stratified and geographic patterns of depression and anxiety in Mozambican women: Mental health policy implications
Source: PLoS One. 2026 Aug 19;21(8):e0356265. doi: 10.1371/journal.pone.0356265 (PMC13489479; doi:10.1371/journal.pone.0356265)
Supplement: Supplementary file 2 [file pone.0356265.s005.pdf]

Date: 27 July 2026

Dear Dr. Jennifer Yourkavitch
Academic Editor
PLOS ONE

Re: Revised Manuscript Submission (Minor Revision)

Manuscript ID: PONE-D-25-61419R1

Thank you for the opportunity to revise and resubmit our manuscript entitled:

"Stratified and Geographic Patterns of Depression and Anxiety in Mozambican Women: Mental Health Policy Implications."

We sincerely appreciate your careful evaluation of our manuscript and your constructive comments. Your thoughtful suggestions have helped us further improve the clarity, scientific rigor, and policy relevance of the manuscript.

In response to your comments, we have carefully revised the manuscript and addressed all points in detail in the accompanying document entitled **"Response to Reviewers,"** where each comment is followed by our point-by-point response and a description of the corresponding revisions made in the manuscript.

We have also submitted:

- A revised manuscript with tracked changes.
- A clean version of the revised manuscript.

The principal revisions include:

- Revised the **Abstract** and **main Conclusions** to ensure that the conclusions are directly supported by the study findings and provide more specific, evidence-based public health implications.
- Reduced repetition of results in the Conclusion and strengthened the interpretation and policy relevance of the findings.
- Improved terminology consistency throughout the manuscript, including the consistent use of **"anxiety symptoms"** and **"depression symptoms."**
- Defined the **Strengthening the Reporting of Observational Studies in Epidemiology (STROBE)** guideline at its first mention and improved reporting consistency throughout the manuscript.
- Performed additional language editing to improve clarity, grammar, readability, and scientific presentation.

- Carefully reviewed and updated the reference list, including the addition of relevant recent references where appropriate, and confirmed that no retracted publications are cited.
- Enhanced transparency and reproducibility by making the R scripts used for data management, statistical analyses, and figure generation publicly available in a GitHub repository, together with documentation describing the analytical workflow: **https://github.com/muhammadsalek/mozambique-mental-health-analysis**.

We have also ensured that the revised manuscript complies with the PLOS ONE reporting and formatting requirements.

We believe that these revisions have fully addressed the Academic Editor's comments and have further strengthened the scientific quality, clarity, and policy relevance of the manuscript.

Thank you once again for your valuable time, thoughtful guidance, and the opportunity to improve our work. We hope that the revised manuscript is now suitable for publication in **PLOS ONE**, and we look forward to your favorable consideration.

Sincerely,

Md Salek Miah
Corresponding Author
Department of Statistics
Shahjalal University of Science & Technology (SUST)
Sylhet-3114, Bangladesh

Email: saleksta@gmail.com
ORCID: 0009-0005-5973-461X

Journal Requirements:

If the reviewer comments include a recommendation to cite specific previously published works, please review and evaluate these publications to determine whether they are relevant and should be cited. There is no requirement to cite these works unless the editor has indicated otherwise.

Please review your reference list to ensure that it is complete and correct. If you have cited papers that have been retracted, please include the rationale for doing so in the manuscript text, or remove these references and replace them with relevant current references. Any changes to the reference list should be mentioned in the rebuttal letter that accompanies your revised manuscript. If you need to cite a retracted article, indicate the article's retracted status in the References list and also include a citation and full reference for the retraction notice.

Response: [Through manuscripts]

Thank you for these important editorial requirements. We carefully reviewed the reference list to ensure that all citations are accurate, complete, and up to date. We also evaluated all references for relevance and confirmed that no retracted publications are cited in the revised manuscript. Where appropriate, we updated and added recent relevant references to strengthen the manuscript. No reviewer-suggested references were added unless they were considered directly relevant to the study.

Academic Editor Comment:

The conclusion in the Abstract comprises general statements that do not reflect your findings. The conclusion section in the main narrative relies too heavily on restating results. Please revise the conclusions sections in the Abstract and in the narrative so that they better reflect the study findings and their implications.

Response: [Page no: 3, line no:52-57, page no:21, line no:577-600]

We sincerely thank the Academic Editor for this valuable suggestion. We have revised both the Abstract and the main Conclusion sections to ensure that the conclusions are more closely aligned with the principal findings of the study. Specifically, we replaced the previous general statements with findings-based conclusions by highlighting the need to prioritize women aged 35–49 years, strengthen women's decision-making autonomy, and focus interventions in high-burden provinces. We also revised the policy implications to better reflect the evidence generated by this study rather than restating the results.

Additional Revisions [Through manuscripts and page no:10, line no:272-275]

In addition to addressing the Academic Editor's comment, we made several editorial and reporting improvements throughout the manuscript to enhance clarity, consistency, and reproducibility. Specifically:

- We revised terminology throughout the manuscript to consistently use **"anxiety symptoms"** and **"depression symptoms"** where appropriate, rather than referring broadly to mental health outcomes.
- We defined the **Strengthening the Reporting of Observational Studies in Epidemiology (STROBE)** guideline in full at its first mention and ensured consistent reporting throughout the manuscript.
- We performed additional language editing to improve clarity, grammar, consistency, and scientific readability.

- To further support transparency and reproducibility, we have made the analysis scripts and documentation publicly available in a GitHub repository, which is cited in the manuscript's reproducibility statement and is available at: **https://github.com/muhammadsalek/mozambique-mental-health-analysis**.

Figure Requirements

To ensure your figures meet our technical requirements, please review our figure guidelines: https://journals.plos.org/plosone/s/figures

You may also use PLOS's free figure tool, NAAS, to help you prepare publication quality figures: https://journals.plos.org/plosone/s/figures#loc-tools-for-figure-preparation.

NAAS will assess whether your figures meet our technical requirements by comparing each figure against our figure specifications.

Response:

- Thank you for these recommendations. We carefully reviewed the PLOS ONE figure preparation guidelines and revised the figures where necessary to improve quality, clarity, labeling, and overall presentation. We also verified that all figures comply with the journal's technical requirements. To further enhance transparency and reproducibility, we have made the R scripts used for figure generation and statistical analyses publicly available in our GitHub repository, together with documentation describing the analytical workflow: **https://github.com/muhammadsalek/mozambique-mental-health-analysis**.
