# Supplementary material for: Stratified and geographic patterns of depression and anxiety in Mozambican women: Mental health policy implications
Source: PLoS One. 2026 Aug 19;21(8):e0356265. doi: 10.1371/journal.pone.0356265 (PMC13489479; doi:10.1371/journal.pone.0356265)
Supplement: Supplementary file 3 [file pone.0356265.s001.docx]

**Supplementary**

Table S1: Frequency distributions for GAD-7 and PHQ-9 items (n=13,183).

| Scale | Items | Never | Rarely | Often | Always | Don’t know | Refused |
| --- | --- | --- | --- | --- | --- | --- | --- |
|  |  | n (%) | n (%) | n (%) | n (%) | n (%) | n (%) |
| Over the last two weeks, how often have you been bothered by the following problems? | | | | | |  |  |
| GAD-7 items | 1. Feeling nervous, anxious or on edge | 7915 (60) | 3663 (27.8) | 1149 (8.7) | 369 (2.8) | 60 (0.5) | 26 (0.2) |
|  | 2. Not being able to stop or control worrying | 8768 (66.5) | 2970 (22.5) | 1102 (8.4) | 275 (2.1) | 53 (0.4) | 15 (0.1) |
|  | 3. Worrying too much about different things | 7779 (59) | 3616 (27.4) | 1249 (9.5) | 497 (3.8) | 35 (0.3) | 7 (0.1) |
|  | 4. Trouble relaxing | 8523 (64.7) | 3156 (23.9) | 1102 (8.4) | 345 (2.6) | 47 (0.4) | 10 (0.1) |
|  | 5. Being so restless that it is hard to sit still | 8945 (67.9) | 2904 (22) | 998 (7.6) | 289 (2.2) | 38 (0.3) | 9 (0.1) |
|  | 6. Becoming easily annoyed or irritated | 7773 (59) | 3660 (27.8) | 1297 (9.8) | 415 (3.2) | 30 (0.2) | 6 (0) |
|  | 7. Feeling afraid as if something awful might happen | 8700 (66) | 3040 (23.1) | 1062 (8.1) | 336 (2.6) | 34 (0.3) | 10 (0.1) |
| PHQ-9 items | 1. Little interest or pleasure in doing things |  |  |  |  |  |  |
|  | 2. Feeling down, depressed, or hopeless | 8974 (68.1) | 2919 (22.1) | 948 (7.2) | 310 (2.4) | 27 (0.2) | 5 (0) |
|  | 3. Trouble falling or staying asleep, or sleeping too much | 8310 (63) | 3425 (26) | 1076 (8.2) | 328 (2.5) | 31 (0.2) | 13 (0.1) |
|  | 4. Feeling tired or having little energy | 8484 (64.4) | 3150 (23.9) | 1165 (8.8) | 347 (2.6) | 24 (0.2) | 14 (0.1) |
|  | 5. Poor appetite or overeating | 8648 (65.6) | 3135 (23.8) | 1088 (8.3) | 289 (2.2) | 17 (0.1) | 6 (0) |
|  | 6. Feeling bad about yourself - or that you are a failure or have let yourself or your family down | 9088 (68.9) | 2828 (21.5) | 937 (7.1) | 304 (2.3) | 18 (0.1) | 8 (0.1) |
|  | 7. Trouble concentrating on things, such as reading the newspaper or watching television | 10339 (78.4) | 1939 (14.7) | 690 (5.2) | 176 (1.3) | 26 (0.2) | 13 (0.1) |
|  | 8. Moving or speaking so slowly that other people could have noticed? Or the opposite - being so fidgety or restless that you have been moving around a lot more than usual | 10161 (77.1) | 2168 (16.4) | 656 (5) | 165 (1.3) | 30 (0.2) | 4 (0) |
|  | 9. Thoughts that you would be better off dead or of hurting yourself in some way | 10504 (79.7) | 1898 (14.4) | 605 (4.6) | 139 (1.1) | 31 (0.2) | 7 (0.1) |
