# Supplementary material for: Stratified and geographic patterns of depression and anxiety in Mozambican women: Mental health policy implications
Source: PLoS One. 2026 Aug 19;21(8):e0356265. doi: 10.1371/journal.pone.0356265 (PMC13489479; doi:10.1371/journal.pone.0356265)
Supplement: Peer review history

# Peer review: Decision Letter 0

**DOI:** 10.1371/journal.pone.0356265.r001

**Authors:** Jennifer Yourkavitch

4 May 2026

PONE-D-25-61419

Stratified and Spatial Patterns of Depression and Anxiety in Mozambican Women: Maternal Health Policy Implications

PLOS One

Dear Dr. Miah,

Thank you for submitting your manuscript to PLOS ONE. After careful consideration, we feel that it has merit but does not fully meet PLOS ONE’s publication criteria as it currently stands. Therefore, we invite you to submit a revised version of the manuscript that addresses the points raised during the review process.

Thank you for this important work. The reviewers agree you have important information to share but have suggested many areas for revision. Please respond to each suggestion by the reviewers.

Please submit your revised manuscript by Jun 18 2026 11:59PM. If you will need more time than this to complete your revisions, please reply to this message or contact the journal office at plosone@plos.org. When you're ready to submit your revision, log on to https://www.editorialmanager.com/pone/ and select the 'Submissions Needing Revision' folder to locate your manuscript file.

Please include the following items when submitting your revised manuscript:

- A letter that responds to each point raised by the academic editor and reviewer(s). You should upload this letter as a separate file labeled 'Response to Reviewers'.
- A marked-up copy of your manuscript that highlights changes made to the original version. You should upload this as a separate file labeled 'Revised Manuscript with Track Changes'.
- An unmarked version of your revised paper without tracked changes. You should upload this as a separate file labeled 'Manuscript'.

If you would like to make changes to your financial disclosure, please include your updated statement in your cover letter. Guidelines for resubmitting your figure files are available below the reviewer comments at the end of this letter.

If applicable, we recommend that you deposit your laboratory protocols in protocols.io to enhance the reproducibility of your results. Protocols.io assigns your protocol its own identifier (DOI) so that it can be cited independently in the future. For instructions see: https://journals.plos.org/plosone/s/submission-guidelines#loc-laboratory-protocols. Additionally, PLOS ONE offers an option for publishing peer-reviewed Lab Protocol articles, which describe protocols hosted on protocols.io. Read more information on sharing protocols at https://plos.org/protocols?utm_medium=editorial-email&utm_source=authorletters&utm_campaign=protocols.

As the corresponding author, your ORCID iD is verified in the submission system and will appear in the published article. PLOS supports the use of ORCID, and we encourage all coauthors to register for an ORCID iD and use it as well. Please encourage your coauthors to verify their ORCID iD within the submission system before final acceptance, as unverified ORCID iDs will not appear in the published article. *Only*  the individual author can complete the verification step; PLOS staff *cannot*  verify ORCID iDs on behalf of authors.

We look forward to receiving your revised manuscript.

Kind regards,

Jennifer Yourkavitch

Academic Editor

PLOS One

Journal Requirements:

When submitting your revision, we need you to address these additional requirements.

1. Please ensure that your manuscript meets PLOS ONE's style requirements, including those for file naming. The PLOS ONE style templates can be found at https://journals.plos.org/plosone/s/file?id=wjVg/PLOSOne_formatting_sample_main_body.pdf and https://journals.plos.org/plosone/s/file?id=ba62/PLOSOne_formatting_sample_title_authors_affiliations.pdf

2. In the online submission form, you indicated that [The data underlying the results presented in this study are publicly available from the Demographic and Health Surveys (DHS) Program, accessible at https://dhsprogram.com/data/available-datasets.cfm Researchers must register and request access to the Mozambique 2022–2023 DHS dataset to use these data. All analyses reported in this manuscript were conducted using the de-identified data provided by DHS.].

All PLOS journals now require all data underlying the findings described in their manuscript to be freely available to other researchers, either 1. In a public repository, 2. Within the manuscript itself, or 3. Uploaded as supplementary information.

This policy applies to all data except where public deposition would breach compliance with the protocol approved by your research ethics board. If your data cannot be made publicly available for ethical or legal reasons (e.g., public availability would compromise patient privacy), please explain your reasons on resubmission and your exemption request will be escalated for approval.

3. We note that Figures 5, 6, and S1 in your submission contain map images which may be copyrighted. All PLOS content is published under the Creative Commons Attribution License (CC BY 4.0), which means that the manuscript, images, and Supporting Information files will be freely available online, and any third party is permitted to access, download, copy, distribute, and use these materials in any way, even commercially, with proper attribution. For these reasons, we cannot publish previously copyrighted maps or satellite images created using proprietary data, such as Google software (Google Maps, Street View, and Earth). For more information, see our copyright guidelines: http://journals.plos.org/plosone/s/licenses-and-copyright.

We require you to either (1) present written permission from the copyright holder to publish these figures specifically under the CC BY 4.0 license, or (2) remove the figures from your submission:

1. You may seek permission from the original copyright holder of Figures 5, 6, and S1 to publish the content specifically under the CC BY 4.0 license.

We recommend that you contact the original copyright holder with the Content Permission Form (http://journals.plos.org/plosone/s/file?id=7c09/content-permission-form.pdf) and the following text:

“I request permission for the open-access journal PLOS ONE to publish XXX under the Creative Commons Attribution License (CCAL) CC BY 4.0 (http://creativecommons.org/licenses/by/4.0/). Please be aware that this license allows unrestricted use and distribution, even commercially, by third parties. Please reply and provide explicit written permission to publish XXX under a CC BY license and complete the attached form.”

Please upload the completed Content Permission Form or other proof of granted permissions as an "Other" file with your submission.

In the figure caption of the copyrighted figure, please include the following text: “Reprinted from [ref] under a CC BY license, with permission from [name of publisher], original copyright [original copyright year].”

2. If you are unable to obtain permission from the original copyright holder to publish these figures under the CC BY 4.0 license or if the copyright holder’s requirements are incompatible with the CC BY 4.0 license, please either i) remove the figure or ii) supply a replacement figure that complies with the CC BY 4.0 license. Please check copyright information on all replacement figures and update the figure caption with source information. If applicable, please specify in the figure caption text when a figure is similar but not identical to the original image and is therefore for illustrative purposes only.

The following resources for replacing copyrighted map figures may be helpful:

USGS National Map Viewer (public domain): http://viewer.nationalmap.gov/viewer/

The Gateway to Astronaut Photography of Earth (public domain): http://eol.jsc.nasa.gov/sseop/clickmap/

Maps at the CIA (public domain): https://www.cia.gov/library/publications/the-world-factbook/index.html and https://www.cia.gov/library/publications/cia-maps-publications/index.html

NASA Earth Observatory (public domain): http://earthobservatory.nasa.gov/

Landsat: http://landsat.visibleearth.nasa.gov/

USGS EROS (Earth Resources Observatory and Science (EROS) Center) (public domain): http://eros.usgs.gov/#

Natural Earth (public domain): http://www.naturalearthdata.com/

4. Please include captions for your Supporting Information files at the end of your manuscript, and update any in-text citations to match accordingly. Please see our Supporting Information guidelines for more information: http://journals.plos.org/plosone/s/supporting-information.

If the reviewer comments include a recommendation to cite specific previously published works, please review and evaluate these publications to determine whether they are relevant and should be cited. There is no requirement to cite these works unless the editor has indicated otherwise.

[Note: HTML markup is below. Please do not edit.]

Reviewers' comments:

Reviewer's Responses to Questions

**Comments to the Author**

1. Is the manuscript technically sound, and do the data support the conclusions?

The manuscript must describe a technically sound piece of scientific research with data that supports the conclusions. Experiments must have been conducted rigorously, with appropriate controls, replication, and sample sizes. The conclusions must be drawn appropriately based on the data presented.

Reviewer #1: Partly

Reviewer #2: Partly

**********

2. Has the statistical analysis been performed appropriately and rigorously?

Reviewer #1: I Don't Know

Reviewer #2: Yes

**********

3. Have the authors made all data underlying the findings in their manuscript fully available?

The PLOS Data policy requires authors to make all data underlying the findings described in their manuscript fully available without restriction, with rare exception (please refer to the Data Availability Statement in the manuscript PDF file). The data should be provided as part of the manuscript or its supporting information, or deposited to a public repository. For example, in addition to summary statistics, the data points behind means, medians and variance measures should be available. If there are restrictions on publicly sharing data—e.g. participant privacy or use of data from a third party—those must be specified.

Reviewer #1: Yes

Reviewer #2: Yes

**********

4. Is the manuscript presented in an intelligible fashion and written in standard English?

PLOS ONE does not copyedit accepted manuscripts, so the language in submitted articles must be clear, correct, and unambiguous. Any typographical or grammatical errors should be corrected at revision, so please note any specific errors here.

Reviewer #1: No

Reviewer #2: No

**********

5. Review Comments to the Author

Please use the space provided to explain your answers to the questions above. You may also include additional comments for the author, including concerns about dual publication, research ethics, or publication ethics. (Please upload your review as an attachment if it exceeds 20,000 characters)

Reviewer #1: Thank you for the opportunity to review this manuscript. Overall this is an interesting approach to examine mental health prevalence among girls and women among reproductive age and its clear that the authors have put a great deal of work into the analysis. While I believe that the study findings may be useful, there is opportunity for clarification so that the readers can understand both the study methodology and the results. Overall, the title of the paper leads the reader to understand that this is a study specifically examining maternal mental health – but it reads as a study that examined mental health among girls and women among reproductive age, but its difficult to tease out which results specifically refer to perinatal mental health versus girls’ and womens’ mental health in general.

The manuscript will greatly benefit from much more detail within the methodology section that extends beyond the analysis used, and greater organization including sub-headings within the results as its currently quite confusing and as it currently reads, many of the results appear to be contradictory. An overall copy edit for language will also be helpful.

Thank you again for the opportunity to review and congratulations on all the work that you have done thus far.

Abstract:

Methodology:

1. It will be helpful to have age disaggregation bands available here as otherwise it is challenging to interpret the findings.

2. Also, it is clear that the DHS was used to gather PHQ-9 and GAD-7 data, were additional measures also gathered from the DHS? If yes, please specify or please include mention of other scales employed by the study team such as the scale for women’s empowerment, HH asset ownership, how internet usage was assessed.

Findings.

3. 11% anxiety and 10% depressive. It would be useful to understand the percentage comorbid depression and anxiety.

4. In your findings for the age-bands, it will be helpful to specify which group had the highest odds of anxiety, and then next highest.

Conclusions:

5. Suggest extending the conclusion beyond integrated policies. While this is an important start, without supportive health systems, skilled clinicians, and available services, the policies will not address MMH challenges experienced by Mozambican girls and women.

Introduction.

6. Suggest ending the first sentence on line 67 after disease and starting a new sentence with “In 2021…”

7. Line 69…should be “worsen” and “elevate the risk of comborbid…

8. Just a note that if you are including girls as young as 15 years old, so I would encourage the authors to refer to the study population as “girls and women of reproductive age”.

9. line 73: Depression and anxiety disproportionately affect girls and women in LMIC…

10. line 76: Would encourage including lack of supportive policies and guidelines, mental health stigma, lack of specialized services as other issues.

11. If the authors are going to discuss internet access in the introduction, also suggest drawing in specific examples from the literature in regards to MMH specifically, otherwise this doesn’t seem a natural fit within this section.

12. Note that lines 81-83 state that there is little information on mental health twice. Also be sure throughout to note that the topic is specifically maternal mental health which is even further neglected area of mental health.

13. I have yet to see information on the influence of access to media on MMH, it would be helpful to include that specific relationship on lines 90-91.

14. Suggest ending the introduction with a sentence or two on how this methodological framework may be useful for other countries interested in developing responsive and data-driven MMH policies.

Methodology.

15. Line 142, what does the two areas missed refer to?

16. To confirm, this is mental health in general among women of reproductive age? These women have not necessarily had a pregnancy?

17. How was covariate data gathered? Was this all available in the DHS? If not, please specify tools used and how data was gathered if means were required outside of standard DHS. Beyond the statistical analysis, much more information is needed on how all of these measures were collected.

18. Starting on line 174. I am curious as to whether HIV status and/or migration status was also considered as variables given these are also widely recognized risk factors?

Results.

19. Line 215. Please specify what higher education refers to.

20. Line 2016. Please clarify what “justified intimate partner violence refers to”. Did they believe that 2/3rds of women who experienced IPV thought that it was excusable? Or among women in general? More specificity here will be important to understand the statement.

21. Line 217, What are indicators of women’s empowerment (it would help if the information were available in the methods).

22. Line 218. What does improved sanitation refer to? Running water? Toilet in the house?

23. For lines 217-222, in the methodology it will be important to have the various scales that were used to understand how to interpret all of these results.

24. What was the % of comorbid anxiety and depression? If this not possible to ascertain from the DHS? If not, please include in the limitations.

25. The abstract says that women in their mid 30s and then again in their mid 40s experienced the highest odds of mental health symptoms, but lines 224-225 say that prevalence of symptoms was highest among young people. More clarification is needed to understand this conflicting data.

26. Line 226. Please clarify what ‘early childbirth’ refers to. Assume this means younger age when gave birth, and not pre-term birth?

27. Also, early childbirth (if referring to young age), early sexual debut and early cohabitation all represent a similar phenomenon – just noting that these are similar and wondering if this should be teased out any further to understand specifically if its early motherhood (adolescents are among the highest risk for a perinatal mental health disorder), or if simply cohabitating places a girl at risk.

28. Line 230. Please clarify the similar trends across partners’ education, etc. Does that mean that girls and women experienced more mental health symptoms based upon their partners education?

29. Line 234, a new paragraph should start with “Among the…

30. Line 236, aren’t three or more children and multiple pregnancies the same? Or does multiple pregnancies refer to twins and triplets?

31. Line 236, what does inadequate ANC visits refer to? Please include a number – e.g., did not meet the threshold # of…

32. Line 236-237, pressure to become pregnant from who?

33. Line 237, the sentence about previous pregnancy loss is confusing. It currently reads that women who had NOT experienced a previous loss were more likely to experience depression and anxiety. This is a surprising finding given that previous pregnancy loss and stillbirth are risk factors for perinatal mental health disorders. More clarification is needed.

34. Women abstaining from sex?

35. Please name the specific religious grouping under study.

36. Line 244, had more than twice the odds in comparison to which other age group? Again, these age groupings are confusing with the information reported in the abstract. As it reads right now, it looks as though the researchers have lumped girls and women 15-39 together to indicate twice the odds of anxiety (in comparison to who?), and higher odds of depression, and then also states that women 45-49 also have higher odds of anxiety. Again, across all of these, what is the reference or comparison group to whom the higher odds are being compared to?

37. Line 255. Internet use is stated to report higher risk of anxiety, but line 233 says that symptoms were higher among those without internet access. This seems contradictory?

38. Line 262. These findings of higher odds of anxiety correlate to what was reported in the abstract. But how is this different from the findings of anxiety reported among girls? Are there sub-headings missing by location? The results as written are quite confusing.

39. Line 279, it appears that a bracket is missing.

40. Line 287, am I understanding correctly that this data is only rural women now, and early sexual debut was protective against experiencing anxiety? I would use caution with that terminology as its not clear that having sex at a young age is protective, but rather girls who had sex at a young age in rural areas experienced lower odds of depression in comparison to who?

41. Line 293, the reference to toilet facilities is unclear.

42. Line 311. What does household division refer to?

43. Early sexual debut now appears to be the greatest risk factor, but earlier was said to be protective in rural areas?

44. Line 315-316. Unclear what AUC and ROC refers to and what it means in the context of the results. What should the reader infer from this?

45. Overall comment on the results section. It is quite long and confusing. Sub-headings are needed and a great deal of attention to clarify the different analysis which seem to currently overlap and contradict each other. There are also several spaces where the authors use “higher odds” but don’t provide a reference/comparison group.

Discussion

46. Line 322. A definition for ‘reproductive transitions’ should be included within the methodology. It will be important to understand how much of this data is actually centered around the reproductive transitions once the reader has an idea what that transition refers to.

47. Line 332. This is the first reference that I’ve read regarding poor quality care. How was this ascertained? There was earlier mention of fewer ANC visits, but this seems different.

48. I am not convinced that the internet has any meaningful impact based upon this study, it seems like a superficial measure with perhaps some correlations but not necessarily impacts – if the authors are to include internet within this study, suggest further explanation and justifications for its inclusion. Especially given internet was not necessarily widely accessible.

49. The authors indicate that early sexual debut is the main risk factor but than state here that women over age 18 were more likely to have symptoms.

Strengths and Limitations

50. Challenging to tease out actual reproductive transitions – how much of this data is associated with perinatal mental health conditions versus overall mental health among this population?

Implications

51. Line 393, suggest changing ‘introducing” to “integrating”.

52. Again, clarify what reproductive transitions are.

53. Line 396, what about activities that improve gender equity and reduce IPV?

Conclusions

54. Unsure if equity-based is the most appropriate term here. Suggest a maternal health policy that integrated mental health from pre-pregnancy through two years postpartum.

55. Line 404, and also tackles gender inequities that influence risk for mental health conditions.

Reviewer #2: Needs a thorough copy edit; in many places sentences are not complete or not clear. For example line 217, “showed indicators of women’s empowerment” is not clear. Line 284, Among rural women instead of In the rural women. Line 314, no capitalization on depression and anxiety. Lines 351, 352 need revision. And more.

"Objectives" lack references and rationale that link the covariates to the actual outcomes; while this is an observational study that cannot explore causal relationships, the first part of the paper lacks clear thought on how the risk factors can directly influence depression and anxiety. The pieces are better described in the discussion and conclusion, but need more thoughtful discussion in the motivation of the paper, the mechanisms and how they can relate to the findings. Water source and toilet type, for example, what is the framework for how these relate to depression and anxiety? The author talks about conceptual relevance last, but this should probably be first (line 130).

Sometimes the author refers to ‘mental health’ instead of symptoms of depression or anxiety (line 224 for example). Those should be corrected. Line 238, unclear what is meant by preponderance? How would that influence higher symptom prevalence? Line 333, higher symptoms isn’t clear. Make sure to clearly distinguish where necessary if it is symptoms of anxiety, depression, or both. And what is the difference, would we expect both to move in the same direction with the covariates?

If age at first sex is most salient, why? It is not just age at first sex, it is the younger a young woman is when she first has sex, the more likely she is to have anxiety and depression? Line 314. Line 319, the MDHS includes all women, not just ever married. If the sample is restricted this is not clear.

I don’t think there is any spatial analysis in this paper. The author looks at geographic differences, rural urban, etc, which are important but this is not the same as spatial analysis. I suggest changing “spatial” to “geographic” or “regional” analysis and keeping the focus on geographic comparisons which are in and of themselves interesting. In the figures, it is not spatial prevalence, it is simply regional prevalence.

I don't think the author clearly discusses the figures, and I'm not sure all of the figures are needed or particularly helpful to understand the findings. Not sure figure 1 adds much value unless there is a discussion of it and the differences observed. Figures 3 and 4 would be more useful if the covariates were ordered or grouped in some way and discussed in the results. Figure 7 could be useful for the beginning of the article. Maps are useful but could be more useful if discussed when looking at internal conflict. Color bins should be changed to quartiles or some other grouping that is easier to read. A map is not spatial prevalence.

It would be interesting to reflect on the use of the GAD7 and PHQ9 in this setting as a reliable and valid way to capture depression and anxiety in a nationally representative HH survey such as the DHS.

In the Comparison and Existing Literature, it should be noted if the studies the author is comparing were done using the GAD7 and PHQ9, same age groups, etc?

That said, the analysis is an important contribution to the literature as this is the first time the DHS has included the mental health module in any survey. The discussion summarizes these patterns; this is the best part of the paper. Need to weave that more into the beginning and justification for why the analysis is being done. What is striking is how well the findings seem to hold up. It suggests that these scales are valid for use in Mozambique (and probably other countries) given the direction of the relationships found between the covariates and the outcomes. Need further discussion of Nampula, why does this region stand out? Why more so than Cabo Delgado?

**********

6. PLOS authors have the option to publish the peer review history of their article (what does this mean?). If published, this will include your full peer review and any attached files.

If you choose “no”, your identity will remain anonymous but your review may still be made public.

**Do you want your identity to be public for this peer review?**  For information about this choice, including consent withdrawal, please see our Privacy Policy.

Reviewer #1: No

Reviewer #2: No

**********

[NOTE: If reviewer comments were submitted as an attachment file, they will be attached to this email and accessible via the submission site. Please log into your account, locate the manuscript record, and check for the action link "View Attachments". If this link does not appear, there are no attachment files.]

To ensure your figures meet our technical requirements, please review our figure guidelines: https://journals.plos.org/plosone/s/figures

You may also use PLOS’s free figure tool, NAAS, to help you prepare publication quality figures: https://journals.plos.org/plosone/s/figures#loc-tools-for-figure-preparation.

NAAS will assess whether your figures meet our technical requirements by comparing each figure against our figure specifications.

# Author comment: Author response to Decision Letter 1

**DOI:** 10.1371/journal.pone.0356265.r002

3 Jul 2026

Date: July 3, 2026

Dear Dr. Jennifer Yourkavitch

Academic Editor

PLOS ONE

Re: Revised Manuscript Submission

Thank you for the opportunity to revise and resubmit our manuscript entitled:

“Stratified and Geographic Patterns of Depression and Anxiety in Mozambican Women: Maternal Mental Health Policy Implications”

We sincerely appreciate the time and constructive feedback provided by the Editor and reviewers, which has significantly improved the quality and clarity of the manuscript.

In response to the comments, we have carefully revised the manuscript and addressed all issues in detail in a separate document titled “Response to Reviewers,” where each comment is reproduced followed by our point-by-point response and corresponding changes in the manuscript.

We have also submitted:

• A revised manuscript with tracked changes

• A clean final version of the manuscript

• Updated figures and tables, where applicable

Key revisions include:

• Improved terminology consistency throughout the manuscript

• Revised Results section for clearer structure and analytical presentation

• Reframed “spatial analysis” as “geographic/provincial analysis” for methodological accuracy

• Improved figure presentation and interpretation

• Updated maps using quartile-based classification

• Clarified reference groups and model specifications

• Strengthened discussion and theoretical framing

• Enhanced methodological clarity and variable definitions

• Improved limitations section

• Standardized statistical reporting

• Strengthened conclusions and policy relevance

We have also ensured full compliance with PLOS ONE formatting and submission requirements.

Additional notes

We would like to inform you that the corresponding author’s email address has been updated, and additional relevant references have been included in the revised manuscript.

We also apologize for the delayed submission. The revised manuscript was originally planned for submission on June 25, 2026; however, due to an unexpected electricity outage, we were unable to complete the submission on time. We are submitting it at the earliest possible opportunity on June 26, 2026.

We hope the revised manuscript adequately addresses all concerns and is now suitable for publication in PLOS ONE. We greatly appreciate your consideration and the opportunity to improve our work.

Sincerely,

Md Salek Miah

Corresponding Author

Department of Statistics

Shahjalal University of Science & Technology (SUST), Bangladesh

Email: saleksta@gmail.com

ORCID: 0009-0005-5973-461X

Requirement 1: PLOS ONE formatting and file naming

Editor Comment:

Please ensure that your manuscript meets PLOS ONE's style requirements, including those for file naming. The PLOS ONE style templates can be found at

https://journals.plos.org/plosone/s/file?id=wjVg/PLOSOne_formatting_sample_main_body.pdf and

https://journals.plos.org/plosone/s/file?id=ba62/PLOSOne_formatting_sample_title_authors_affiliations.pdf

Response:

Thank you for this comment. We have carefully reviewed the PLOS ONE formatting guidelines using the official templates provided by the journal. Based on these requirements, we have thoroughly revised the manuscript to ensure full compliance with PLOS ONE style.

Specifically, we have:

• Reformatted the manuscript according to PLOS ONE structural guidelines

• Revised title page, author names, and institutional affiliations to match journal requirements

• Standardized headings, subheadings, tables, and references

• Corrected and updated file naming according to submission system requirements

• Ensured consistency in formatting throughout the manuscript

These revisions ensure full adherence to PLOS ONE formatting and file naming standards.

Requirement 2: Data availability statement and DHS dataset policy

Editor Comment:

In the online submission form, you indicated that [The data underlying the results presented in this study are publicly available from the Demographic and Health Surveys (DHS) Program, accessible at https://dhsprogram.com/data/available-datasets.cfm Researchers must register and request access to the Mozambique 2022–2023 DHS dataset to use these data. All analyses reported in this manuscript were conducted using the de-identified data provided by DHS.].

All PLOS journals now require all data underlying the findings described in their manuscript to be freely available to other researchers, either 1. In a public repository, 2. Within the manuscript itself, or 3. Uploaded as supplementary information.

This policy applies to all data except where public deposition would breach compliance with the protocol approved by your research ethics board. If your data cannot be made publicly available for ethical or legal reasons (e.g., public availability would compromise patient privacy), please explain your reasons on resubmission and your exemption request will be escalated for approval.

Response:

Thank you for this important clarification regarding PLOS ONE data availability policy. We confirm that the data used in this study are derived from the Mozambique 2022–2023 Demographic and Health Survey (DHS) dataset, which is publicly accessible upon registration and approval through the DHS Program website (https://dhsprogram.com/data/available-datasets.cfm).

However, access to the DHS microdata is governed by strict data use agreements, and researchers are not permitted to publicly redistribute the raw dataset in an open repository.

To comply with PLOS ONE policy, we have:

• Revised the Data Availability Statement to clearly state controlled access via DHS

• Confirmed that the dataset can be obtained through DHS registration and approval

• Provided all derived datasets used in the analysis as Supporting Information (S1 File)

• Included all data processing and statistical analysis code to ensure full reproducibility

We respectfully request an exemption from full public deposition of the raw DHS microdata due to these contractual restrictions. However, all results are fully reproducible using the provided code once the dataset is obtained through DHS approval.

Requirement 3: Figures 5, 6, and S1 – copyright and map-related concerns

Editor Comment:

We note that Figures 5, 6, and S1 in your submission contain map images which may be copyrighted. All PLOS content is published under the Creative Commons Attribution License (CC BY 4.0), which means that the manuscript, images, and Supporting Information files will be freely available online, and any third party is permitted to access, download, copy, distribute, and use these materials in any way, even commercially, with proper attribution. For these reasons, we cannot publish previously copyrighted maps or satellite images created using proprietary data, such as Google software (Google Maps, Street View, and Earth). We require you to either (1) present written permission from the copyright holder to publish these figures under CC BY 4.0 or (2) remove/replace the figures.

Response:

Thank you for raising this important concern. We confirm that Figures 5, 6, and S1 are entirely original figures created by the authors using R (RStudio). No copyrighted maps, satellite imagery, or proprietary mapping tools such as Google Maps, Google Earth, or similar platforms were used in this study.

The spatial data used in the analysis were obtained from official DHS Program shapefiles, which are publicly available for research use. All spatial analysis and visualization were conducted independently using open-source R packages, including sf, ggplot2, and tmap.

Therefore:

• All figures are original creations of the authors

• No copyrighted or restricted map sources were used

• The figures fully comply with CC BY 4.0 licensing requirements

• No external permissions are required

Requirement 4: Supporting Information captions

Editor Comment: Please include captions for your Supporting Information files at the end of your manuscript, and update any in-text citations to match accordingly.

Response: [Page no: 41, line no:965-967]

Thank you for this comment. We have added complete captions for all Supporting Information files at the end of the revised manuscript. Additionally, all in-text citations related to Supporting Information have been carefully checked and updated to ensure full consistency with PLOS ONE formatting requirements.

RESPONSE TO REVIEWER 1 RESPONSE 1

(ABSTRACT – AGE DISAGGREGATION)

Reviewer Comment 1:

It will be helpful to have age disaggregation bands available here as otherwise it is challenging to interpret the findings.

Author Response: [Page no: 2, line no: 30, page no:11, line no: 238-239]

We sincerely thank the reviewer for this valuable suggestion. We would like to clarify that the age range of 15–49 years represents the standard reproductive age population in Demographic and Health Survey (DHS)-based studies and is used as the study eligibility criterion rather than a covariate in the analytical models. In response to the reviewer’s suggestion, we have improved clarity in the abstract methodology section by explicitly stating that the study population consists of ever-married women aged 15–49 years. In addition, we briefly indicate that age was categorized into standard DHS reproductive age groups (15–19, 20–24, 25–29, 30–34, 35–39, 40–44, and 45–49 years) for descriptive interpretation to improve understanding of the sample structure.

This revision enhances interpretability while maintaining the correct methodological distinction between study population definition and analytical covariates.

Change made:

• 15–49 defined as reproductive age study population

• Age bands added for descriptive clarity only

RESPONSE 2 (ABSTRACT – DHS VARIABLES / SCALES CLARIFICATION)

Reviewer Comment 2:

Also, it is clear that the DHS was used to gather PHQ-9 and GAD-7 data. Were additional measures also gathered from the DHS? Please specify other scales such as women’s empowerment, household asset ownership, and internet usage.

Author Response: [page no: 2, line no: 32-34, Page no: 10, line no: 216-222]

We sincerely thank the reviewer for this important comment. In response, we have clarified the abstract methodology to explicitly describe all data sources and variables used in the study.

We confirm that, in addition to PHQ-9 and GAD-7 for assessing depression and anxiety, the study also utilized multiple standardized Demographic and Health Survey (DHS)-derived variables. These included women’s empowerment indicators (based on participation in household decision-making domains), household asset ownership (constructed from ownership of selected durable goods), and internet usage (measured as self-reported use within the previous 12 months).

These variables were derived from standard DHS questionnaires and recode datasets, ensuring methodological consistency and comparability across studies.

RESPONSE TO REVIEWER 1 (COMMENT 3)

Reviewer Comment:

It would be useful to understand the percentage of comorbid depression and anxiety.

Author Response: [Page no: 32, line no: 708-710]

We sincerely thank the reviewer for this valuable suggestion. We agree that reporting comorbidity between anxiety and depressive symptoms would provide additional epidemiological insight into the mental health burden among the study population.

However, in the present analysis, comorbidity (simultaneous occurrence of PHQ-9 ≥10 and GAD-7 ≥10) was not included as a predefined outcome measure in the analytical framework. Therefore, this estimate was not reported in the current version of the manuscript.

We acknowledge this as an important limitation and have added it to the Limitations section. We also recommend that future studies using this dataset should explicitly explore comorbid mental health conditions to provide a more comprehensive understanding of mental health burden among reproductive-aged women.

RESPONSE TO REVIEWER 1 (COMMENT 4)

Reviewer 1 – Comment 4:

In your findings for the age-bands, it will be helpful to specify which group had the highest odds of anxiety, and then next highest.

Author Response [Abstract: Findings Section, Page 2-3, Lines 40-44]

We sincerely thank the reviewer for this valuable suggestion. We agree that explicitly identifying the age groups with the highest and subsequent highest odds of anxiety would improve the clarity and interpretability of the findings presented in the abstract.

In response, we revised the Findings section to clearly indicate the ranking of age groups according to the magnitude of their associations with anxiety and depressive symptoms. Specifically, women aged 35–39 years exhibited the highest odds of both anxiety (aAOR = 2.06, 95% CI: 1.48–2.87) and depressive symptoms (aAOR = 1.49, 95% CI: 1.07–2.08). Women aged 45–49 years showed the second-highest odds of anxiety (aAOR = 1.58, 95% CI: 1.12–2.23), while the second-highest odds of depressive symptoms were also observed among women aged 45–49 years (aAOR = 1.32, 95% CI: 0.92–1.89), compared with women aged 15–19 years.

These revisions improve the readability of the abstract and allow readers to more readily identify the age groups at greatest risk without comparing multiple effect estimates across categories.

RESPONSE TO REVIEWER 1 (COMMENT 5)

Reviewer 1 – Comment 5:

Suggest extending the conclusion beyond integrated policies. While this is an important start, without supportive health systems, skilled clinicians, and available services, the policies will not address MMH challenges experienced by Mozambican girls and women.

Author Response [Abstract: Conclusion Section, Page 3-4, Lines 63-66]

We sincerely thank the reviewer for this insightful and constructive suggestion. We agree that policy development alone is insufficient to effectively address maternal mental health challenges. In response, we have expanded the conclusion to emphasize the importance of supportive health systems, access to mental health services, trained healthcare professionals, and community-based interventions alongside integrated and equity-focused policies.

The revised conclusion now highlights that meaningful improvements in maternal mental health outcomes will require not only policy commitment but also adequate service availability, workforce capacity, and implementation mechanisms to ensure timely identification and support of women experiencing anxiety and depression.

This revision strengthens the public health relevance and practical implications of the study findings.

RESPONSE TO REVIEWER 1 (COMMENT 6)

Reviewer 1 – Comment 6:

Suggest ending the first sentence on line 67 after disease and starting a new sentence with “In 2021…”

Author Response [Introduction, Page 4, Lines 84-85]

We sincerely thank the reviewer for this helpful editorial suggestion. In response, we have revised the opening paragraph of the Introduction by separating the original sentence into two distinct sentences. Specifically, the first sentence now ends after “global burden of disease,” and a new sentence beginning with “In 2021…” has been introduced.

This revision improves sentence structure, readability, and overall clarity of the Introduction.

RESPONSE TO REVIEWER 1 (COMMENT 7)

Reviewer 1 – Comment 7:

Line 69…should be “worsen” and “elevate the risk of comorbid…”

Author Response [Introduction, Page 4, Line 86-87]

We sincerely thank the reviewer for this helpful language correction. In response, we have revised the sentence as suggested. The text now states that depression and anxiety “worsen quality of life, reduce productivity, and elevate the risk of comorbid physical health conditions.”

This revision improves grammatical accuracy and enhances the clarity of the statement.

Reviewer 1 – Comment 8:

Just a note that if you are including girls as young as 15 years old, so I would encourage the authors to refer to the study population as “girls and women of reproductive age”.

Author Response [Methodology, Page 10, Lines 207-208]

We sincerely thank the reviewer for this thoughtful suggestion. We acknowledge that the study population includes adolescents aged 15–19 years in addition to adult women.

However, we have retained the term “women of reproductive age” throughout the manuscript to maintain consistency with the standard terminology used in Demographic and Health Surveys (DHS), d

Attachment Submitted filename: Response_reviewr.pdf

# Editor report: Decision Letter 1

**DOI:** 10.1371/journal.pone.0356265.r003

**Authors:** Jennifer Yourkavitch

27 Jul 2026

PONE-D-25-61419R1

Stratified and Geographic Patterns of Depression and Anxiety in Mozambican Women:  Mental Health Policy Implications

PLOS One

Dear Dr. Miah,

Thank you for submitting your manuscript to PLOS ONE. After careful consideration, we feel that it has merit but does not fully meet PLOS ONE’s publication criteria as it currently stands. Therefore, we invite you to submit a revised version of the manuscript that addresses the points raised during the review process.

Please submit your revised manuscript by Sep 10 2026 11:59PM. If you will need more time than this to complete your revisions, please reply to this message or contact the journal office at plosone@plos.org. When you're ready to submit your revision, log on to https://www.editorialmanager.com/pone/ and select the 'Submissions Needing Revision' folder to locate your manuscript file.

Please include the following items when submitting your revised manuscript:

- A letter that responds to each point raised by the academic editor and reviewer(s). You should upload this letter as a separate file labeled 'Response to Reviewers'.
- A marked-up copy of your manuscript that highlights changes made to the original version. You should upload this as a separate file labeled 'Revised Manuscript with Track Changes'.
- An unmarked version of your revised paper without tracked changes. You should upload this as a separate file labeled 'Manuscript'.

If you would like to make changes to your financial disclosure, please include your updated statement in your cover letter. Guidelines for resubmitting your figure files are available below the reviewer comments at the end of this letter.

If applicable, we recommend that you deposit your laboratory protocols in protocols.io to enhance the reproducibility of your results. Protocols.io assigns your protocol its own identifier (DOI) so that it can be cited independently in the future. For instructions see: https://journals.plos.org/plosone/s/submission-guidelines#loc-laboratory-protocols. Additionally, PLOS ONE offers an option for publishing peer-reviewed Lab Protocol articles, which describe protocols hosted on protocols.io. Read more information on sharing protocols at https://plos.org/protocols?utm_medium=editorial-email&utm_source=authorletters&utm_campaign=protocols.

As the corresponding author, your ORCID iD is verified in the submission system and will appear in the published article. PLOS supports the use of ORCID, and we encourage all coauthors to register for an ORCID iD and use it as well. Please encourage your coauthors to verify their ORCID iD within the submission system before final acceptance, as unverified ORCID iDs will not appear in the published article. *Only*  the individual author can complete the verification step; PLOS staff *cannot*  verify ORCID iDs on behalf of authors.

We look forward to receiving your revised manuscript.

Kind regards,

Jennifer Yourkavitch

Academic Editor

PLOS One

Journal Requirements:

If the reviewer comments include a recommendation to cite specific previously published works, please review and evaluate these publications to determine whether they are relevant and should be cited. There is no requirement to cite these works unless the editor has indicated otherwise.

Please review your reference list to ensure that it is complete and correct. If you have cited papers that have been retracted, please include the rationale for doing so in the manuscript text, or remove these references and replace them with relevant current references. Any changes to the reference list should be mentioned in the rebuttal letter that accompanies your revised manuscript. If you need to cite a retracted article, indicate the article’s retracted status in the References list and also include a citation and full reference for the retraction notice.

Additional Editor Comments:

Thank you for your thorough edits in response to reviewers' comments. My concern is with your conclusion sections. The conclusion in the Abstract comprises general statements that do not reflect your findings. Teh conclusion section in the main narrative relies too heavily on restating results. I found some statements within the "impact on public health" section that would be good in the conclusions sections.

For example, this statement: "The findings suggest that maternal mental health should be integrated into existing reproductive, antenatal, and community health services in Mozambique." is too general and not specifically tied to your findings. One of your findings is about the higher burden on a certain age group, so perhaps interventions for that specific age group would be appropriate? That is an example of a conclusion that reflects your findings.

Please revise the conclusions sections in the abstract and in the narrative.

[Note: HTML markup is below. Please do not edit.]

To ensure your figures meet our technical requirements, please review our figure guidelines: https://journals.plos.org/plosone/s/figures

You may also use PLOS’s free figure tool, NAAS, to help you prepare publication quality figures: https://journals.plos.org/plosone/s/figures#loc-tools-for-figure-preparation.

NAAS will assess whether your figures meet our technical requirements by comparing each figure against our figure specifications.

# Author comment: Author response to Decision Letter 2

**DOI:** 10.1371/journal.pone.0356265.r004

27 Jul 2026

Thank you for the opportunity to revise our manuscript. We have carefully addressed the Academic Editor's comments and revised both the Abstract and the main Conclusion to better reflect the study findings and their public health implications. We also made additional editorial improvements throughout the manuscript, including enhancing terminology consistency, strengthening reporting clarity, updating relevant references, and improving overall readability.

A detailed point-by-point response to the Academic Editor's comments is provided in the attached document entitled "Response Letter." We have also submitted a revised manuscript with tracked changes and a clean version of the manuscript. In addition, to enhance transparency and reproducibility, the R scripts and documentation used for the analyses are publicly available in our GitHub repository: https://github.com/muhammadsalek/mozambique-mental-health-analysis.

We sincerely appreciate the Academic Editor's valuable comments and thank you for your consideration of our revised manuscript.

Attachment Submitted filename: Responses Letter.pdf

# Editor report: Decision Letter 2

**DOI:** 10.1371/journal.pone.0356265.r005

**Authors:** Jennifer Yourkavitch

2 Aug 2026

Stratified and Geographic Patterns of Depression and Anxiety in Mozambican Women:  Mental Health Policy Implications

PONE-D-25-61419R2

Dear Dr. Miah,

We’re pleased to inform you that your manuscript has been judged scientifically suitable for publication and will be formally accepted for publication once it meets all outstanding technical requirements.

Within one week, you’ll receive an e-mail detailing the required amendments. When these have been addressed, you’ll receive a formal acceptance letter and your manuscript will be scheduled for publication.

An invoice will be generated when your article is formally accepted. Please note, if your institution has a publishing partnership with PLOS and your article meets the relevant criteria, all or part of your publication costs will be covered. Please make sure your user information is up-to-date by logging into Editorial Manager at Editorial Manager® and clicking the ‘Update My Information' link at the top of the page. For questions related to billing, please contact billing support.

If your institution or institutions have a press office, please notify them about your upcoming paper to help maximize its impact. If they’ll be preparing press materials, please inform our press team as soon as possible -- no later than 48 hours after receiving the formal acceptance. Your manuscript will remain under strict press embargo until 2 pm Eastern Time on the date of publication. For more information, please contact onepress@plos.org.

Kind regards,

Jennifer Yourkavitch

Academic Editor

PLOS One

# Editor report: Acceptance letter

**DOI:** 10.1371/journal.pone.0356265.r006

**Authors:** Jennifer Yourkavitch

PONE-D-25-61419R2

PLOS One

Dear Dr. Miah,

I'm pleased to inform you that your manuscript has been deemed suitable for publication in PLOS One. Congratulations! Your manuscript is now being handed over to our production team.

At this stage, our production department will prepare your paper for publication. This includes ensuring the following:

* All references, tables, and figures are properly cited

* All relevant supporting information is included in the manuscript submission,

* There are no issues that prevent the paper from being properly typeset

You will receive further instructions from the production team, including instructions on how to review your proof when it is ready. Please keep in mind that we are working through a large volume of accepted articles, so please give us a few days to review your paper and let you know the next and final steps.

Lastly, if your institution or institutions have a press office, please let them know about your upcoming paper now to help maximize its impact. If they'll be preparing press materials, please inform our press team within the next 48 hours. Your manuscript will remain under strict press embargo until 2 pm Eastern Time on the date of publication. For more information, please contact onepress@plos.org.

You will receive an invoice from PLOS for your publication fee after your manuscript has reached the completed accept phase. If you receive an email requesting payment before acceptance or for any other service, this may be a phishing scheme. Learn how to identify phishing emails and protect your accounts at https://explore.plos.org/phishing.

If we can help with anything else, please email us at customercare@plos.org.

Thank you for submitting your work to PLOS ONE and supporting open access.

Kind regards,

PLOS ONE Editorial Office Staff

on behalf of

Dr. Jennifer Yourkavitch

Academic Editor

PLOS One
